# Loss of RPGR disrupts motile cilia and causes primary ciliary dyskinesia by affecting F-actin dynamics

**DOI:** 10.1172/JCI193367

**Published:** 2026-03-31

**Authors:** Yang Wu, Erika Tavares, Binrun Liang, Wallace B. Wee, Vito Mennella, Han-Chao Feng, Jiaying Cao, Pui Yee Wong, Jiayi Zheng, Mu He, Kirk AJ Stephenson, Liran Hanan Hochma, Janice Min Li, Nan-Peng Chen, Sharon D. Dell, Elise Heon, Zhen Liu

**Affiliations:** 1 Department of Life Science, The Hong Kong University of Science and Technology, Hong Kong SAR, China.; 2 Genetics & Genome Biology Program, and; 3 Child Health Evaluative Sciences, The Hospital for Sick Children, Toronto, Canada.; 4 Department of Biochemistry, School of Biological and Behavioural Sciences, Queen Mary University of London, London, United Kingdom.; 5 School of Biomedical Sciences, The University of Hong Kong, Hong Kong SAR, China.; 6 Department of Ophthalmology and Vision Sciences, The Hospital for Sick Children, University of Toronto, Toronto, Canada.; 7 Institute of Systems and Physical Biology, Shenzhen Bay Laboratory, Shenzhen, China.; 8 Division of Respiratory Medicine, BC Children’s Hospital, The University of British Columbia, Vancouver, Canada.

**Keywords:** Cell biology, Pulmonology, Cytoskeleton, Diagnostic imaging, Respiration

## Abstract

Cilia are cellular organelles that extrude from the surface of various cell types, serving either sensory or motile functions. Retinitis pigmentosa GTPase regulator (*RPGR*) variants affect both photoreceptor sensory cilia and airway motile cilia, leading to retinitis pigmentosa (RP) and primary ciliary dyskinesia (PCD), respectively. Not all patients develop PCD, and it remains unclear which *RPGR* variants predispose patients to PCD. Here, we leverage 2D organoids, super-resolution microscopy, and live-cell imaging to characterize the multiciliated cells (MCCs) from patients with different *RPGR* variants and CRISPR-modified *RPGR* KO MCCs. We demonstrate that MCCs with *RPGR* variants have reduced ciliation, shorter cilia, impaired cilia beat, or cilia beat incoordination, potentially resulting in compromised mucociliary clearance and lung diseases. Moreover, we show that RPGR regulates motile cilia through interfering with F-actin dynamics, evidenced by the undissolved F-actin meshwork in *RPGR*-deficient MCCs, and the defects can be ameliorated with either latrunculin A or Y27632 treatment. Though PCD was observed only in patients with variants that affect both isoforms, patients with *RPGR^ORF15^* variants also had cilia and airway anomalies. All *RPGR* variants affected motile cilia in some way, and the mechanisms involved the accumulation of apical F-actin.

## Abstract 2

Loss of RPGR, through apical F-actin accumulation in airway cells, leads to reduced ciliation with short cilia that have an impaired beat, leading to defective mucociliary clearance.

## Introduction

**Figure 1:**
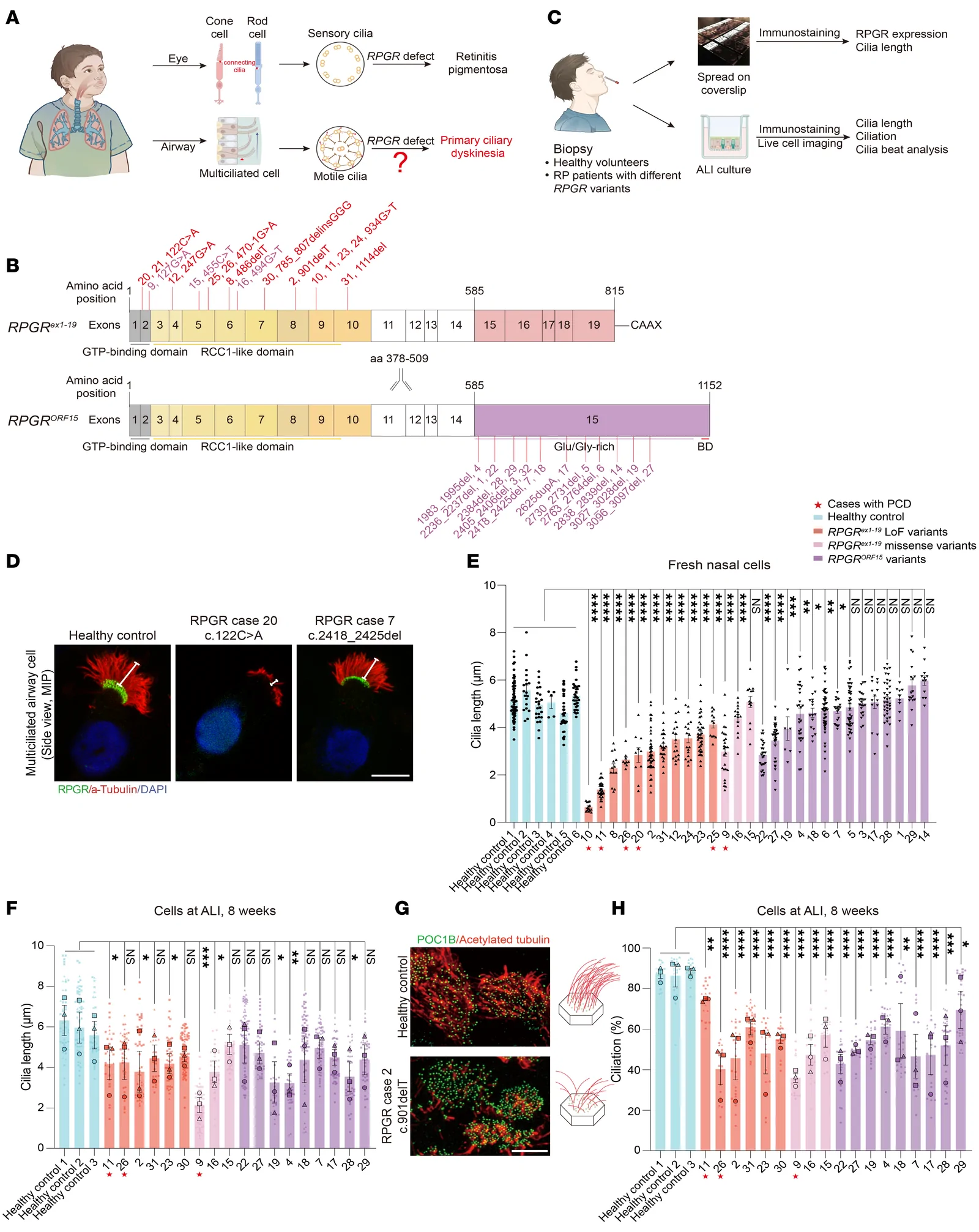
MCCs with pathological *RPGR* variants presented with short cilia and decreased ciliation. (**A**) Cartoon showing *RPGR* as the causative gene for RP and PCD, as well as the focus of this study. (**B**) Distributions of variants of the 32 patients participating in this study. Red indicates variants that affect both isoforms and correspond to LoF or splicing; pink corresponds to variants with missense effect; and purple corresponds to variants affecting only *RPGR^ORF15^*. Variants in patients 9 and 15 are predicted to be missense and affect splicing. (**C**) The proposed workflow for this study. (**D**) Immunostaining of human nasal cells of control individuals and patients with *RPGR* variants. Green represents RPGR and red represents α-tubulin. MIP, maximum intensity projection. Scale bar: 10 μm. (**E**) Cilia length measurements for the nasal MCCs from control participants and patients with RP. Individuals with PCD are distinguished with a red asterisk. (**F**) Cilia length measurements for the MCCs from control participants and patients with RP cultured at the ALI for 8 weeks. (**G**) Immunostaining of basal body (POC1B) and cilia (acetylated tubulin) showed reduced ciliation in the MCCs of a patient. Scale bar: 5 μm. (**H**) Ciliation was reduced for the MCCs bearing pathological *RPGR^ex1–19^* or *RPGR^ORF15^* variants. Data represent mean ± SEM. *P < 0.05, **P < 0.01, ***P < 0.001, ****P < 0.0001 by 2-tailed *t* test (**E**, **F**, and **H**). For (**E**), we performed 1 experiment in a subset of the cohort that included 6 healthy control participants and 28 patients. For (**F**–**H**), we performed 3 independent replicates for a subset of the cohort that included 3 healthy control participants and 18 patients.

Cilia are highly conserved organelles that protrude from the cell surface and are essential for cellular sensing, signaling, or motility (1, 2). Cilia can generally be classified into motile cilia or sensory (primary) cilia (3), based on their structure and whether they beat (Figure 1A). Motile cilia align along the surface of the respiratory tract, ependyma, and fallopian tubes; beat in synchrony; and function in airway mucociliary clearance, cerebral fluid circulation, and egg delivery (1). The sensory cilia are solitary and appear on nearly all cells, receiving environmental stimuli and eliciting signaling pathways, such as Hedgehog signaling (4). The photoreceptor outer segment is a specialized sensory cilium dedicated to phototransduction, a process key to vision (5).

Conditions due to defective function, development, or maintenance of cilia are collectively referred to as ciliopathies. Primary ciliary dyskinesia (PCD) is an important motile ciliopathy (6, 7), affecting approximately 1 in 7,500 people worldwide (8). Major features of PCD include oto-sino-pulmonary diseases, which can be life-threatening because of the progressive deterioration of lung function. Retinitis pigmentosa (RP) is a genetically heterogeneous disorder characterized by degeneration of photoreceptors, leading to night blindness and sight loss (9). The incidence rate of RP ranges between 1 in 3,000 to 4,000 people (10). RP is linked to various gene variants related to the visual pathway, and variants associated with sensory cilia are known to contribute to RP (11).

Deleterious variants involving the retinitis pigmentosa GTPase regulator (*RPGR*) gene account for approximately 70% of X-linked RP (12–14) and are also a known cause of X-linked PCD (15). *RPGR* is expressed in sensory and motile cilia (16, 17). As a result, patients with *RPGR* deleterious variants could present with both motile and sensory ciliopathy phenotypes (Figure 1A). *RPGR* is expressed in 2 major isoforms (Figure 1B) (18–21): one is constitutively expressed in multiple organs and cell types (NM_000328.3; *RPGR^ex1–19^*), and the other has a retina-specific expression pattern (NM_001034853.2; *RPGR^ORF15^*). *RPGR^ex1–19^* has 19 exons, of which *RPGR^ORF15^* shares exons 1–14 (18, 22–25). *RPGR^ORF15^* is an X-linked RP mutational hotspot, whereas *RPGR^ex1–19^* is ubiquitously expressed and was initially regarded as the sole isoform in airway multiciliated cells (MCCs) (17, 18). In the retina, RPGR contributes to outer-segment membrane disk renewal, and variants involving either *RPGR^ex1–19^* or *RPGR^ORF15^* cause early-onset RP by disrupting actin dynamics (26).

RPGR’s role in motile cilia remains unclear. Whereas most PCD causative genes encode axoneme components, dynein arm docking or assembly factors, or transcription factors that regulate multiciliogenesis, RPGR is distinct and may play a role in ciliary transport (27–30). The gold standard for confirming PCD diagnosis is either ciliary structural defects detected by transmission electron microscopy (TEM) and/or biallelic variants in known PCD genes detected by DNA sequencing. However, these standards cannot diagnose all PCD cases, including *RPGR* variants, as some *RPGR* variants could be variants of unknown significance or may not lead to classic TEM changes (31, 32). Previous clinical reports showed that variants in *RPGR* associated with PCD can result in static cilia, impaired cilia beat, or disrupted beat coordination (15, 19, 33). However, these reports were based on a limited number of patients. Which variants predispose patients to PCD and how RPGR regulates motile cilia properties are still unclear.

Using super-resolution microscopy and live-cell imaging of patient samples, we observed that patients with pathological variants affecting both isoforms (*RPGR^ex1–19^* variants) had sparse, shorter, and mostly static motile cilia. For *RPGR^ORF15^* variants, shorter cilia were observed for a portion of patients, and the ciliation level was decreased for all patients. Most MCC cilia with *RPGR^ORF15^* variants either exhibited restricted motility or were static. These phenotypes were recapitulated in CRISPR-mediated *RPGR* KO MCCs.

Investigating the mechanisms underlying how RPGR regulates motile cilia revealed its role in regulating F-actin dynamics. With *RPGR* variants, a condensed F-actin meshwork accumulates and persists at the apical surface of MCCs, preventing some cilia from extruding from the surface and restricting their movement on the cell surface. This persistence of F-actin meshwork is associated with a temporary reduction in gelsolin levels at the apical surface. To examine whether the phenotype was specific to F-actin changes, we treated *RPGR* KO MCCs and patient MCCs with pathological *RPGR* variants with either the actin polymerization inhibitor latrunculin A (LatA) or the ROCK inhibitor Y27632, and we found that cilia length and ciliation level were ameliorated, and cilia beat properties were partially restored.

To summarize, working with a cohort of 32 patients with different *RPGR* variants, we provide the first mechanistic study, to our knowledge, of RPGR’s function in the respiratory system. Although PCD was more prevalent in patients with *RPGR^ex1–19^* variants, cilia anomalies were seen in patients with both *RPGR^ex1–19^* and *RPGR^ORF15^* variants, and patients with *RPGR^ORF15^* variants had frequent respiratory findings, though these were not characteristic of PCD. This work also reveals a distinct role of *RPGR* compared with that of other genes associated with PCD: RPGR modulates F-actin dynamics at the apical surface to control multiciliogenesis and regulate ciliary beat machinery. The methods reported here can be translated clinically to identify individuals at risk of mucociliary clearance defects and improve patient outcomes, with appropriate management.

## Results

### Patient characterization.

This study was approved by the ethics board of participating institutions, and recruitment respected the tenets of the Declaration of Helsinki. We recruited a cohort of 32 patients with X-linked RP with a wide range of *RPGR^ex1–19^* and *RPGR^ORF15^* variants, including 1 female patient with retinal degeneration (Figure 1, B and C, and Supplemental Table 1; supplemental material available online with this article; https://doi.org/10.1172/JCI193367DS1), and 12 healthy volunteers. All patients were recruited through eye clinics and had a disease-associated *RPGR* variant and retinal degeneration. The predicted impact of each identified variant was validated using literature, pathogenicity and conservation predictive algorithms, allele frequency databases, and splicing assays (34–39) (Supplemental Table 1 and Supplemental Figure 1). *RPGR^ex1–19^* variants were found in 16 patients: 8 were considered loss of function (LoF), 5 were missense resulting in an in-frame deletion, 2 had missense variants with loss of transcript due to incomplete splicing (missense splicing), and 1 had a missense. LoF variants affecting the *RPGR^ORF15^* isoform were present in the remaining 16 patients (Supplemental Table 1).

Our assessment of the 32 patients with either *RPGR^ex1–19^* or *RPGR^ORF15^* variants revealed a range of respiratory manifestations: 19 of 31 (61%) had sinus diseases, 15 of 30 (50%) had lower airway diseases, and 19 of 31 patients (61%) had hearing issues, including hearing loss and/or otitis media (Supplemental Table 2). Of the 16 patients carrying *RPGR^ORF15^* variants, 5 (31%) had sinus disease, 4 (25%) had lower airway disease, and 8 (50%) had hearing issues.

Additional clinical assessments were performed, and these indicated 9 of 28 patients (32%) had abnormal lung function with obstructive ventilation defects (*n* = 7 mild, 1 moderate, and 1 severe defect). Lung-imaging airway abnormalities were present on either chest radiographs or computed tomography scan in 11 of 27 patients (41%), of whom 6 had bronchiectasis. Of the 16 patients carrying *RPGR^ORF15^* variants, 2 (13%) had abnormal lung function, and 3 (19%) had abnormal chest radiographs.

Combining the results from all assessments, 6 patients (19%) met criteria for a clinical diagnosis of PCD. All exhibited symptoms of severe oto-sino-pulmonary disease with symptom onset in early childhood, with reduced nasal nitric oxide levels and airway abnormalities on chest imaging, and all of whom had *RPGR^ex1–19^* variants. Only 2 of the 6 patients were diagnosed with PCD prior to being recruited into our study.

### Patients with pathological RPGR^ex1–19^ or RPGR^ORF15^ variants had cilia defects.

Fresh nasal cilia were available from 29 patients. The mean age of patients at the time of nasal biopsy was 30 years (range 8–63 years). Confocal microscopy of fresh MCCs immunostained with RPGR antibodies and antibodies targeting cilia showed that RPGR was absent in most patients with *RPGR^ex1–19^* variants but was properly localized in the cells bearing *RPGR^ORF15^* variants (Figure 1D). Cilia length measurements showed that 13 of 14 patients (93%) with *RPGR^ex1–19^* variants, and 7 of 14 patients (50%) with *RPGR^ORF15^* variants had decreased cilia length (Figure 1E). This suggests that overall RPGR is important for motile cilia growth or length regulation.

Analysis of MCCs from a female patient (case 21) with severe RP due to an *RPGR^ex1–19^* variant [c.122C>A; p.(Ser41*)] showed that half of her MCCs did not express RPGR and they had shorter cilia because of unfavorable X-inactivation (Supplemental Figure 2A). Because fresh nasal cells might be subject to infection and other environmental stress, we redifferentiated the isolated basal cells at the air-liquid interface (ALI) for further assessment. Differentiated cells were available from 18 patients. Cilia length of the differentiated MCCs at 4 and 8 weeks was measured by analyzing the signal from acetylated tubulin (Figure 1F and Supplemental Figure 2B). Of 18 patients, 5 with *RPGR^ex1–19^* variants and 3 with *RPGR^ORF15^* variants had cells with significantly shorter motile cilia than did control individuals (Figure 1F). Shorter cilia were more prevalent for 4-week MCCs in 8 of 9 patients with *RPGR^ex1–19^* variants and in 8 of 9 patients with *RPGR*^ORF15^ variants (Supplemental Figure 2B). The shorter cilia in MCCs bearing *RPGR^ORF15^* variants suggest that *RPGR^ORF15^* also contributes to the growth of the motile cilia. ALI cell culture also enabled us to quantify the ciliation level, defined as the number of cilia divided by the number of basal bodies per MCC (Figure 1, G and H, and Supplemental Figure 2C). Patients with either *RPGR^ex1–19^* (*n* = 9 of 9) or *RPGR^ORF15^* (*n* = 9 of 9) variants had significantly decreased ciliation level compared with healthy control individuals (Figure 1H).

Altogether, our characterization of the MCCs from patients with a wide variety of pathogenic and likely pathogenic *RPGR^ex1–19^* and *RPGR^ORF15^* variants shows that disruption of RPGR function may lead to reduced ciliation and shortened cilia length.

### Damaging RPGR^ex1–19^ and RPGR^ORF15^ variants affect cilia beat.

**Figure 2:**
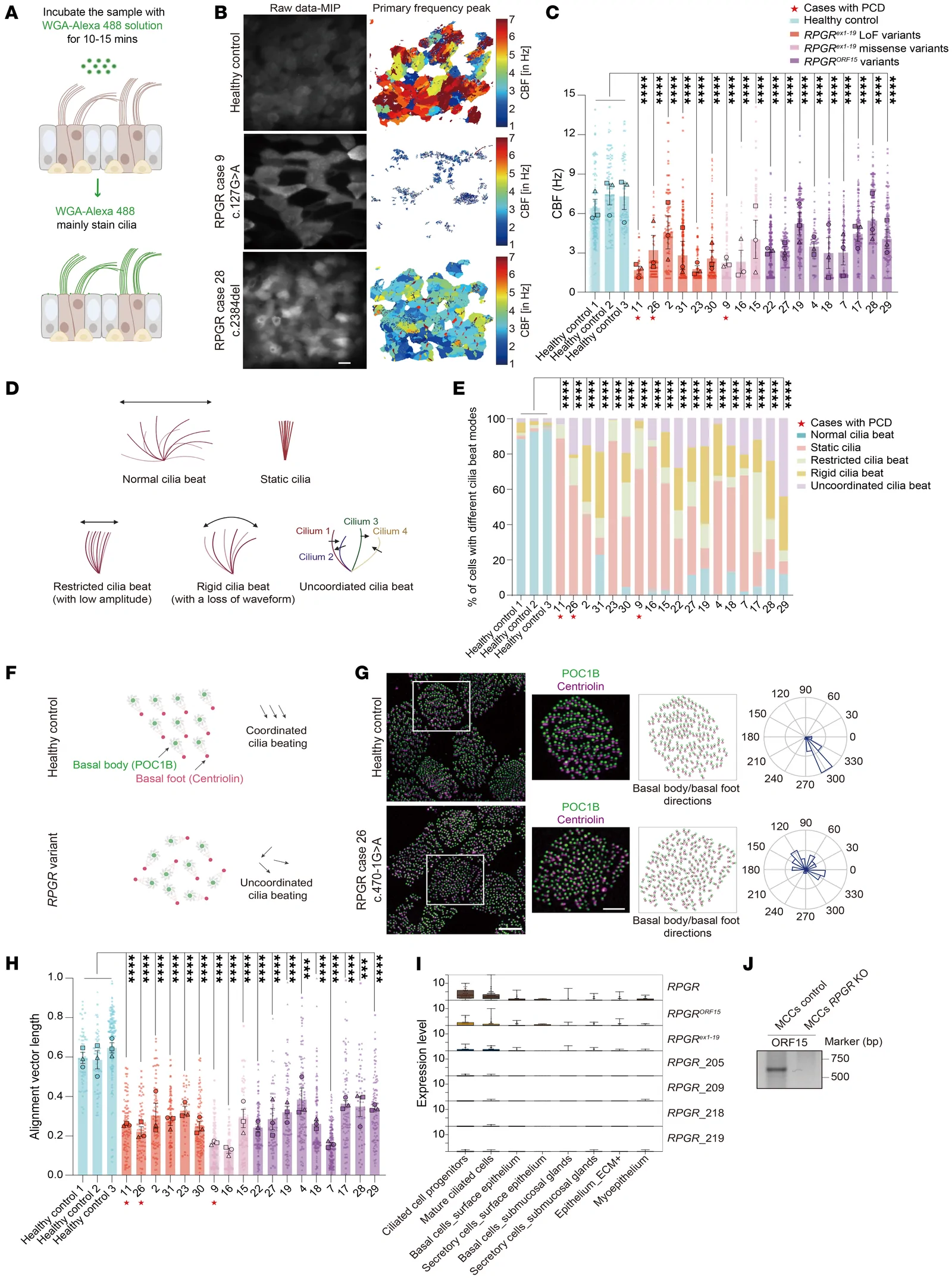
MCCs with *RPGR* pathological variants had an impaired and uncoordinated cilia beat. (**A**) A cartoon showing labeling motile cilia with wheat germ agglutinin–Alexa 488 (WGA-Alexa 488). (**B**) The pixel frequency map shows cilia beat impairment in the MCCs with pathological *RPGR* variants. MIP, maximum intensity projection. CBF, cilia beat frequency. Scale bar: 10 μm. (**C**) Characterization of CBF in cells from 18 patients with RP in this study. (**D**) Different cilia beat modes identified in *RPGR* LoF MCCs. (**E**) Characterization of cilia beat waveform distribution for 18 patients with RP in this study. (**F**) A cartoon showing labeling POC1B and centriolin to assess rotational polarity and its disruption in MCC bearing a pathological *RPGR* variant. (**G**) Immunostaining shows rotational polarity in healthy control cells and its disruption in 1 patient with a pathological *RPGR^ex1–19^* variant. Scale bars: 2 μm; insert, 2 μm. (**H**) Characterization of rotational polarity in cells from 18 patients with RP in this study. (**I**) Single-cell RNA-Seq revealed the expression of *RPGR* mRNA and its different isoforms in airway epithelial cells. ECM, extracellular matrix. (**J**) RT-PCR showed the expression of the *RPGR^ORF15^* isoform in human airway epithelial cells (HNCs cultured at ALI for >8 weeks). Data represent mean ± SEM. The box shows the 25th–75th percentile, with the middle line showing the median (**I**). ***P < 0.001, ****P < 0.0001 by 2-tailed *t* test (**C**, **E**, and **H**). For (**C**, **E**, and **H**), we performed 3 technical replicates for a subset of the cohort that included cells from 3 healthy control individuals and 18 patients.

To investigate how RPGR regulates cilia motility, cilia beating was measured for MCCs redifferentiated at ALI for 8 weeks. We stained the whole filter with wheat germ agglutinin–Alexa Fluor 488 to mark the highly glycosylated motile cilia (40) and imaged the MCCs with an inverted fluorescence microscope (Figure 2A). The frequency of pixel signal fluctuations in Supplemental Video 1 was initially used to represent the overall cilia motility within the field of interest. This method clearly distinguishes 2 patients from a healthy control individual in the frequency map (Figure 2B). To quantify the differences, we further measured the cilia beat frequency in individual cells from patients with different variants (Supplemental Figure 3). Our analysis showed that most motile cilia from patients with *RPGR^ex1–19^* variants were static or had a restricted beat (*n* = 7 of 9; 78%), whereas for patients 2 and 15, a mixture of slow and fast beat cilia was observed (Figure 2C). All patients with variants in *RPGR^ORF15^* (*n* = 9 of 9) had decreased cilia beat frequency (Figure 2C).

The motile cilia beating of healthy control cells had a distinguishable power and recovery stroke, as well as synchronization of the metachronal wave (Supplemental Video 1). On the contrary, MCCs from patients with RP showed noticeable heterogeneity in those features, between patients as well as among cells from the same patient (Supplemental Video 1). Regarding the cilia beat waveform, besides the normal cilia beat, 4 different types of beat phenotypes could be identified: (a) static cilia, (b) restricted cilia beat (low amplitude), (c) rigid cilia beat with a loss of waveform, and (d) cilia beat in a rotational and/or uncoordinated manner (Figure 2D). We quantified the percentages of cells exhibiting different cilia beat modes and found that most cells with either *RPGR^ex1–19^* or *RPGR^ORF15^* variants displayed static cilia or a restricted cilia beat (*n* = 8 of 9 [89%] for *RPGR^ex1–19^* and 6 of 9 (67%) for *RPGR^ORF15^* variants), whereas the rest (*n* = 1 of 9 [11%] for *RPGR^ex1–19^* and *n* = 3 of 9 [33%] for *RPGR^ORF15^* variants) had a rigid or uncoordinated beat (Figure 2E).

To quantitatively compare beat coordination, we used a 3D structured illumination microscopy–based (3D-SIM–based) method we previously developed (41) to examine the rotational polarity of the basal foot (Figure 2, F and G), an appendage structure localized at the base of each cilium (41, 42). In healthy MCCs, all basal feet point toward the direction of cilia beat, a phenomenon called rotational polarity (42–44) (Figure 2F), which is disrupted in patients with PCD, because of defective cilia beat (41). We performed this analysis and found that the rotational polarity was disrupted in cells with either *RPGR^ex1–19^* (*n* = 9 of 9) or *RPGR^ORF15^* (*n* = 9 of 9) variants (Figure 2H).

Considering all results together, damaging variants in *RPGR^ex1–19^* and *RPGR^ORF15^* affect airway MCC cilia beat frequency, waveform, and coordination.

### Patients with RPGR-RP with severe PCD symptoms had severe cilia defects.

Patients with severe clinical respiratory PCD symptoms had *RPGR^ex1–19^* LoF variants or in-frame deletions and had severe cilia defects. All 6 patients whose condition met the clinical diagnostic criteria of PCD had shortened nasal cilia length (Figure 1E). We successfully redifferentiated cells from 3 patients (patients 11, 26, and 9); 2 had cells with reduced cilia length and ciliation, and the third had cells with slowed cilia growth, supporting our findings from fresh cells (Figure 1, F and H, and Supplemental Figure 2B). Cilia beat analysis of MCCs from those same 3 patients with PCD showed that most MCCs presented with static cilia or a restricted cilia beat, and the rotational polarity was disrupted in all cases (Figure 2, C, E, and H, and Supplemental Figure 4A).

Four patients with RP bearing the same *RPGR^ex1–19^* variant [c.934G>T; p.(Glu260_Thr311del)] presented with different clinical and cellular phenotypes: 2 patients presented with a clinical PCD phenotype and shorter nasal cilia length, and patients 23 and 24 only had milder respiratory symptoms and intermediate nasal cilia lengths (Figure 1E, Supplemental Figure 4B, and Supplemental Table 2). This suggests multifactorial influences in the development of PCD.

While most patients with *RPGR*-RP had motile cilia defects, only a subpopulation presented with a distinctly abnormal respiratory phenotype, with 6 patients showing a strong PCD phenotype and bearing variants affecting both isoforms. However, in cases of patients with *RPGR^ORF15^* variants, abnormal cilia and respiratory phenotypes were also observed, suggesting these patients may be affected by respiratory illnesses caused by cilia dysfunction (Figures 1 and 2, Supplemental Figure 4C, and Supplemental Table 2).

### RPGR^ORF15^ isoform was expressed in airway MCCs.

Although the *RPGR^ex1–19^* isoform was once regarded as the sole isoform in airway MCCs (17), we showed that cells from patients with *RPGR^ORF15^* variants presented with cilia defects (Figure 1, E, F, and H), albeit with less severe respiratory phenotypes (Supplemental Table 2). To validate if the *RPGR^ORF15^* isoform is expressed in MCCs, we first examined the single-cell sequencing data of a fetus’s trachea generated in a previous study by He et al. (45). All *RPGR* isoforms were first treated as a single gene. At the mRNA level, compared with other cell types, *RPGR* was mainly expressed in the MCC progenitors (Foxn4 expressing) and mature MCCs (Figure 2I). We next separated different isoforms and performed isoform-specific expression analysis. The result showed that the expression levels of the *RPGR^ORF15^* isoform were comparable to the predominantly expressed *RPGR^ex1–19^* isoform, confirming that *RPGR^ORF15^* is expressed in airway cells. The expression of the *RPGR^ORF15^* isoform was upregulated during the early stage of multiciliogenesis (Figure 2I), indicating its role in cilia formation. To further examine the expression of *RPGR^ORF15^* isoform in MCCs, we performed RT-PCR of the total mRNA transcripts extracted from healthy control cells and *RPGR* KO MCCs. The result further supports that *RPGR^ORF15^* is expressed in airway cells (Figure 2J and Supplemental Figure 5), consolidating the single-cell sequencing data.

### RPGR KO MCCs presented with sparse and short motile cilia.

**Figure 3:**
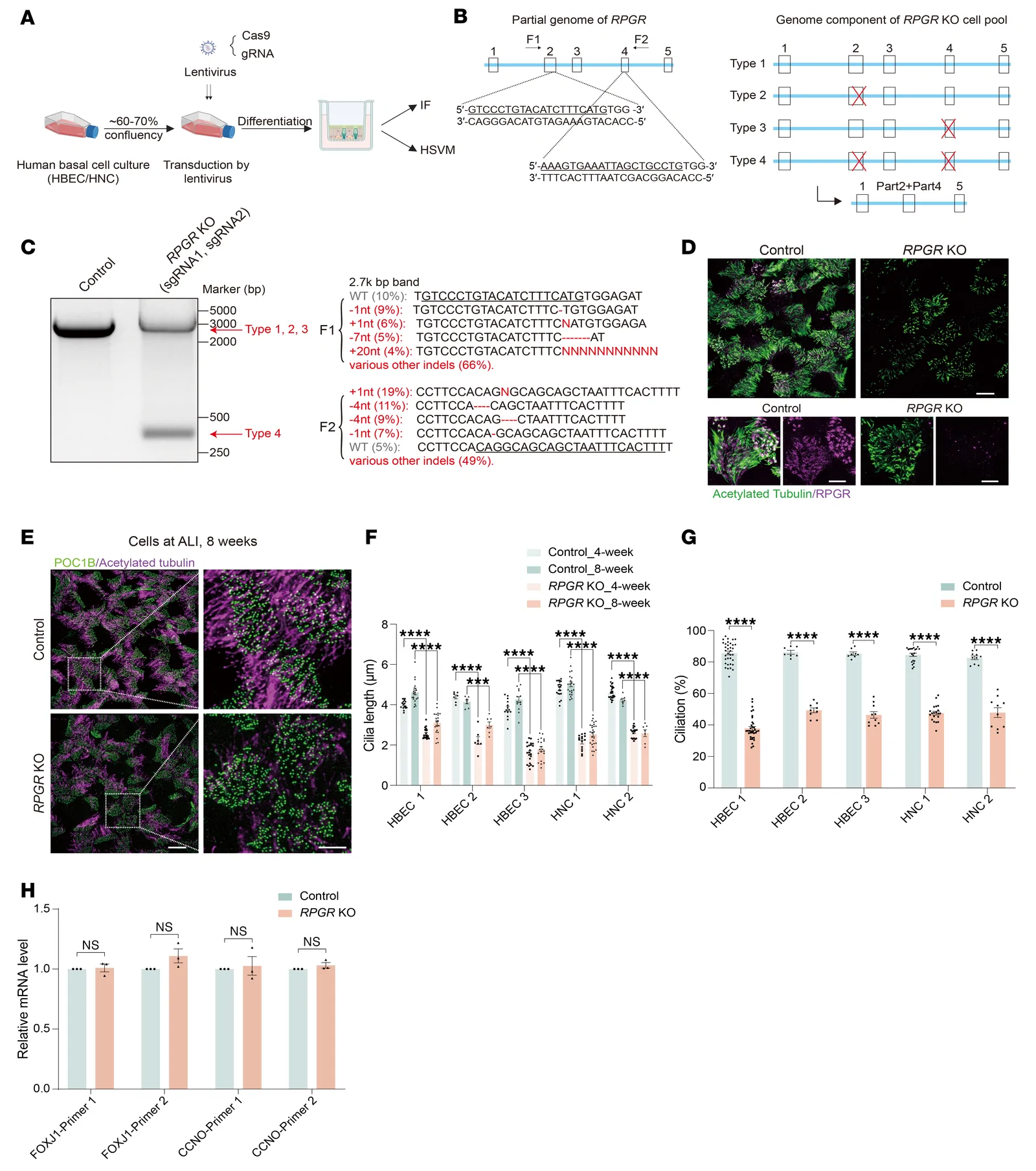
*RPGR* KO MCCs presented with sparse and short motile cilia. (**A**) Workflow of generating *RPGR* KO MCCs. Indel, insertion-deletion. HSVM, high-speed video microscopy. (**B**) The location of gRNAs and the expected genome edits. (**C**) DNA gel and Sanger sequencing showed high efficiency of *RPGR* KO in MCCs. (**D**) Immunostaining of MCCs with RPGR antibody showed complete RPGR removal in *RPGR* KO MCCs. Scale bars: 10 μm; insert, 5 μm. (**E**) Immunostaining with POC1B and acetylated-tubulin antibodies show the reduction of ciliation length and ciliation for *RPGR* KO HBEC/HNC cells. Scale bars: 10 μm; insert, 5 μm. (**F**) Cilia length was significantly disrupted in all 5 biological replicates. (**G**) Ciliation was severely affected for all 5 *RPGR* KO biological replicates. (**H**) *FOXJ1* and *CCNO* expressions were unaffected in *RPGR* KO MCCs. For each gene, 2 sets of primers were used. Data represent mean ± SEM. ***P < 0.001, ****P < 0.0001 by 2-way repeated ANOVA followed by Šídák’s post hoc test (**F**) or 2-tailed *t* test (**G** and **H**). For (**F** and **G**), we performed 5 experiments using cells from 5 control individuals and corresponding *RPGR* KO MCCs. For (**H**), we performed 3 experiments using cells from 3 control individuals and corresponding KO MCCs.

To determine the *RPGR* specificity of our findings, we generated *RPGR* KO MCCs by CRISPR-mediated genomic perturbation of healthy human nasal or bronchial basal cells. By using an optimized protocol that includes medium adjustments and adaptation of 2 sgRNAs simultaneously (Figure 3, A and B), we successfully produced *RPGR* KO MCCs, as confirmed by DNA gel electrophoresis and Sanger sequencing (Figure 3C). The percentage of cells in which both gRNAs were effective reached 92.3%, resulting in an overall KO efficiency of approximately 99% (Figure 3C). Immunostaining showed a complete absence of RPGR compared with the control (Figure 3D). We further characterized the cilia properties of 5 biological replicates, including 3 human bronchial epithelial cell (HBEC) and 2 human nasal cell (HNC) samples, showing that cilia length was affected in all (Figure 3, E and F). Quantification of the ciliation level of MCCs showed that, although basal body number was unaffected, ciliation was significantly decreased (Figure 3, E and G, and Supplemental Figure 2D), which suggests that RPGR regulates ciliation but not basal body amplification or docking. To rule out the possibility that the phenotype was due to a secondary defect in MCC differentiation, we showed the expression level of *FOXJ1* and *CCNO*, as assessed by qRT-PCR, was unchanged in *RPGR* KO MCCs (*n* = 3) (Figure 3H).

Taken together, removing *RPGR* by CRISPR perturbation from airway MCCs led to shortened cilia and reduced ciliation levels, recapitulating the cellular phenotypes observed in the patients.

### RPGR KO MCCs presented with static cilia and/or abnormal cilia motility.

**Figure 4:**
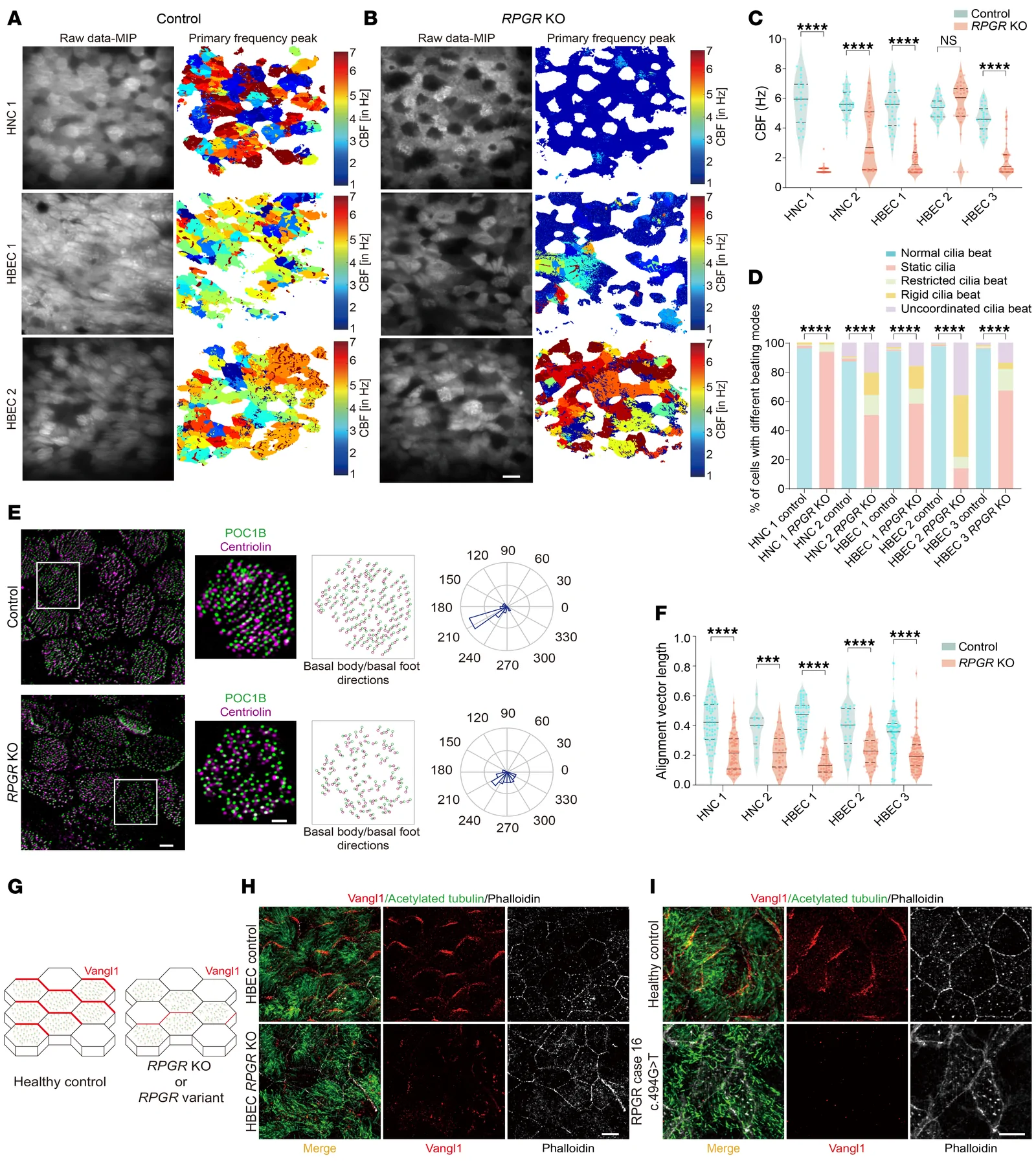
*RPGR* KO MCCs presented with limited motility and defective rotational and planar polarity. (**A** and **B**) The pixel frequency map shows control MCC cilia beat and its impairment in *RPGR* KO MCCs. CBF, cilia beat frequency; MIP, maximum intensity projection. Scale bar: 10 μm. (**C** and **D**) Characterization of CBF and beat mode changes for 5 biological replicates in this study. (**E**) Immunostaining showed rotational polarity in control cells and its disruption in *RPGR* KO MCCs. Scale bars: 2 μm; insert, 1 μm. (**F**) Rotational polarity was disrupted for all *RPGR* KO samples in this study. (**G**–**I**) Vangl1 is mislocalized in the MCCs from an HBEC *RPGR* KO sample and from a sample from a patient with RP, suggesting loss of planar polarity. Scale bar: 5 μm. Data represent mean ± SEM. The center, upper, and lower lines represent the median, upper, and lower quartiles, respectively (**C** and **F**). ***P < 0.001, ****P < 0.0001 by 2-tailed *t* test (**C** and **F**) or Fisher’s exact test (**D**). For (**C**, **D**, **F**, and **H**), we performed 5 experiments using cells from 5 control individuals and corresponding KO MCCs. (**I**) Results from 9 of 10 patients (see Supplemental Figure 2F for details).

Live-cell imaging of *RPGR* KO MCCs (Figure 4, A–D) showed that most cells (68%; *n* = 4 of 5 biological replicates) had static cilia (Figure 4, B, upper and middle, and Supplemental Video 2), leading to disrupted fluorescent bead clearance in vitro (Supplemental Video 3). There was large heterogeneity within and among different biological replicates (Figure 4C). A quantification of cilia beat frequency of the *RPGR* KO MCCs showed results consistent with patient data. Analysis of the cilia beat waveform, in 4 of 5 replicates, showed static cilia were predominant; for the fifth replicate, although most cilia could beat with a normal frequency compared with the control (NS; *n* = 2 technical replicates), both the waveform and beat coordination were abnormal (Figure 4D).

The rotational polarity assessment of *RPGR* KO cells showed a drastic disruption of beat coordination for all biological replicates (*n* = 5) (Figure 4, E and F). Previous studies suggested the disruption of planar cell polarity (PCP) for patient cells bearing *RPGR* variants (46, 47); therefore, we stained the ALI filters with antibodies targeting the planar polarity protein Vangl1 (48, 49). For control cells, Vangl1 was specifically enriched on 1 side of the MCCs. In both *RPGR* KO HNCs and HBECs, as well as in patient cells with *RPGR^ex1–19^* or *RPGR^ORF15^* variants, the asymmetrical distribution of Vangl1 was largely lost, suggesting a disruption in planar polarity (Figure 4, G–I, and Supplemental Figure 2, E and F).

Furthermore, we designed a gRNA that specifically targets the *RPGR* ORF15 region and achieved a KO efficiency of 70% in HBECs and 59% in HNCs (Supplemental Figure 6A). Characterization of the MCCs generated with the ORF15-specific gRNA revealed that cilia length, ciliation, cilia beat frequency, waveform, and coordination were all affected, as was the distribution of Vangl1 (Supplemental Figure 6, B–H). These changes impaired the clearance of fluorescent beads and recapitulated the phenotypes of the patient MCCs bearing *RPGR^ORF15^* variants (Supplemental Figure 6I).

To summarize, RPGR defects caused by CRISPR perturbation led to either static cilia or cilia with altered motility, disrupting both planar and rotational polarity.

### RPGR localizes to the transition zone and cilia membrane; its loss does not affect the integrity of the transition zone or the localization of axoneme dyneins and central pair components.

**Figure 5:**
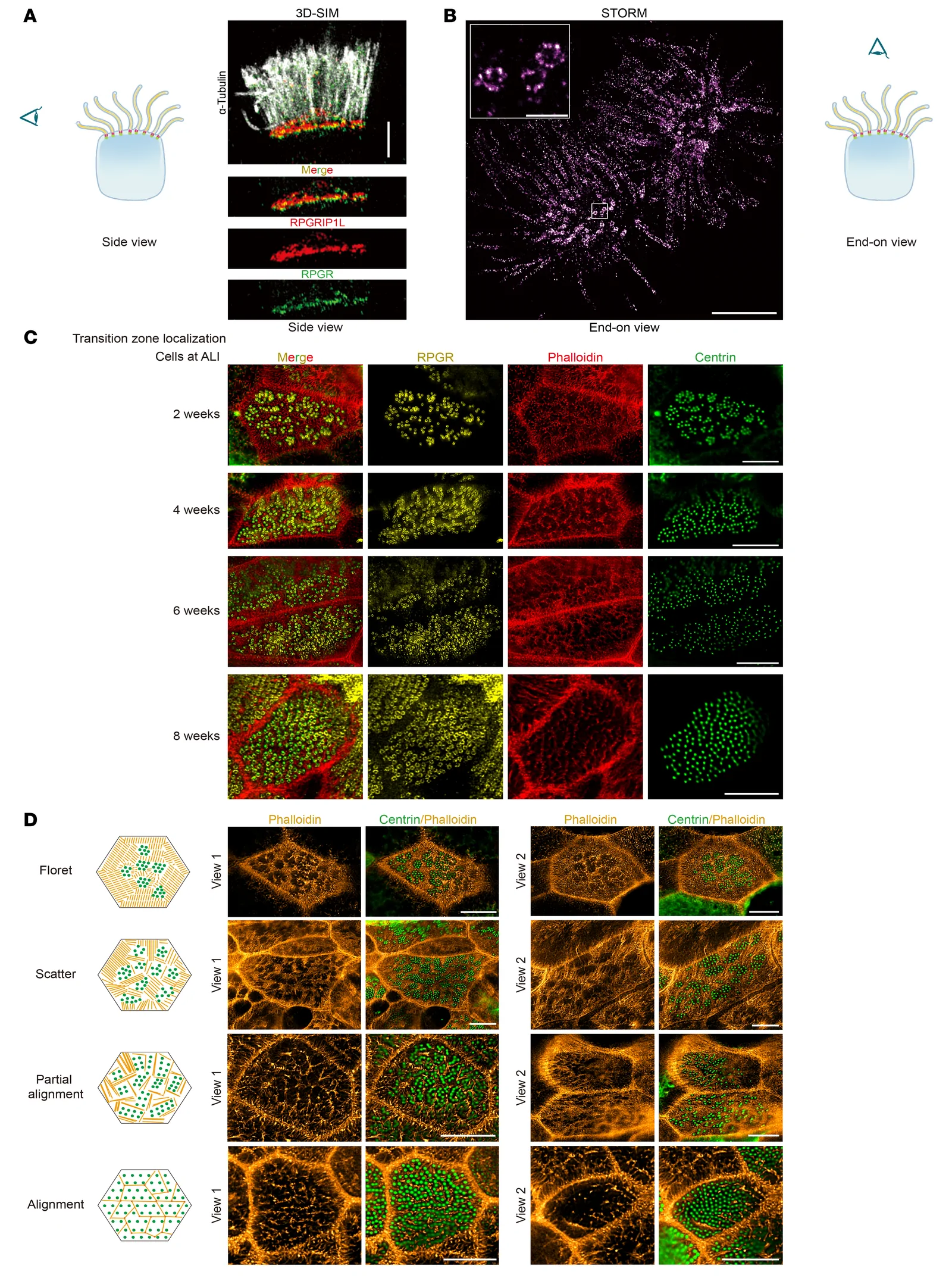
RPGR mainly localized to the TZ and cilia membrane; the relative distribution of RPGR and F-actin is shown. (**A**) 3D-SIM shows RPGR mainly locates to the TZ of motile cilia. Scale bar: 5 μm. (**B**) STORM showed RPGR presents with a dotted ring structure at the TZ (rectangle) and puncta along cilia. Scale bars: 5 μm; insert, 500 nm. (**C**) RPGR locates to the TZ throughout the differentiation of MCCs. Phalloidin labels the apical F-actin, and no colocalization was observed between RPGR and F-actin. Their distributions are largely exclusive. Scale bar: 5 μm. (**D**) The dynamics of apical F-actin during the differentiation of MCCs. Scale bar: 5 μm. Results here represent 4 biological replicates with similar results.

To inspect how RPGR regulates motile cilia, we first examined RPGR subcellular localization by 3D-SIM. We stained airway MCCs from healthy control individuals with a validated RPGR antibody. 3D-SIM provides approximately 120 nm resolution (50) and showed a clear transition zone (TZ) distribution of RPGR by colocalization with the TZ marker RPGRIP1L, as well as a weak signal in cilia (Figure 5A). We also used stochastic optical reconstruction microscopy (STORM) (51), which provides approximately 20 nm resolution, to examine the distribution of RPGR and found that it formed a dotted ring pattern at the TZ for each cilium as well as cilia localized puncta (Figure 5B). Additionally, we examined the distribution of RPGR at different multiciliogenesis stages (2–8 weeks) by stimulated emission depletion (STED) microscopy (approximately 40 nm resolution; ref. 52) and found that RPGR consistently localized to the TZ and motile cilia (Figure 5C and Supplemental Figure 7).

The subcellular localization of RPGR and its interaction with TZ components suggest it might be involved in ciliary transportation and maintenance of cilia composition. Because most cilia of *RPGR* LoF cells were static, we wondered if the outer dynein arms, inner dynein arms, and central pair components were intact. We assessed 13 samples from patients with *RPGR^ex1–19^* or *RPGR^ORF15^* variants and found that outer dynein arm marker DNAH5, inner dynein arm marker DNALI1, and central pair component SPEF2 all showed proper localization (Supplemental Figure 8, A–D), consistent with the normal TEM results (Supplemental Figure 8, E and F) (53). RPGR interacts with the TZ components RPGRIP1L, CEP290, and NPHP4 (54, 55). Immunostaining showed the loss of RPGR did not affect the distribution of these TZ components (Supplemental Figure 9A), and other TZ components, such as AHI1, MKS1, MKS3, and CC2D2A, were all properly localized (Supplemental Figure 9B), suggesting that RPGR is dispensable for the integrity of the TZ.

### RPGR KO MCCs maintained an abnormal, condensed, apical F-actin meshwork.

RPGR has been reported to regulate F-actin polymerization (56, 57), and loss of RPGR in hTERT-RPE1 cells induces the formation of actin bundles (56). Using an *Rpgr* KO mouse model, Megaw et al. found that RPGR regulates photoreceptor ciliary-tip actin dynamics, and its dysregulation affects outer segment membrane turnover (26, 58). To investigate if RPGR also regulates F-actin organization in MCCs, we first looked at the relative distribution of apical F-actin and RPGR. Notably, we found no obvious colocalization between the 2 (Figure 5C). We next stained centrin-GFP–labeled MCCs cultured for different weeks with phalloidin-STAR RED and imaged the dynamics of F-actin using STED microscopy. During the early stage of multiciliogenesis, the apical surface was mostly covered with F-actin meshwork, where the gaps were decorated with centrin-GFP florets (Figure 5D). The F-actin meshwork is critical for basal body docking (59, 60). As MCCs mature, the F-actin meshwork gradually dissolves, allowing more scattered or aligned centrin-GFP–labeled basal bodies to emerge (Figure 5D). The F-actin pattern now condenses into bright puncta or bundles (Figure 5D). The filamentous F-actin is supposed to align basal bodies to synchronize cilia beat (60, 61).

**Figure 6:**
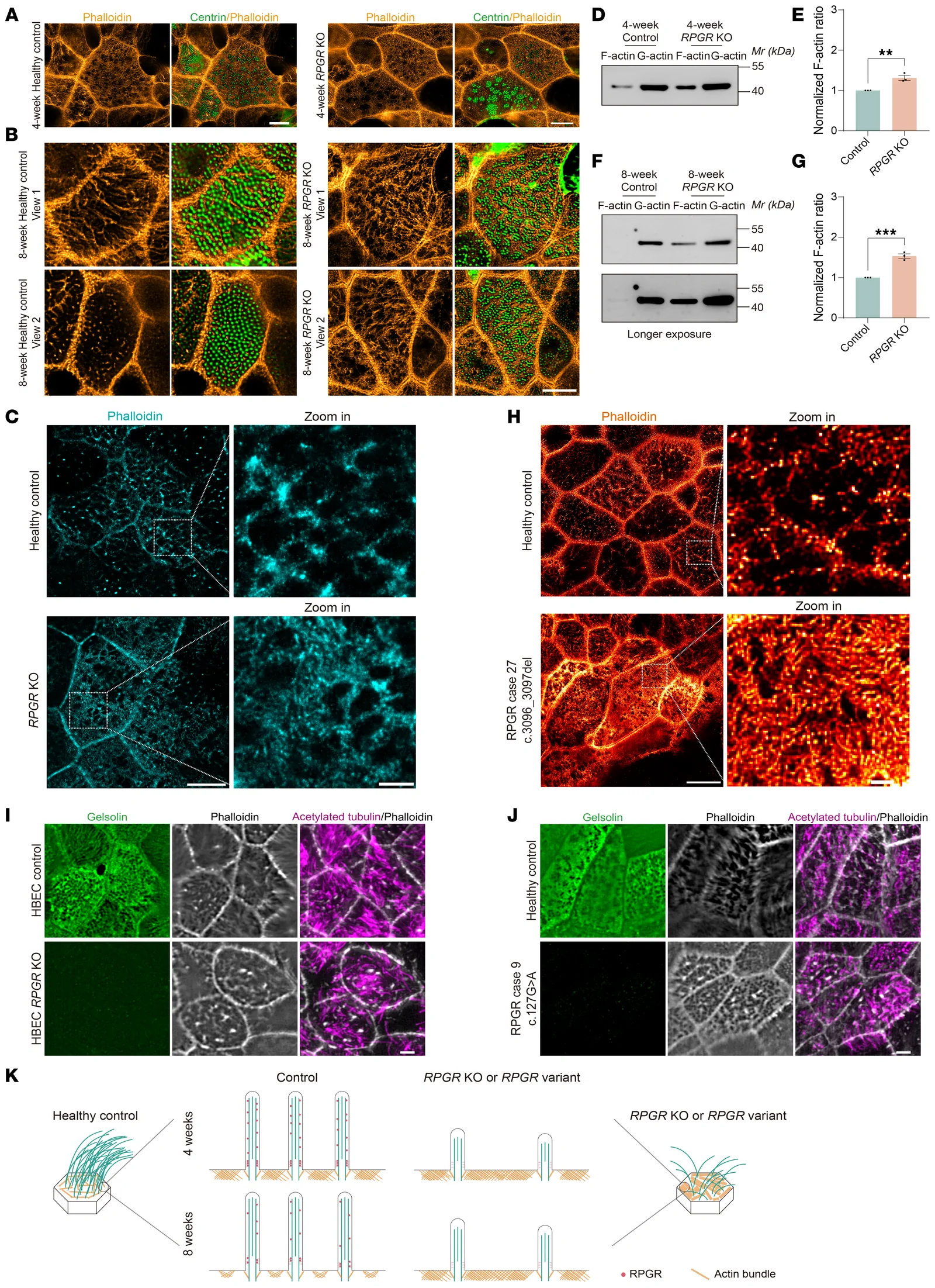
Mature *RPGR* LoF MCCs presented with condensed apical F-actin meshwork that did not dissolve. (**A**) Distribution of F-actin in 4-week control and *RPGR* KO MCCs. Scale bar: 5 μm. (**B**) Distribution of F-actin in 8-week control cells and *RPGR* KO MCCs. Scale bar: 5 μm. (**C**) STORM imaging of the apical F-actin in both *RPGR* KO cells and healthy control cells. Scale bars: 5 μm; insert, 1 μm. (**A**–**C**) Findings from 4 biological replicates are represented. (**D** and **E**) Immunoblotting showed increased F-actin in 4-week *RPGR* KO MCCs. Results were summarized from 3 biological replicates. Mr, relative molecular mass. (**F** and **G**) Immunoblotting showed increased F-actin in 8-week *RPGR* KO MCCs. Results were summarized from 3 biological replicates. (**H**) STED imaging of the apical F-actin in MCCs from patients with RP and healthy control cells. Scale bars: 5 μm; insert, 1 μm. (**I** and **J**) Apical gelsolin was diminished in 4-week *RPGR* LoF MCCs. (**I**) *RPGR* KO HBEC MCCs. (**J**) MCCs from 1 patient with RP. Scale bar: 2 μm. Data represent mean ± SEM. **P < 0.01, ***P < 0.001 by 2-tailed *t* test (**E** and **G**). (**H**) represents results from 8 patients with RP (see Supplemental Figure 11, C and D, for details). (**I**) represents results from 5 biological replicates. (**J**) represents similar results from 17 patients (see Supplemental Figure 13, B and C, for details). (**K**) The proposed model of this study: without *RPGR*, F-actin meshwork persists at the apical surface, preventing ciliation and cilia elongation; cilia beat is severely impaired.

We next characterized the apical F-actin in *RPGR* KO MCCs. In 4-week *RPGR* KO MCCs, F-actin was comparable between control and *RPGR* KO cells (Figure 6A). However, after maturation, *RPGR* KO MCCs preserved the meshwork and failed to form F-actin puncta or bundles (Figure 6B), which affected the extrusion of the cilia. These results were confirmed using STORM imaging of phalloidin-Alexa Fluor 647–labeled MCCs (Figure 6C). We further quantified the F-actin/G-actin ratio in MCCs by immunoblotting, and the result showed an obvious increase in F-actin for both 4-week and 8-week *RPGR* KO samples (Figure 6, D–G), aligning with super-resolution images.

Analysis of MCCs from patients with RP showed results mostly consistent with those observed in *RPGR* KO cells (Supplemental Figure 10, A–D, and Supplemental Figure 11, A–D). For most patients with variants in *RPGR^ex1–19^* (*n* = 6 of 9) or *RPGR^ORF15^* (*n* = 7 of 9), the apical F-actin meshwork in 8-week MCCs could still be readily discerned by either STED or 3D-SIM (Figure 6H; Supplemental Figure 10, C and D; and Supplemental Figure 11, C and D). However, in some patients, the F-actin accumulation seemed to be milder or normal (*n* = 3 of 9 for *RPGR^ex1–19^* variants and *n* = 2 of 9 for *RPGR^ORF15^* variants) (Supplemental Figure 10, C and D, and Supplemental Figure 11E).

To further validate the involvement of F-actin in muticiliogenesis, we treated healthy control MCCs with Rho activator II, a known compound to stabilize F-actin (62, 63), and observed cilia properties at ALI 8 weeks. F-actin stabilization substantially affected cilia length and ciliation, reduced cilia beat frequency, changed cilia beat waveform, and most MCCs lost cilia beat coordination (Supplemental Figure 12, A–I). We also found that bead clearance was paralyzed in Rho activator II–treated MCCs (Supplemental Figure 12J). This suggested that F-actin accumulation could account for the observed motile cilia phenotypes in *RPGR* LoF MCCs.

In photoreceptor and hTERT-RPE1 cells, RPGR regulates actin dynamics through interaction with gelsolin and cofilin (26, 58). Staining gelsolin for *RPGR* KO MCCs showed that in both *RPGR* KO HNCs (*n* = 2) and HBECs (*n* = 3), the cell surface, the layer where F-actin mainly locates, had temporarily diminished the gelsolin signal, suggesting that RPGR might regulate F-actin dynamics through locating gelsolin (Figure 6I and Supplemental Figure 13A). Gelsolin exists in active and inactive forms, and it is the active gelsolin that binds to F-actin (64, 65). Reduction of gelsolin binding to F-actin in *RPGR* KO MCCs suggests that RPGR might be involved in gelsolin activation. We further examined the distribution of gelsolin in MCCs bearing different *RPGR* variants and found a consistent decrease in the gelsolin that colocalized with the apical F-actin (*n* = 8 of 8 for *RPGR^ex1–19^* variants and *n* = 7 of 9 for *RPGR^ORF15^* variants) (Figure 6J and Supplemental Figure 13, B and C).

In summary, RPGR regulates the dynamics of F-actin, and *RPGR* KO MCCs and patient MCCs with pathological *RPGR* variants had an F-actin meshwork that failed to dissolve.

### Motile cilia anomalies caused by RPGR LoF were improved with latrunculin A or Y27632 treatment.

If RPGR regulates motile cilia through F-actin, treatment with actin polymerization inhibitors (64, 66) should rescue or ameliorate the phenotype. Previous studies showed that overexpression of active gelsolin can rescue the ciliation issue in *RPGR* KD hTERT-RPE1 cells (58). We first worked on hTERT-RPE1, due to its quick turnaround time. From our *RPGR* KO hTERT-RPE1 cell pool, we found reduced ciliation and cilia length defects after *RPGR* removal, consistent with findings from previous studies (67) and our motile cilia data (Supplemental Figure 14, A–C). Treatment of hTERT-RPE1 control and *RPGR* KO cells with 0.2 μM latrunculin A (LatA) for 24 hours restored ciliation and cilia length of *RPGR* KO cells (Supplemental Figure 14, D and E). This suggests that LatA can restore the cilia phenotype caused by loss of RPGR and that RPGR functions upstream of actin in cilia regulation. We also applied LatA on the fibroblast cells from 2 patients with RPGR-RP with cilia defects (Supplemental Figure 14, F and H) and found that LatA treatment partially rescued the cilia phenotypes (Supplemental Figure 14, G and I).

To investigate whether LatA treatment could rescue motile cilia, we applied LatA to ALI-cultured *RPGR* KO MCCs. Previous studies indicated an increase of RhoA activity in *RPGR* KD hTERT-RPE1 cells (47) or *Rpgr* KO mouse models (68); therefore, we also used a ROCK inhibitor, Y27632, to modulate actin dynamics. We added LatA or Y27632 starting from the ALI day 0 and observed cilia properties at the ALI week 4, because *RPGR* is strongly expressed during the early stage of multiciliogenesis (Figure 2I and Supplemental Figure 15).

**Figure 7:**
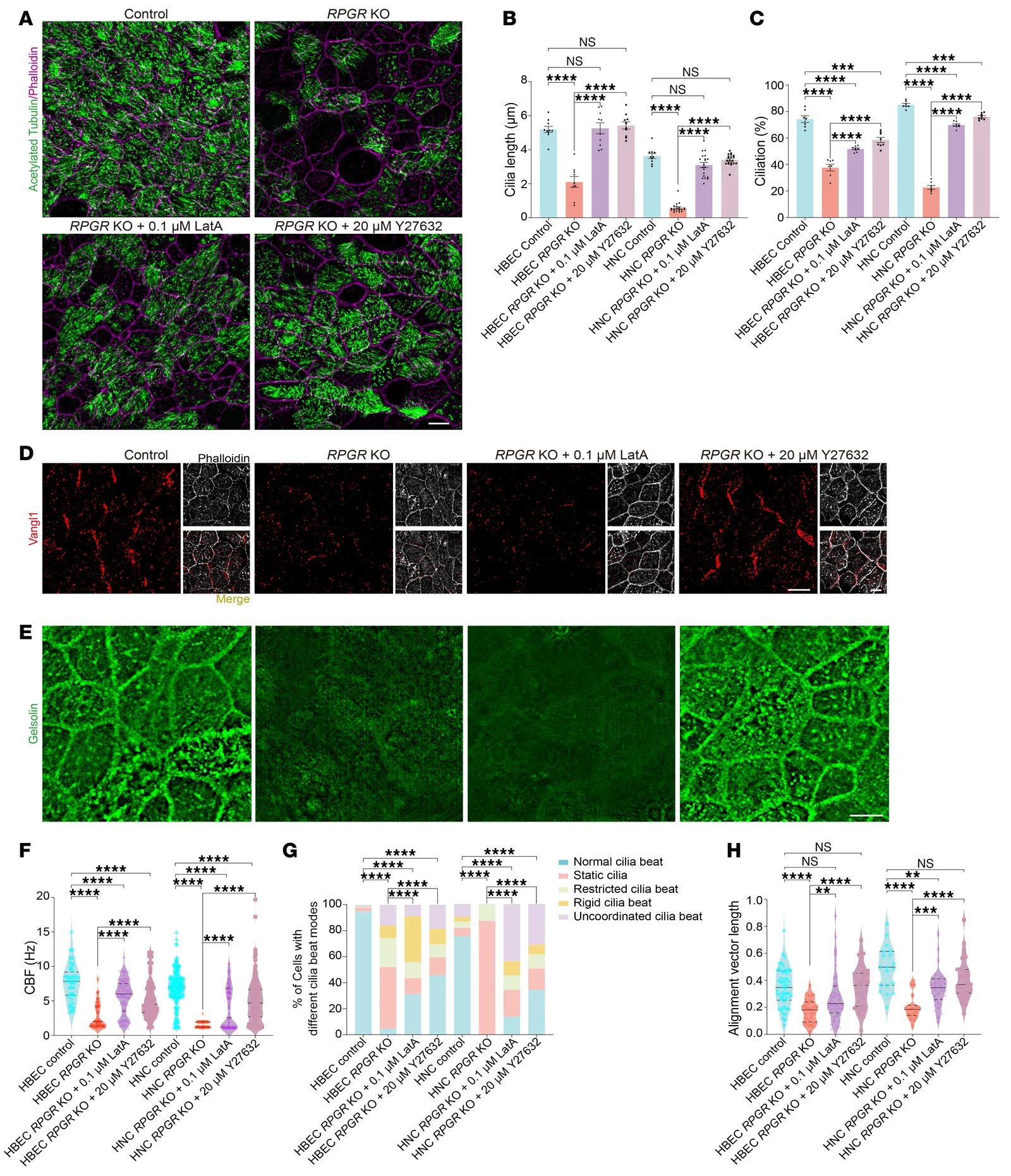
LatA and Y27632 treatment ameliorated the motile cilia defect in *RPGR* KO MCCs. (**A**) Immunostaining showed the reduction in ciliation and cilia length in *RPGR* KO MCCs, along with the rescued phenotypes following LatA and Y27632 treatment. Scale bar: 10 μm. (**B** and **C**) Image quantification shows improvements in cilia length and ciliation for 2 *RPGR* KO biological replicates. (**D**) Y27632, but not LatA, restored the polarized distribution of Vangl1 at the apical surface in the 4-week HBEC sample. Scale bars: 5 μm; insert, 5 μm. (**E**) Y27632, but not LatA, restored the distribution of gelsolin at the apical surface in one 4-week HBEC sample. Scale bar: 5 μm. (**F**–**H**) The cilia beat anomalies in *RPGR* LoF MCCs were partially rescued by LatA or Y27632 treatment. (**F**) Both treatments improved cilia beat frequency (CBF). (**G**) Both treatments partially rescued the waveform, with more cells displaying a normal beating pattern. (**H**) The disrupted cilia beat coordination was partially rescued by LatA or Y27632 treatment. Results were from 2 biological replicates (**B**, **C**, and **F**–**H**). Data represent mean ± SEM. The center, upper, and lower lines represent the median, upper, and lower quartiles, respectively (**F** and **H**). **P < 0.01, ***P < 0.001, ****P < 0.0001 by 2-tailed *t* test (**B**, **C**, **F**, and **H**) or Fisher’s exact test (**G**).

After treatment, although the cilia length could be fully restored, ciliation was partially rescued for both LatA and Y27632 treatment in 2 biological replicates (Figure 7, A–C). Additionally, we examined the distribution of Vangl1 and gelsolin. Although LatA treatment did not rescue their distributions, Y27632 showed obvious rescue of these 2 phenotypes (Figure 7, D and E).

Treatment of *RPGR* KO MCCs with LatA or Y27632 led to significant improvements in cilia beat frequency, waveform, and coordination (Figure 7, F–H, and Supplemental Video 4). We also found that, after treatment with LatA and Y27632, the directional movement of the beads could be readily observed (*n* = 2; Supplemental Figure 16 and Supplemental Video 5), suggesting that F-actin depolymerization can, indeed, ameliorate the motile cilia phenotypes caused by RPGR defect.

**Figure 8:**
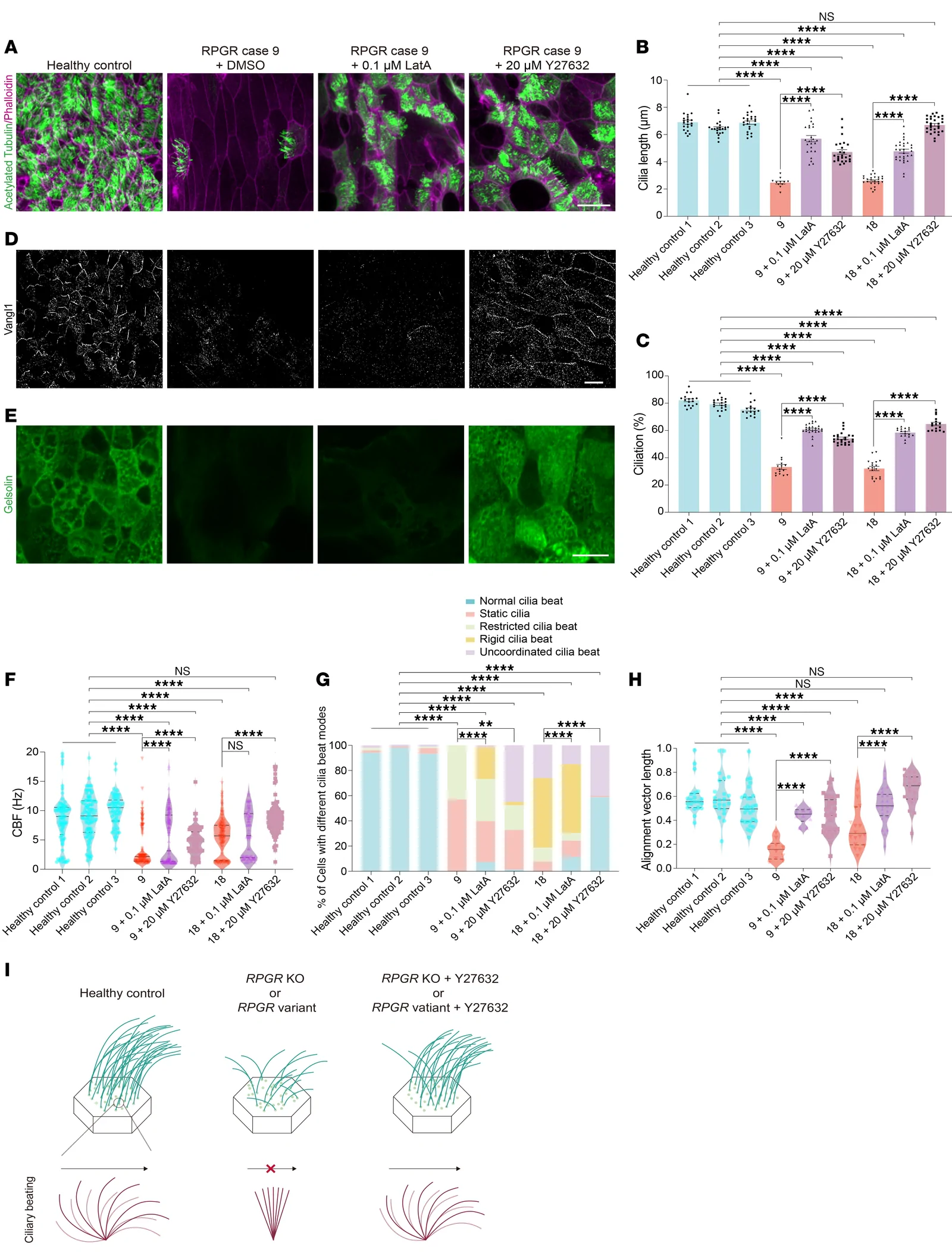
LatA and Y27632 treatment ameliorated the motile cilia defect in patient MCCs with *RPGR* variants. (**A**) Immunostaining showed that LatA and Y27632 can partially rescue cilia length and ciliation defects in the MCCs from a patient with RP (patient 9). Scale bar: 10 μm. (**B** and **C**) The cilia length and ciliation level in MCCs from patients with pathological variants (patients 9 and 18) were improved by LatA and Y27632 treatment. (**D** and **E**) The distribution of Vangl1 and gelsolin in patient cells with *RPGR* defects can be rescued by Y27632 but not LatA. Scale bar: 10 μm. (**F**–**H**) The cilia beat anomalies in MCCs from patients with *RPGR^ex1–19^* or *RPGR^ORF15^* pathological variants were alleviated by either LatA or Y27632 treatment. (**F**) Both treatments improved cilia beat frequency (CBF). (**G**) Both treatments partially rescued the waveform, with more cells displaying a normal beating pattern. (**H**) The disrupted cilia beat coordination was partially rescued by LatA or Y27632 treatment. (**B**, **C**, **F**–**H**) Data represent more than 11 of 17 patients with *RPGR^ex1–19^* or *RPGR^ORF15^* variants. The detailed summary information can be found in Supplemental Figure 17. Data represent mean ± SEM. The center, upper, and lower lines represent the median, upper, and lower quartiles, respectively (**F** and **H**). **P < 0.01, ****P < 0.0001 by 2-tailed *t* test (**B**, **C**, **F**, and **H**) or Fisher’s exact test (**G**). (**I**) A summary cartoon shows that the cilia defects in *RPGR* KO MCCs or *RPGR*-deficient patient MCCs can be alleviated by Y27632 treatments.

We further treated the MCCs from 17 patients with either *RPGR^ex1–19^* or *RPGR^ORF15^* variants with LatA and Y27632 and found results consistent with those we observed in the *RPGR* KO MCCs (Figure 8 and Supplemental Figure 17). Of the MCCs from the 17 patients, treatment with either LatA or Y27632 resulted in the restoration of Vangl1 in cells of 11 patients and restoration of gelsolin in 12 by at least 1 method. Improvements in cilia length, beat frequency, waveform, and coordination were observed in MCCs of 12 patients, and MCCs of 13 patients had an increase in ciliation levels. This led to enhanced mucociliary clearance for 12 cases. Notably, no rescue effect was observed in 4 patients. No obvious difference was found between *RPGR^ex1–19^* and *RPGR^ORF15^* variants.

Interestingly, Y27632 outperformed LatA in rescuing the distribution of Vangl1 and gelsolin and in improving the cilia beat waveform (Figures 7 and 8 and Supplemental Figure 17). This result suggests that RPGR may not directly interact with actin but rather regulates actin dynamics by suppressing the RhoA/ROCK activity.

Moreover, we tested treatment starting at a later stage (from ALI week 4 and observed cilia properties at ALI week 8) and also found improvements in bead clearance in 2 of 2 *RPGR* KO MCCs and 5 of 9 patient MCCs (Supplemental Figures 18 and 19). Our study suggests that early rescue has a better performance, which is consistent with the increased expression of RPGR during the early stage of multiciliogenesis. Additionally, after 1 month of treatment withdrawal, there was a slight deterioration of cilia properties in *RPGR* KO or patient MCCs; however, most parameters were still improved compared with the cells without any treatment, and mucociliary clearance was still preserved after drug withdrawal (*n* = 8 of 9 patient MCCs and *n* = 2 of 2 *RPGR* KO MCCs) (Supplemental Figures 20 and 21).

In summary, the structural anomalies and dysfunctions of motile cilia caused by *RPGR* LoF can be partially rescued by either LatA or Y27632 treatment.

## Discussion

In this study, we investigated how loss of RPGR affects motile cilia function and its role in respiratory diseases. By examining, via advanced fluorescence microscopy, the nasal cilia from patients with *RPGR*-RP with a wide range of *RPGR* variants and those of *RPGR* KO MCCs, we reveal that RPGR regulates ciliation, cilia length, and cilia beat properties in airway MCCs. We further suggest these changes may result from a persistent F-actin meshwork at the apical surface because these phenotypes were improved with treatment with the G-actin sequestor LatA or the ROCK inhibitor Y27632 (Figures 7 and 8). This is, to our knowledge, the first mechanistic study of RPGR’s role in cilia motility. We show that all variants studied led to motile cilia anomalies irrespective of the isoform. This reveals a new mechanism for *RPGR*-related respiratory diseases, such as PCD, which shares similarities with recent studies of RPGR in the sensory cilia of photoreceptors and hTERT-RPE1 cells (26, 56, 58, 69).

Variants in *RPGR* lead to a range of motile cilia abnormalities, which are associated with a range of airway abnormalities, the severe end of which is PCD. PCD remains an underdiagnosed disorder of motile cilia, for which improper management can substantially affect airway health outcomes (70, 71). A delayed diagnosis can lead to lung deterioration, even to lung transplantation or death (8). Patients with *RPGR* variants are usually referred to an eye care provider because they have vision loss, while their respiratory symptoms can be mild and often overlooked. The diagnosis of *RPGR*-related PCD can be challenging and certainly underrecognized, because the cilia phenotype can be different from other PCD causes. PCD caused by variants in other genes may be diagnosed by demonstrating classic cilia ultrastructural defects by TEM, such as missing outer and/or inner dynein arms. However, *RPGR* variants do not result in a classic ciliary ultrastructural phenotype (31). Therefore, TEM is not useful for diagnosing *RPGR*-PCD. Four previously undiagnosed individuals with PCD were identified through this study and are now treated appropriately in PCD clinics. Furthermore, we show that cilia length, ciliation, motility, and coordination were abnormal even in patients with mild or normal respiratory phenotypes.

Importantly, this study suggests that both *RPGR^ex1–19^* and *RPGR^ORF15^* variants can contribute to impaired mucociliary clearance and airway dysfunction. The variants affecting both isoforms appear to be associated with oto-sino-pulmonary disease. Furthermore, our study demonstrated variability in clinical outcomes, even in patients carrying the same variant. This could be explained by different environmental factors, genetic backgrounds, and genetic modifying influences. Unlike earlier studies suggesting that *RPGR^ex1–19^* was the sole isoform in airway MCCs, we show that the *RPGR^ORF15^* isoform is expressed in MCCs and that variants in *RPGR^ORF15^* could affect cilia properties, potentially explaining the high frequency of respiratory signs and symptoms observed in some *RPGR^ORF15^* variant carriers.

We found that RPGR in MCCs localizes both to TZ and cilia. Because the C-terminal of the canonical RPGR is prenylated (57), RPGR should be distributed along the cilia membrane, which is also different from most PCD-causative genes. Consistent with this observation, we did not identify changes in DNAH5, DNALI1, and SPEF2 distributions. We did not find localization changes for either RPGR interactors or TZ components.

Our work revealed that F-actin dynamics play a central role in the regulation of motile cilia by RPGR (Figure 6K and Figure 8I). In control cells, F-actin first forms an apical meshwork, which is presumably important for basal body docking and ciliation. The apical F-actin meshwork later disassembles and forms F-actin patches or bundles, aligning basal bodies for cilia stabilization and beat synchronization. Unlike control cells, mature *RPGR* LoF MCCs retain the condensed meshwork, which restricts ciliation and cilia elongation, and limits cilia beat. This phenotype correlates with the reduced level of active gelsolin observed at the apical surface, which is most obvious for 4-week MCCs. Treatment with either LatA or Y27632 partially rescued cilia length and ciliation, and improved the cilia beat defect, leading to better bead clearance. We found that Y27632 had a relatively better performance than LatA. Y27632, unlike LatA, can restore the distribution of gelsolin and Vangl1. It is possible that RPGR does not directly interact with actin but rather influences actin through the RhoA/ROCK pathway. This result aligns with previous literature indicating upregulation of RhoA activity in *Rpgr* KO mouse retina (68). Because RPGR works upstream, RhoA/ROCK signaling pathway regulators could counteract the loss of RPGR, which may explain the heterogeneous phenotypes. Individual differences in RhoA/ROCK signaling pathway regulators could also partially explain the phenotype heterogeneity observed between different patient samples. Moreover, there are FDA-approved drugs that target ROCK1/ROCK2, and using ROCK inhibitors to modulate F-actin dynamics in patients with *RPGR*-PCD might be a promising therapeutic strategy.

In photoreceptor-connecting cilia, RPGR regulates membrane turnover through actin dynamics, which is key to the stability of this transport system (26, 58). In addition, we show that RPGR also relies on actin to regulate motile cilia in MCCs. Our study reveals that RPGR uses the same pathway to achieve different outcomes across various cell and cilia types.

Disruption of rotational, but not planar, polarity is a common feature of PCD and has been proposed to aid PCD diagnosis (41, 72, 73). Our study showed that loss of RPGR affects both planar and rotational polarity, another distinction from other PCD-associated genes. RPGR has been reported to be associated with the PCP pathway by affecting Dvl through the proteasome in hTERT-RPE1 cells (47). To our knowledge, this is the first study showing the consequent loss of Vangl1 at cell junctions. Because the downstream of the PCP pathway involves actin dynamics, we speculate that the PCP pathway might also be involved in the regulation of actin by RPGR.

Our study has several limitations. Our sample size was limited due to the rareness of *RPGR*-RP and recruitment during the COVID-19 pandemic. Heterogeneous *RPGR* KO cell pools were generated rather than a homogenous cell line, which could contribute to the heterogeneity of the cilia beat in *RPGR* KO MCCs. Our study was also limited to human cell ex vivo models, and further mechanistic insights might be gained from using in vivo mouse models for investigation, although the mouse respiratory phenotype is usually mild (72, 74–76). Although we identified a role of F-actin turnover in the pathogenesis of *RPGR*-PCD, we cannot exclude the role of other interacting pathways in the alteration of motile cilia phenotypes. Further mechanistic insights may be gained by assessing motile cilia protein composition changes using proteomics or Cryo-EM (77–79). Finally, because our study was cross-sectional, we lack information on the progression of oto-sino-pulmonary disease in these patients.

In summary, our study shows, for the first time, to our knowledge, that *RPGR^ORF15^* is expressed in airway MCCs, and variants in both isoforms could lead to motile cilia defects in symptomatic and asymptomatic patients. *RPGR^ex1–19^* variants led to a more severe airway and cilia phenotype, but only a few patients manifested severe enough symptoms to be classified with a clinical diagnosis of PCD. This study reveals that the actin pathway is involved, which could be manipulated to treat *RPGR*-PCD. Our study opens new avenues of research for *RPGR*-related diseases and opportunities for improved PCD diagnosis and patient treatment.

## Methods

### Sex as a biological variable.

Our study examined human airway cells donated by male and female volunteers, with similar findings observed for both sexes. Because this genetic disease is X-linked, most patient cells were from male individuals.

### Study design.

The purpose of this project is to study the mechanism of PCD caused by *RPGR* defects. Thirty-two patients with RP with a range of respiratory symptoms were recruited from ophthalmology clinics. Patients completed study procedures at The Hospital for Sick Children (SickKids) in Toronto, Ontario, Canada, and the BC Children’s Hospital (BCCH) in Vancouver, British Columbia, Canada. *RPGR* KO MCCs were also generated to validate the cellular phenotypes observed in patient cells. Super-resolution microscopy, live-cilia beat imaging, and drug rescue experiments were carried out to characterize the cellular phenotypes and to reveal the mechanism.

### Patient phenotype characterization.

All patient participants had a comprehensive eye exam in addition to optical coherence tomography imaging, Goldmann kinetic visual field test, and electroretinography when possible (Supplemental Table 2). The respiratory phenotypes were characterized using PCD-specific protocols (i.e., questionnaire, natural history, nasal nitric oxide measurements, chest x-ray, spirometry, and physical exam) (Supplemental Table 2)

### Antibodies and labeling reagents.

The primary antibodies used for immunofluorescence were as follows: rabbit anti-RPGR (1:200; Atlas Antibodies, HPA001593); mouse anti–acetylated tubulin (1:3,000; Sigma, T7451); mouse anti–α-tubulin (1:3,000; Sigma, T9026); rabbit anti-POC1B (1:200; Thermo Fisher Scientific, PA524495); mouse anti-centriolin (1:50; Santa Cruz, sc-365521); rabbit anti-DNAH5 (1:200; Atlas Antibodies, HPA037470); rabbit anti-DNALI1 (1:200; Atlas Antibodies, HPA028305); rabbit anti-SPEF2 (1:200; Atlas Antibodies, HPA039606); rabbit anti-RPGRIP1L (1:50; ProteinTech, 556160-1-AP); rabbit anti-MKS1 (1:50; ProteinTech, 16206-1-AP); rabbit anti-MKS3 (1:50; ProteinTech, 13975-1-AP); rabbit anti-CEP290 (1:100; ProteinTech, 22490-1-AP); rabbit anti-NPHP4 (1:100; ProteinTech, 13812-1-AP); rabbit anti-AHI1 (1:50; Atlas Antibodies, HPA031698); rabbit anti-CC2D2A (1:50; Atlas Antibodies, 22293-1-AP); rabbit anti-Vangl1 (1:50; Atlas Antibodies, HPA025235); rabbit anti-gelsolin (1:50; ProteinTech, 11644-2-AP); and rabbit-ARL13B (1:200; ProteinTech, 17711-1-AP). The secondary antibodies used for immunofluorescence were as follows: goat anti–rabbit IgG (H+L) 2nd Antibody, Alexa Fluor 488 (1:200; Thermo Fisher Scientific, A11034); goat anti–rabbit IgG (H+L) 2nd Antibody, Alexa Fluor 555 (1:200; Thermo Fisher Scientific, A21428); F(ab′)2-goat anti–mouse IgG (H+L) Cross-Adsorbed Secondary Antibody, Alexa Fluor 647 (1:200; Thermo Fisher Scientific, A21237); F(ab′)2-goat anti–rabbit IgG (H+L) Cross-Adsorbed Secondary Antibody; and Alexa Fluor 647 (1:200; Thermo Fisher Scientific, A21246). The reagents for labeling F-actin were as follows: Alexa Fluor 647 Phalloidin (1:50; Thermo Fisher Scientific, A22287) and Phalloidin STAR RED (1:100; Abberior, STRED-0100-20UG).

### Cell collection and culturing.

Using cytology brushes as previously described (41, 80), human nasal cells were obtained from 12 healthy volunteers at The Hong Kong University of Science and Technology (HKUST) and SickKids and from patients with *RPGR* variants at SickKids and BCCH. For all the measurements, the researchers were blind to the genetic information of the participants. After nasal brushing, HNCs were collected in BEBM bronchial epithelial cell growth medium (Lonza, cc-3170), washed, and subcultured until confluence. Cells were subsequently seeded on 24-Transwell inserts and differentiated with PneumaCult-ALI medium (STEMCELL Technologies, 05001) following the manufacturer’s protocol.

HBECs were sourced from Lonza (catalog cc2504S) and expanded and differentiated following the manufacturer’s protocol. The BEBM and PneumaCult-ALI media were supplemented with vancomycin, tobramycin, gentamicin, and antibiotic-antimycotic.

HEK293T cells (a gift from Ting Xie, HKUST) were cultured with high-glucose DMEM (Thermo Fisher Scientific, 11965084) supplemented with 10% FBS and 1% streptomycin/penicillin.

hTERT-RPE1 cells (ATCC, CRL-4000) were cultured with DMEM/F12 medium (Thermo Fisher Scientific, 11320033), supplemented with 10% FBS, 1% streptomycin/penicillin, and 0.1 mg/mL hygromycin. FBS was removed from the medium to induce ciliation of hTERT-RPE1.

Fibroblast cell lines were established at the Biobank at SickKids and those from patients with RP were cultured in MEM-α (Thermo Fisher Scientific, 12561056) with 10% FBS. The growth medium was changed with Opti-MEM (Thermo Fisher Scientific, 31985070) to induce the ciliation of skin fibroblasts.

### Statistics.

All experiments were conducted at least twice, and statistical analyses (2-tailed *t* test, 2-way repeated ANOVA followed by Šídák’s post hoc test, or Fisher’s exact test) were performed with GraphPad Prism 10. A *P* value less than 0.05 was considered statistically significant. The details of the statistics in this study are specified in the main text, figures, and/or figure legends.

### Study approval.

The study was approved by the following research ethics board protocols: HREP-2021-0268 (HKUST), 1000005895 (SickKids), and H20-03566 (BCCH). Written informed consent has been given by all participants or their guardians.

### Data availability.

The underlying data related to the figures can be found in the Supporting Data Values XLS file. The single-cell sequencing data are available from the Sequence Read Archive (access no. PRJNA548516). The code for analyzing cilia beat frequency and rotational polarity has been uploaded to Github (https://github.com/liuzhorizon/RPGR, commit ID fa4d962; and https://github.com/liuzhorizon/PCDDiagnosis_Quantitative_SuperResolution, commit ID 2e3d197).

The detailed methods can be found in the Supplemental Methods.

## Author contributions

YW and ZL designed the experiments, and YW performed most of the experiments. SDD, WBW, ET, KAJS, LHH, JML, and EH evaluated the clinical phenotypes, provided the patient samples, and collected the confocal images. BL developed methods for analyzing the cilia beat data. JC generated the *RPGR* KO hTERT-RPE1 cells. PYW contributed to immunolabeling and data analysis. JZ and MH contributed to single-cell RNA-seq data analysis. NPC contributed experiments related to F-actin. VM and HCF participated in the early studies of the project. YW and ZL wrote the manuscript. ZL, SDD, EH, and ET revised the manuscript.

## Conflict of interest

The authors have declared that no conflict of interest exists.

## Funding support

- General Research Fund from the Research Grants Council of Hong Kong (grants 16100823, 26101022, and 16101324 to ZL).
- Canadian Institutes of Health Research project (grants FRN 156154 SDD, VM, and EH).
- The Henry Brent Chair in Innovative Pediatric Ophthalmology Research to EH.

## Supplementary Material

Supplemental data

Unedited blot and gel images

Supplemental table 1

Supplemental table 2

Supplemental table 3

Supplemental table 4

Supplemental video 1

Supplemental video 2

Supplemental video 3

Supplemental video 4

Supplemental video 5

Supporting data values

## Version 1 03/31/2026

In-Press Preview

## Version 2 05/15/2026

Electronic publication

## Version 3 09/21/2026

Updated Acknowledgments section
