# Supplementary material for: Loss of RPGR disrupts motile cilia and causes primary ciliary dyskinesia by affecting F-actin dynamics
Source: J Clin Invest. 2026 Mar 31;136(10):e193367. doi: 10.1172/JCI193367 (PMC13178666; doi:10.1172/JCI193367)
Supplement: Supplemental data [file jci-136-193367-s107.pdf]

Loss of RPGR disrupts motile cilia and causes primary ciliary dyskinesia by affecting F-actin dynamics

Authors: Yang Wu[1], Erika Tavares[2], Binrun Liang[1], Wallace Wee[3], Vito Mennella[4], Han-Chao Feng[1], Jiaying Cao[1], Pui Yee Wong[1], Jiayi Zheng[5], Mu He[5], Kirk Stephenson[6], Liran Hanan[6], Janice Min Li[2], Nan-Peng Chen[7], Sharon D Dell[3, 8 #], Elise Heon[2,6 #], Zhen Liu[1 #]

Affiliations:

[1]Department of Life Science, The Hong Kong University of Science and Technology, Hong Kong SAR, China.

[2]Genetics & Genome Biology Program, The Hospital for Sick Children, Toronto, Canada.

[3]Child Health Evaluative Sciences, The Hospital for Sick Children, Toronto, Canada.

[4]Department of Biochemistry, School of Biological and Behavioural Sciences, Queen Mary University of London, London, United Kingdom.

[5]School of Biomedical Sciences, The University of Hong Kong, Hong Kong SAR, China.

[6]Department of Ophthalmology and Vision Sciences, The Hospital for Sick Children, University of Toronto, Toronto, Canada.

[7]Institute of Systems and Physical Biology, Shenzhen Bay Laboratory, Shenzhen, China.

[8]Division of Respiratory Medicine, BC Children's Hospital, The University of British Columbia, Vancouver, Canada.

# To whom correspondence should be addressed:

Sharon D Dell, 4480 Oak Street, Vancouver, BC, V6H3V4, Canada, +16048752119, sharon.dell@bcchr.ca; Elise Heon, 170 Elizabeth St, Toronto, ON M5G 1E8, Canada, +14168136525, elise.heon@sickkids.ca; or Zhen Liu, 1 University Road, Clear Water Bay, Kowloon, Hong Kong SAR, China, +85234692553, zhenliu@ust.hk;

List of Supplemental Materials

Supplemental methods

Supplemental figures

Supplemental Figure 1. Validation of the predicted variants through Sanger sequencing.

Supplemental Figure 2. MCCs with *RPGR* defects presented with reduced cilia length, and disrupted planar polarity.

Supplemental Figure 3. Workflow of single-cell cilia beat frequency analysis.

Supplemental Figure 4. Comparison of motile cilia properties across different patient groups.

Supplemental Figure 5. Validation of the expression of $RPGR^{ORF15}$ isoform in human airway MCCs.

Supplemental Figure 6. Characterization of $RPGR^{ORF15}$ KO MCCs generated with an ORF15-specific gRNA.

Supplemental Figure 7. RPGR is also located in motile cilia throughout the differentiation of MCCs.

Supplemental Figure 8. RPGR defect didn't affect the distribution of ODA, IDA, or CP components.

Supplemental Figure 9. The subcellular localization of RPGR interactors and transition zone components was largely unaffected in *RPGR* KO MCCs.

Supplemental Figure 10. Apical F-actin meshwork accumulated in the MCCs from RP patients with pathological variants in *RPGR*.

Supplemental Figure 11. STED revealed that apical F-actin meshwork accumulated in the MCCs from RP patients with pathological variants in *RPGR*.

Supplemental Figure 12. Stabilizing F-actin in healthy control MCCs affected multiciliogenesis and ciliary motility.

Supplemental Figure 13. *RPGR* defect led to diminished apical gelsolin.

Supplemental Figure 14. Cilia issues in *RPGR* KO hTERT-RPE1 were rescued with LatA treatment.

Supplemental Figure 15. RPGR expression at different developmental stages of MCCs.

Supplemental Figure 16. LatA and Y27632 treatment ameliorated the mucociliary clearance defect caused by *RPGR* loss of function.

Supplemental Figure 17. LatA and Y27632 treatment partially rescued the MCCs from patients with pathological variants in *RPGR*.

Supplemental Figure 18. LatA and Y27632 treatment starting from ALI 4 weeks also

ameliorated the motile cilia defect caused by *RPGR* KO.

Supplemental Figure 19. LatA and Y27632 treatment starting from ALI 4 weeks also ameliorated the motile cilia defect in patient MCCs.

Supplemental Figure 20. Withdrawal of LatA and Y27632 treatment led to a decline in cilia properties but mucociliary clearance was preserved.

Supplemental Figure 21. Examining patient MCCs showed that withdrawal of LatA and Y27632 led to a decline in cilia properties, but mucociliary clearance was preserved.

Supplemental tables

Supplemental Table 1.

(1) List of patients involved in this study with the disease-associated $RPGR^{ex1\text{-}19}$ and $RPGR^{ORF15}$ variants.

(2) Disease-associated variants in the patient cohort.

Legend and abbreviations: gDNA: the genomic DNA position of the variant; ClinVar: database of reported variants with associated phenotypes; ACMG: the American College of Medical Genetics variant classification system; GnomAD: control database; Revel, SIFT, MutScore, PhiloP, Splice AI, and Pangolin: publicly available software used to determine the pathogenicity of variants.

Supplemental Table 2.

(1) Summary of the airway assessments.

Legend and abbreviations: Age is in years, NNO: nasal nitric oxide measurements, CXR: chest X-ray, CT: computed tomography.

(2) Summary of the eye phenotypes.

Legend and abbreviations: Age in years at the time of test; BCVA: best corrected visual acuity; OD: right eye, OS: left eye, refraction is in diopters; ERG: electroretinogram; RCD: rod-cone dystrophy, when the cases are advanced it is hard to determine; GVF: Goldmann visual field width in degrees (normal width is 120 degrees); HRR: color vision test, result out of 6 plates; CRT: central retinal thickness measured in µm.

Supplemental Table 3. Summary of all the patients' cilia characteristics in this study.

Supplemental Table 4. Oligonucleotides and plasmids used in this study.

Supplemental videos

Supplemental Video 1. Cilia beat video for MCCs from healthy control, case 9, and case 28.

Supplemental Video 2. Cilia beat video for control and *RPGR* KO MCCs (3 biological replicates).

Supplemental Video 3. Defective mucociliary clearance in *RPGR* KO MCCs revealed by the

beads propelling experiment.

Supplemental Video 4. Cilia beat video for control and *RPGR* KO MCCs treated with LatA and Y27632.

Supplemental Video 5. Beads propulsion video for control and *RPGR* KO MCCs treated with LatA and Y27632.

Supplemental materials

Supplemental methods

Plasmid construction and lentivirus preparation

The lentiCRISPRv2 (Addgene, #52961) plasmid was digested with BsmBI-v2 (NEB, R0739), and ligated with annealed oligos. The guide sequences for *RPGR* KO are as follows (1): Guide 1: GTCCCTGTACATCTTTCATG and Guide 2: AAAGTGAAATTAGCTGCCTG. The guide sequence targeting the ORF15 region is: GTAGTTCAGGAGCAGAACAC. For lentivirus packaging, HEK293T cells were cultured with high-glucose, sodium pyruvate-supplemented DMEM media (ThermoFisher Scientific, 11995073) with 10% fetal bovine serum, and transfected with pMD2.G (Addgene, #12259), psPAX2 (Addgene, #12260), and the lentiviral gRNA plasmid with the 1:10:10 mass ratio by applying PEI MAX (Polysciences, 24765) in Opti-MEM. The medium was changed the next day, and the viral supernatant was harvested 48h post-transfection, filtered with 0.45 μm filter, concentrated with PEG-it virus precipitation solution (ExCell Bio, LV810A-1), and frozen in a –80°C freezer for later use.

Generation of CRISPR KO cells

We generated *RPGR* KO pools in hTERT-RPE1 cells using lentivirus transduction at an MOI of 2 (each cell receives a copy of lentivirus expressing gRNA1 and a copy of lentivirus expressing gRNA2). The resultant KO pool was selected with puromycin treatment for 7 days. The whole genome DNA was extracted, and the KO region was amplified using designed primers (Forward: ACAAGGGGTTTGTATGGATAAA and Reverse: AAAACCCTAATTTACTGTTGCC). The ORF15 KO region was amplified using the following primers: GGAAGGTGCAAGTGAGAGTCA and CTGAGGCCCAATGAGTACCT. The KO efficiency was evaluated by DNA gel electrophoresis and by Sanger sequencing, which was further analyzed by the ICE CRISPR analysis tool (SYNTHEGO).

HNC/HBEC *RPGR* KO pools were generated using lentivirus transduction (2–4). Low passage basal cells (P01-P02) were cultured on collagen (STEMCELL Technologies, 07001) coated 6-well plate in BEBM medium, and incubated until cells reached ~50% confluence. For lentivirus transduction, the BEBM medium was supplemented with 15 μg/mL polybrene (Santa Cruz, sc-134220) and 20 μmol/mL HEPES (ThermoFisher Scientific, 15630080), and the lentivirus transduction was conducted at an MOI of 2. When basal cells reached ~80% confluent, they were sub-cultured with feeder cells in DMEM medium, supplemented with 7% fetal bovine serum, 30% Ham's F-12 nutrient mix (ThermoFisher Scientific, 11765047), 24 mg/L adenine (Sigma, 1152), 4.5 mg/L hTGF (ThermoFisher Scientific, AF-100-15), 1 μg/L hydrocortisone (StemCell, 7925), 10 mg/L insulin (Sigma, I9278), and 8.6 μg/L cholera toxin (Sigma, C8052). The feeder cells for co-culturing were generated by irradiation of puromycin-resistant NIH-3T3 cells (gift from Shuhuai Yao, HKUST, Hong Kong SAR) (3000-5000 rads of gamma radiation). The resultant KO pool was selected in 1 μg/mL puromycin for 7-10 days until basal cells reached confluency, and seeded on 24-transwell inserts with a density of ~5.0 x 10^5 cells/cm^2. When cells reached confluency, the medium was changed to PneumaCult-ALI for differentiation. The genomic DNA was extracted from a portion of the genetically edited basal cells for KO efficiency evaluation.

Immunofluorescence

Samples were fixed with 4% paraformaldehyde (Sigma, 158127) and 0.1% glutaraldehyde (Electron Microscopy Sciences, 16019) for 15 minutes, then reduced with 0.1% sodium borohydride (Sigma, 2643) for 6-7 minutes, and blocked with 3% bovine serum albumin (Sigma, A8806) and 0.2% Triton-X 100 in PBS (pH 7.4) for 30 minutes. For methanol fixation, samples were fixed with –20°C histological grade anhydrous methanol for 30 minutes and then blocked with 3% bovine serum albumin and 0.05% Tween 20 in PBS (pH 7.4) for 30 minutes. Optionally, to preserve the morphology of motile cilia, the Transwell filter samples were treated with 0.01% Triton-X 100 in PBS (pH 7.4) for 1 minute before methanol fixation. Primary antibodies were incubated at 37°C for 3 hours or at 4°C overnight. Secondary antibodies were incubated at 37°C for 30 minutes. DAPI (1 μg/mL) was incubated at 37°C for 1 hour, and Phalloidin-Alexa 647 (1:50 dilution) was incubated at 37°C for 7 hours. All washing steps were processed with PBS (pH 7.4). Samples were mounted with 0.5% n-propyl gallate and 80% glycerol in PBS (pH 7.4), sandwiched between coverslips (25 mm, No. 1.5 H, Deckglaser) and slides, and sealed with nail polish.

Live cell cilia beat observation

Transwell cultured airway epithelial cells were gently rinsed with PBS to remove mucus and cell debris. The filters were then cut off, and incubated with PneumaCult-Ex media containing 100 μg/mL wheat germ agglutinin (WGA)-Alexa 488 (ThermoFisher Scientific, W11261) at 37°C for 20 minutes. The medium was then changed to PneumaCult-Ex media. The labeled filter was put into a confocal dish (MatTek, P35G-1.5-14-C) with the sample side facing the coverslip for imaging. WGA-488 labeled samples were captured at around room temperature (23°C) under an inverted microscope, and a 488nm excitation laser was used to excite the fluorophores, and the cilia beat data were collected using a 100x/1.50 oil immersion objective lens. Videos were recorded at 50 frames per second (fps) for 10 seconds.

For the beads propelling experiment, Transwell cultured airway epithelial cells were gently rinsed with PBS to remove mucus, and the whole filter was put onto a confocal dish with the sample side facing against the coverslip. Fluorescent beads with a diameter of 1 μm (ThermoFisher Scientific, F8821) were diluted 1:50 with PneumaCult-Ex media, and the diluted beads were added to the apical surface. Videos were captured at around room temperature (23°C) under an inverted microscope, and a 561nm excitation laser was applied to excite the fluorescent beads, and the data were collected using a 60x/1.20 water immersion objective lens. Videos were recorded at 50 fps for 10 seconds.

SIM imaging and data analysis

3D-SIM datasets were collected using a Zeiss Elyra 7 Lattice SIM with an alpha Plan-Apochromat 63x/1.4 Oil immersion objective lens and with an additional 1.6x optovar. The fluorophores were excited with 488 nm (500 mW), 561 nm (500 mW), and/or 647 nm lasers (500 mW) tuned to 0.5-10% power through the ZEN software. For each image field, grid excitation patterns were applied on the sample plane and collected for thirteen phases. The fluorescence was collected by the objective and filtered by proper band-pass mirrors before reaching the camera. Two PCO edge sCMOS cameras were used to acquire a 5-10 μm thick Z

stack with 100 nm per slice. The raw data were reconstructed using the SIM module of ZEN Software. Channel alignment was conducted using a calibrated file generated by TetraSpeck beads (ThermoFisher Scientific, T7279). Maximum-intensity projection images were produced for subsequent analysis.

Cilia length measurement

Side-view multiciliated cells on the maximum intensity projection images were chosen for measurements. The cilia length was obtained by measuring the distance from the cilia tip to the base using the free drawer tool of Zeiss ZEN. The average length of 10 cilia within one cell was defined as the cilia length of each MCC.

Ciliation level

The ALI filter samples were fixed with methanol and stained as described above with antibodies against acetylated tubulin and POC1B. For each cell, the number of cilia and basal bodies was manually counted using the software Fiji, and the ratio of the total number of cilia to the total number of basal bodies was calculated as the ciliation level.

Rotational polarity analysis

8-week filter samples were fixed with methanol as previously described, with basal bodies stained using POC1B antibodies and basal feet stained using centriolin antibodies. Samples were observed with 3D-SIM, and the maximum intensity projection of the reconstructed images was exported as ome.tiff (8 bit) for rotational polarity analysis in MATLAB (5). Briefly, binary images were first generated by applying a threshold to two channels. Individual basal body and basal foot were identified in the binary images, paired by pairwise nearest neighbour search, and filtered with a 600 nm cutoff. The basal body-basal foot pairs within one cell were determined by manually defining the contour of the cell with the MATLAB free drawing tool. The direction of each pair was the direction from the center of the basal body to the center of the basal foot. The cilia beat coordination in one cell was represented by the aligned vector length –an average of all basal body basal foot direction within one cell, with a value close to 1 meaning full coordination and 0 meaning no coordination.

STORM imaging and data analysis

For RPGR STORM imaging, cell suspensions were generated by digesting the filter samples with 0.025% Trypsin and resuspending disassociated cells in PneumaCult-Ex media. The cells were then spread and dried on coverslips (25 mm, No. 1.5 H, Deckglaser). The cells were immunostained with primary antibodies against RPGR (1:100 dilution), and then labeled with goat anti-rabbit F(ab')2 Alexa 647 (ThermoFisher Scientific, A21237). For actin STORM data, the filter sample was fixed with PFA and GA, and then labeled with Phalloidin-Alexa 647. The labeled sample was mounted with imaging buffer. The imaging buffer contains 50 mM Tris-HCl (pH 8.0), 10 mM NaCl, and 10% glucose, and is supplied with 50 mM β-mercaptoethanol (Sigma, 2661), 56 mg/mL glucose oxidase (Sigma, 3231), and 17 mg/mL catalase (Sigma, C9322). Concaved slides were used, and the imaging buffer was added between the coverslip and slide. The slide was sealed with nail polish. The labeled samples were imaged with an Abbelight SAFe360 setup with a 100x/1.50 Oil immersion objective lens, and the 647-

excitation laser (500 mW) power was set at 10% to collect the conventional image, and with 100% to switch the fluorophores to dark state and for STORM imaging. The 405 laser (100 mW) power was manually adjusted from 0 to 100% for fluorophore activation. The exposure time was set to 20-50 ms, and 60,000~200,000 frames were collected by a sCMOS Hamamatsu ORCA Fusion camera. Acquired STORM data was then processed by the Abbelight NEO Analysis software for Gaussian fitting. Briefly, after background estimation and removal, the detected molecules were fitted, and the corresponding lateral and axial positions of each activated fluorescent molecule were extracted. The super-resolution image was reconstructed based on the coordinates after drift correction by cross-correlation. The precision was averaged from the localization precisions for all the single-molecule events, and the resolution was around 20 nm.

STED imaging and data analysis

For phalloidin STED imaging, the phalloidin-Alexa 647 or phalloidin-STAR RED (Abberior, STRED-0100-20UG) labeled MCCs from the ALI filter were observed using the STEDYCON (Abberior) super-resolution microscope with a 100x/1.45 Oil immersion objective lens, and the ultra-short pulse red laser (800 μW) excitation power was set at 1%, and the 775 nm depletion laser (1.2 W) power was set at 20% to collect the STED datasets.

For dual labeling of F-actin and centrins, HBEC/HNC were transfected with lentivirus expressing centrin-GFP (pLVX-EGFP-C1-centrin-1 (Addgene, #73331)). For dual labeling of F-actin and RPGR, HBEC/HNC were first immunostained with RPGR antibodies, and then labeled with goat anti-rabbit Abberior STAR ORANGE (Abberior, STORANGE-1002) followed by phalloidin-STAR RED staining. The STAR ORANGE signals were observed with the ultra-short pulse green laser (561 nm, 100 μW), and the excitation power was set at 10%, and the 775 nm depletion laser (1.2 W) power was set at 10% to collect the STED datasets. The phalloidin-STAR RED signals were observed with the ultra-short pulse red laser (647 nm, 800 μW), and the excitation power was set at 0.5%, and the 775 nm depletion laser (1.2 W) power was set at 5% to collect the STED datasets.

Acquired STED data was then deconvoluted by the Huygens Professional Software.

Latrunculin A and Y27632 rescue experiment

hTERT-RPE1 control and *RPGR* KO cells were seeded on coverslips (25 mm diameter, thickness #1.5) with a density of 2.1 x 10^4 cells/cm^2, and 6 hours after cell seeding, the DMEM/F12 growth media was supplemented with 0.2 μM Latrunculin A (Santa Cruz, sc-202691) for another 24 hours. The control cells were treated with DMSO. Samples were then immunostained as described above with antibodies against acetylated tubulin, ARL13B, and DAPI. Skin fibroblast cell LatA treatment was the same as hTERT-RPE1 cells.

Airway basal cells were seeded on Transwell supports for 2-day proliferation, and differentiated at the air-liquid interface. The treatment of LatA and Y27632 started on ALI day 0. 0.1 μM LatA or 20 μM Y27632 was added into the ALI medium. At ALI week 4, samples were collected for measurement of cilia length and ciliation, and the analysis of cilia beat. As to later stage treatment, airway basal cells were seeded on Transwell supports for 2-day

proliferation, and differentiated at the air-liquid interface for 4 weeks, and switched to ALI medium containing 0.1 μM LatA or 20 μM Y27632 for another 4 weeks. After 4 weeks of treatment, samples were collected for further analysis.

For the drug withdrawal experiment, the treatment of LatA or Y27632 started on ALI day 0. After 4 weeks of treatment, the drug treatment was stopped, and only ALI medium was applied for differentiation. At ALI week 8, samples were collected for further analysis.

Rho Activator II treatment experiment

Airway basal cells were seeded on Transwell supports for 2-day proliferation, and differentiated at the air-liquid interface. The treatment of Rho Activator II (Cytoskeleton, CN03_B) started on ALI day 0. 0.5 μg/mL or 1 μg/mL Rho Activator II was added to the ALI medium, and the ALI medium containing Rho Activator II was changed every two days. At ALI week 8, samples were collected for measurement of cilia length and ciliation, and analysis of cilia beat.

G-actin/F-actin immunoblotting assay

The actin of cultured human airway MCCs was extracted according to the manufacturer's protocol (Cytoskeleton, BK037). Briefly, 2-filter samples of 24-transwell insert were collected with 100 μL LAS2 buffer, and after sonication and incubation, F-actin and G-actin were separated by ultracentrifugation (100,000g, 37 °C, 2 hrs). For Western blot analysis, the same volume of F-actin and G-actin was loaded on 4-20% FuturePAGE Gel (ACE, ET12420GEL), and then transferred onto PVDF membrane (Cytiva, 10600023). The membrane was blocked with 5% non-fat milk (Bio-Rad, 706404) in TBST (10 mM Tris-HCl (pH 8.0), 150 mM NaCl, 0.01% Tween 20) for 30 minutes, and incubated with anti-mouse monoclonal actin antibody at a dilution of 1:500 overnight at 4°C. The membrane was stained with goat anti-mouse-HRP secondary antibodies (dilution of 1:2000) for 2 hours (ThermoFisher, 31430). HRP blots were developed with Clarity Western ECL Substrate (Bio-Rad, 1705060), and imaged using Touch Imager XLI (e-BLOT). The quantification was performed with ImageJ.

Cilia beat analysis

Cilia beating leads to oscillation of signal intensity over time at cilia-localized pixels. The contour of the ciliated cells was defined by applying an intensity threshold (set as the mean of the whole plot) to the maximum intensity projection plot and a Gaussian smoothing. The frequency of oscillations for every pixel of the recording was analyzed by computing the Fourier transform of the corresponding intensity changes over time using the MATLAB fast Fourier transform function (fft()). For each pixel, cilia beat frequency (CBF) was calculated as the frequency where the absolute value of the Fourier transform was maximal. This resulted in a scaled spatial CBF map. To reduce background signal fluctuation noise, we listed all connected pixels in the frequency map using the bwconncomp() function and removed regions with fewer than 144 pixels (6, 7). Similarly, we defined regions of interest (ROIs) where the intensity change could reflect the single cell cilia beat frequency manually using the ImageJ plugin ROI Manager. The ROI sizes are variable depending on the actual cell size. A similar

FFT process was performed in R to generate the CBFs. For samples with fewer ciliated cells, we tried to take all cells into account and randomly picked >30 cells in highly ciliated samples. For cilia beat mode analysis, the end-on-view WGA-488 labeled cilia beat videos were analyzed manually. Except the normal beating, four other different types of beat mode can be identified: 1) static cilia; 2) restricted beat, which means cilia beat is restricted with pretty low amplitude; 3) rigid beat, which means that cilia beat loses power and recovery stroke pattern; 4) uncoordinated beat, which means cilia beat in rotationary or/and uncoordinated manner. For each ROI, the cell number for each beat mode was counted, and the ratio was calculated by dividing the cell number for each beat mode by the total cell number.

Single-cell sequencing and data analysis

We modified the GRCh38 ENSEMBL reference by deleting *RPGR* gene-level annotation and treating each *RPGR* transcript isoform as a separate gene. A new genome reference was then constructed using Cell Ranger (v7.0.0) (8) (The code for this customization is available at https://github.com/Jiayi-Zheng/Build-Gene-Isoform-Reference-for-10X).

Sequence alignment was subsequently performed on a previously published human fetal tracheal scRNA-seq dataset (gestation week 21) (9). The dataset was processed and further divided to exclude non-epithelial cells with Seurat (v5.0.0), and VlnPlot() was used to visualize the raw count distribution of *RPGR* transcripts across various cell types.

RNA extraction and RT-PCR/qRT-PCR

The total RNA of cultured human airway MCCs was extracted and reverse-transcribed according to the manufacturer's protocol (Vazyme, RC112, and R412).

For RT-PCR, the obtained cDNA was amplified using the following primers to target *RPGR*^{ORF15} region: CAGATGAGGAAGTAGAGATCCCAGAG and reverse primer (10): CTCTCCTTCCTCCTTTTCAC. The obtained product was assessed by DNA gel electrophoresis and Sanger sequencing. The amplified band with primers of GAPDH was applied as the loading control.

For qRT-PCR, cDNA and FastStart Universal SYBR Green Master (Roche, 4913850001) were mixed according to the manufacturer's protocol. *FOXJ1* and *CCNO* cDNA fragments were amplified using the Roche LightCycler 480 Realtime PCR System. Two sets of primers were designed to amplify the *FOXJ1* and *CCNO* cDNA respectively: 1) *FOXJ1*-Primer 1 forward primer: CCCGACGACGTGGACTA and reverse primer: GGCGGAAGTAGCAGAAGTTG (11), 2) *FOXJ1*-Primer 2 forward primer: CCCACCTGGCAGAATTCAATCCG and reverse primer: CTCAGTAGCCGCTCCGCGTAC (12), 3) *CCNO*-Primer 1 forward primer: GGCGGAATCCCGCTGTAAG and reverse primer: GTCCAGAGTGTTCACCGTCA, 4) *CCNO*-Primer 2 forward primer: TTCACCAGCTACTCCCCTTCC and reverse primer: TCTCCCAGTCGCAAGTCCA. 18s RNA were amplified using the forward primer: GGCCCTGTAATTGGAATGAGTC and reverse primer: CCAAGATCCAACTACGAGCTT (13). For mRNA expression quantification, the fold changes in gene expression were calculated using the delta-delta Ct method and normalized with 18S.

Western blot

The human airway MCCs were lysed with RIPA buffer (ThermoFisher, 89900), supplemented with protease inhibitor (Abcam, ab271306) by incubation on ice for 30 minutes. Total protein extract from the supernatant was quantified with Pierce BCA Protein Assay Kit (ThermoFisher, 23227). For Western blot analysis, 10 µg total protein extract was loaded on a 4-20% FuturePAGE gel, and then transferred onto a 0.45 µm PVDF membrane. Membrane was blocked with 5% non-fat milk in TBST for 1 hour, and incubated with primary antibody overnight at 4℃. Then, the membrane was stained with goat anti-rabbit-HRP secondary antibodies at a dilution of 1:2000 for 2 hours (ThermoFisher, 31460). HRP blots were developed with Clarity Western ECL Substrate (Bio-Rad, 1705060), and imaged using Touch Imager XLI (e-BLOT). For protein quantification, densitometry was performed with ImageJ and normalized with internal histone expression.

TEM

Human nasal cells were scraped and fixed in 2% glutaraldehyde diluted in 100 mM sodium cacodylate, dehydrated with 200 mM D-sucrose dissolved in 100 mM sodium cacodylate. The samples were post-fixed with 1% OsO4, dehydrated with the graded ethanol, and finally embedded in resin. Samples were sliced to a thickness of ~90 nm and imaged with the FEI Tecnai 20 TEM.

References and Notes

1. Zhang Q, et al. Disruption of RPGR protein interaction network is the common feature of RPGR missense variations that cause XLRP. *Proc Natl Acad Sci USA*. 2019;116(4):1353–1360.

2. Horani A, et al. Rho-Associated Protein Kinase Inhibition Enhances Airway Epithelial Basal-Cell Proliferation and Lentivirus Transduction. *Am J Respir Cell Mol Biol*. 2013;49(3):341–347.

3. Chu HW, et al. CRISPR–Cas9-mediated gene knockout in primary human airway epithelial cells reveals a proinflammatory role for MUC18. *Gene Ther*. 2015;22(10):822–829.

4. Everman JL, et al. Functional genomics of CDHR3 confirms its role in HRV-C infection and childhood asthma exacerbations. *J Allergy Clin Immunol*. 2019;144(4):962–971.

5. Liu Z, et al. A quantitative super-resolution imaging toolbox for diagnosis of motile ciliopathies. *Sci Transl Med*. 2020;12(535):eaay0071.

6. Ringers C, et al. Novel analytical tools reveal that local synchronization of cilia coincides with tissue-scale metachronal waves in zebrafish multiciliated epithelia. *eLife*. 2023;12:e77701.

7. Feriani L. Understanding the Collective Dynamics of Motile Cilia in Human Airways.

8. Hao Y, et al. Dictionary learning for integrative, multimodal and scalable single-cell analysis. *Nat Biotechnol*. 2024;42(2):293–304.

9. He M, et al. Chloride channels regulate differentiation and barrier functions of the mammalian airway. *eLife*. 2020;9:e53085.

10. Hadjebi O, et al. The RCC1 superfamily: From genes, to function, to disease. *Biochimica et Biophysica Acta (BBA) - Molecular Cell Research*. 2008;1783(8):1467–1479.

11. Brody SL, et al. Undocking of an extensive ciliary network induces proteostasis and cell fate switching resulting in severe primary ciliary dyskinesia. *Sci Transl Med*. 2025;17(783):eadp5173.

12. Jain A, et al. Mitochondrial uncoupling proteins protect human airway epithelial ciliated cells from oxidative damage. *Proc Natl Acad Sci USA*. 2024;121(10):e2318771121.

13. Zhang L-S, et al. ALKBH7-mediated demethylation regulates mitochondrial polycistronic RNA processing. *Nat Cell Biol*. 2021;23(7):684–691.

Supplementary figures

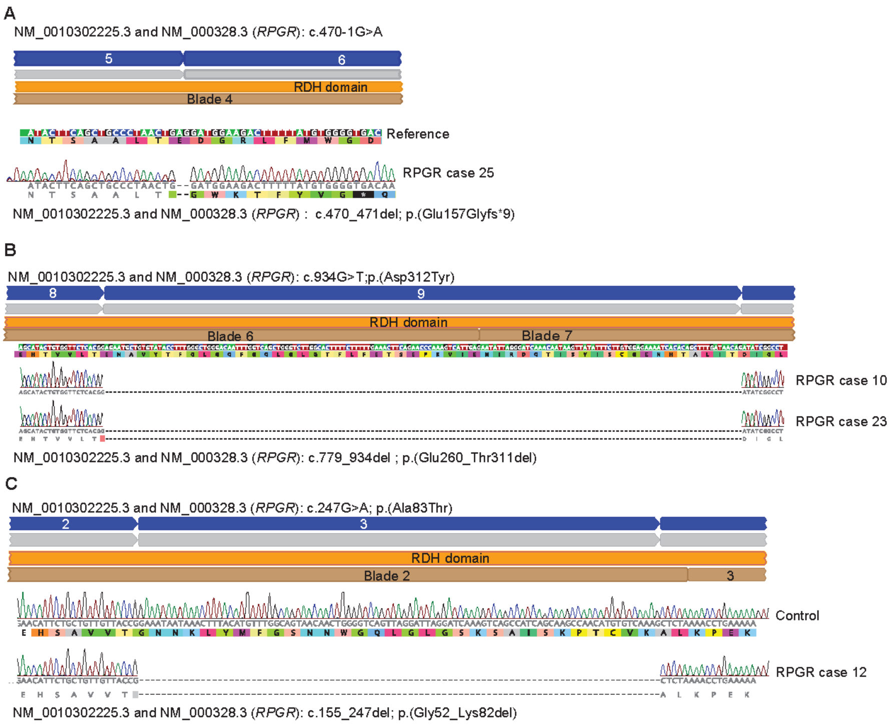

Supplemental Figure 1. Validation of the predicted variants through Sanger sequencing. (A) Sanger sequencing of the PCR products from the cell lines derived from case 25. **(B)** Sanger sequencing of the PCR products from the cell lines derived from cases 10 and 23. **(C)** Sanger sequencing of the PCR products from the cell lines derived from case 12.

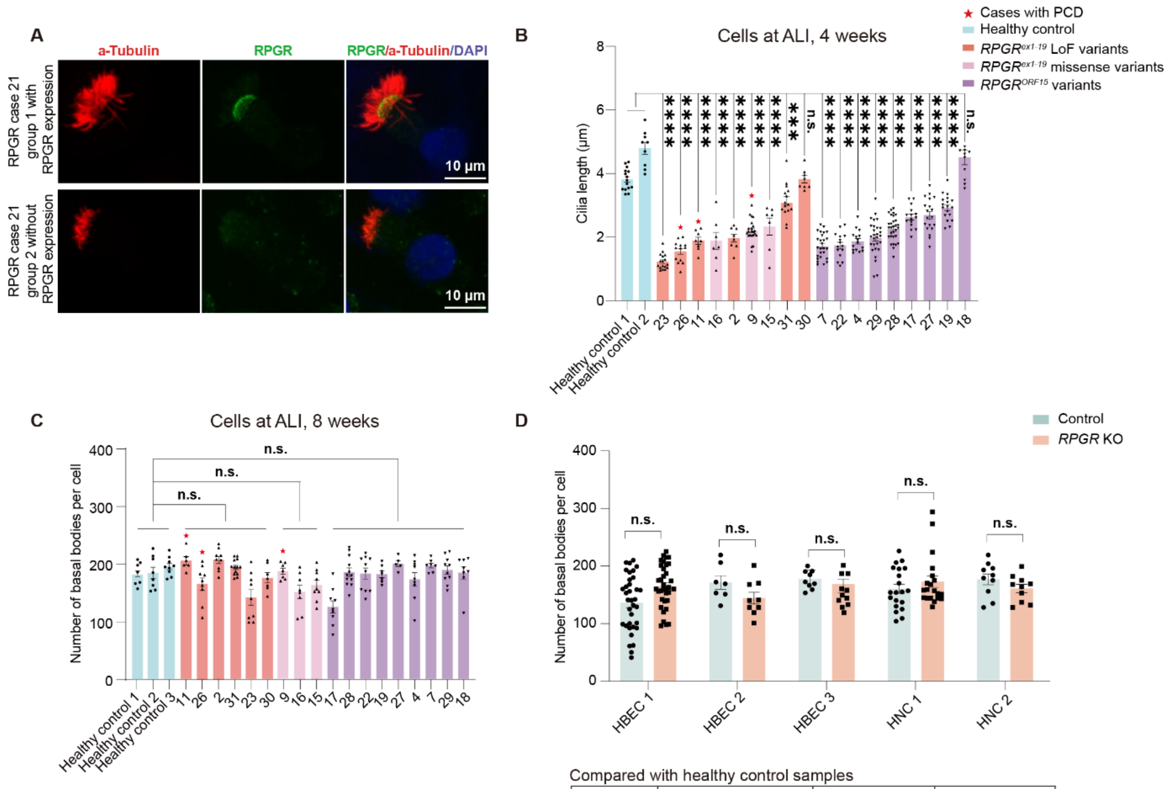

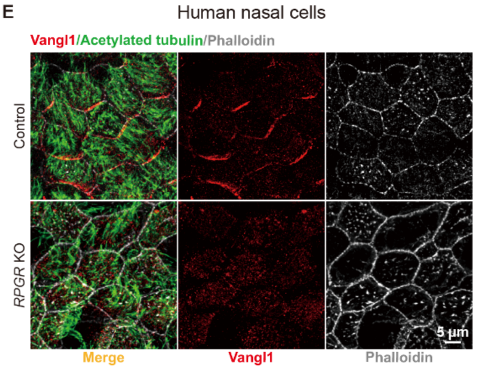

F

Compared with healthy control samples

Patient#	Genotype	Vangl1 (4 weeks)	Vangl1 (8 weeks)
11	*RPGR*$^{ex1-19}$ variant	diminished	diminished
26	*RPGR*$^{ex1-19}$ variant	diminished	NA
31	*RPGR*$^{ex1-19}$ variant	diminished	no change
23	*RPGR*$^{ex1-19}$ variant	diminished	NA
30	*RPGR*$^{ex1-19}$ variant	diminished	diminished
9	*RPGR*$^{ex1-19}$ variant	diminished	diminished
16	*RPGR*$^{ex1-19}$ variant	diminished	diminished
15	*RPGR*$^{ex1-19}$ variant	diminished	diminished
22	*RPGR*^{ORF15} variant	diminished	diminished
27	*RPGR*^{ORF15} variant	diminished	NA
19	*RPGR*^{ORF15} variant	diminished	NA
4	*RPGR*^{ORF15} variant	diminished	NA
18	*RPGR*^{ORF15} variant	diminished	diminished
7	*RPGR*^{ORF15} variant	diminished	diminished
17	*RPGR*^{ORF15} variant	diminished	NA
28	*RPGR*^{ORF15} variant	diminished	diminished
29	*RPGR*^{ORF15} variant	diminished	NA

Supplemental Figure 2. MCCs with *RPGR* defects presented with reduced cilia length, and disrupted planar polarity. (A) Immunostaining of RPGR and cilia marker a-tubulin showed that MCCs for case 21 with *RPGR* expression presented normal cilia length, and those without *RPGR* expression presented decreased cilia length. Scale bar, 10 µm. **(B)** Cilia length measurements for the MCCs from both controls and RP patients cultured at the air-liquid interface for 4 weeks. **(C)** The number of basal bodies was unchanged for most MCCs bearing damaging variants. **(D)** The number of basal bodies was unaffected for all 5 *RPGR* KO biological replicates. **(E)** Immunostaining of Vangl1, acetylated tubulin, and phalloidin showed Vangl1 mislocalization for *RPGR* KO HNCs. Scale bar, 5 µm. (**F**) A summary of Vangl1 distribution in patient MCCs. Data represent mean ± SEM. n.s. no significance, ***, $p < 0.001$, ****, $p < 0.0001$ by two-tailed t-test **(B, D)** or two-way repeated ANOVA followed by Sidak's post hoc test **(C)**.

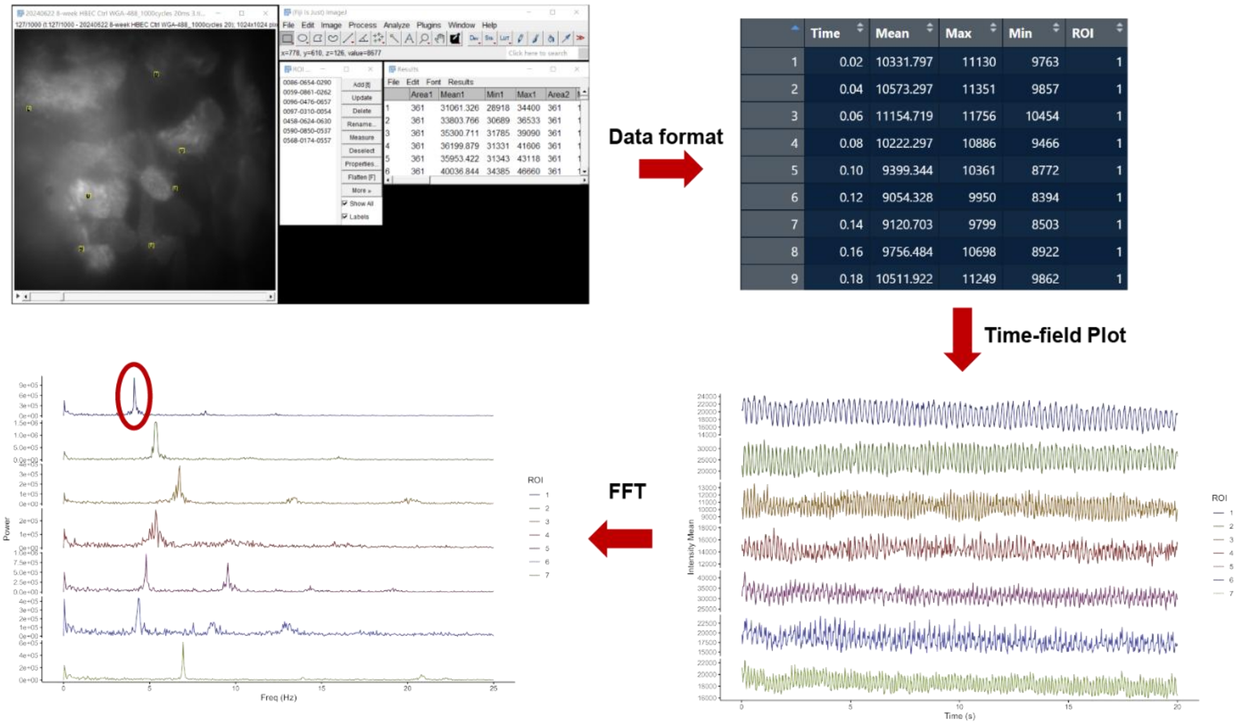

Supplemental Figure 3. Workflow of single-cell cilia beat frequency analysis. The peak frequency was generated from the manually acquired intensity spectrum by fast Fourier transform, and was designated as the cilia beat frequency of the cell.

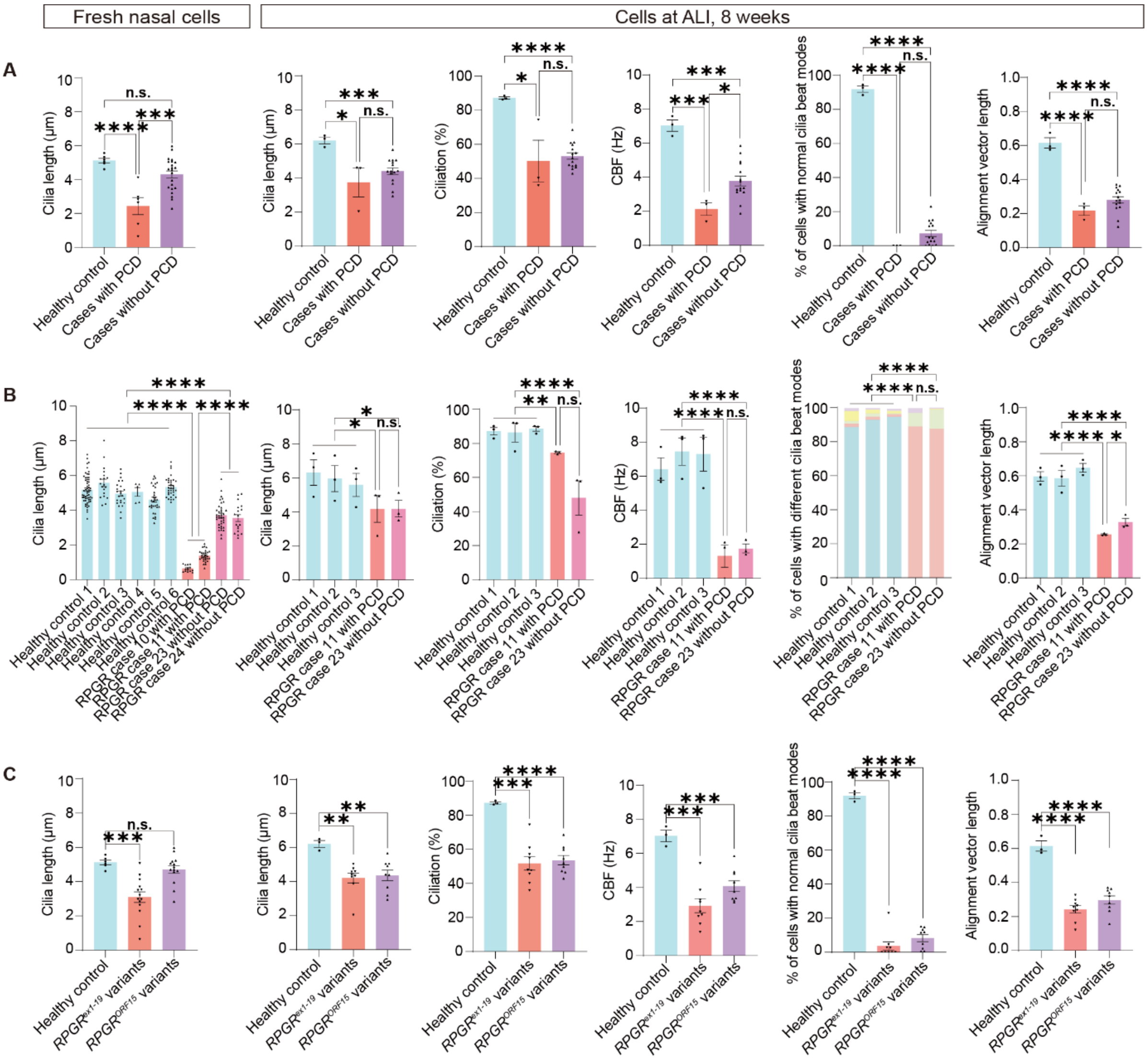

Supplemental Figure 4. Comparison of motile cilia properties across different patient groups. (A) Analysis of motile cilia characteristics in patients with PCD (cases 9, 10, 11, 20, 25, and 26) versus those without PCD. Each point represents the mean value of one individual. **(B)** Analysis of the motile cilia characteristics in patients sharing the same *RPGR* variant (c.934G>T; p.(Glu260_Thr311del), cases 10, 11, 23, and 24). Each point represents the mean data of one control or one patient. **(C)** Analysis of motile cilia characteristics in patients with *RPGR*$^{ex1\text{-}19}$ variants versus those with *RPGR*^{ORF15} variants. Each point represents the mean data of one control or one patient. Data represent mean ± SEM. n.s. no significance, *, $p < 0.05$, **, $p < 0.01$, ***, $p < 0.001$, ****, $p < 0.0001$ by two-tailed t-test **(A, C)** or two-way repeated ANOVA followed by Sidak's post hoc test **(B)**.

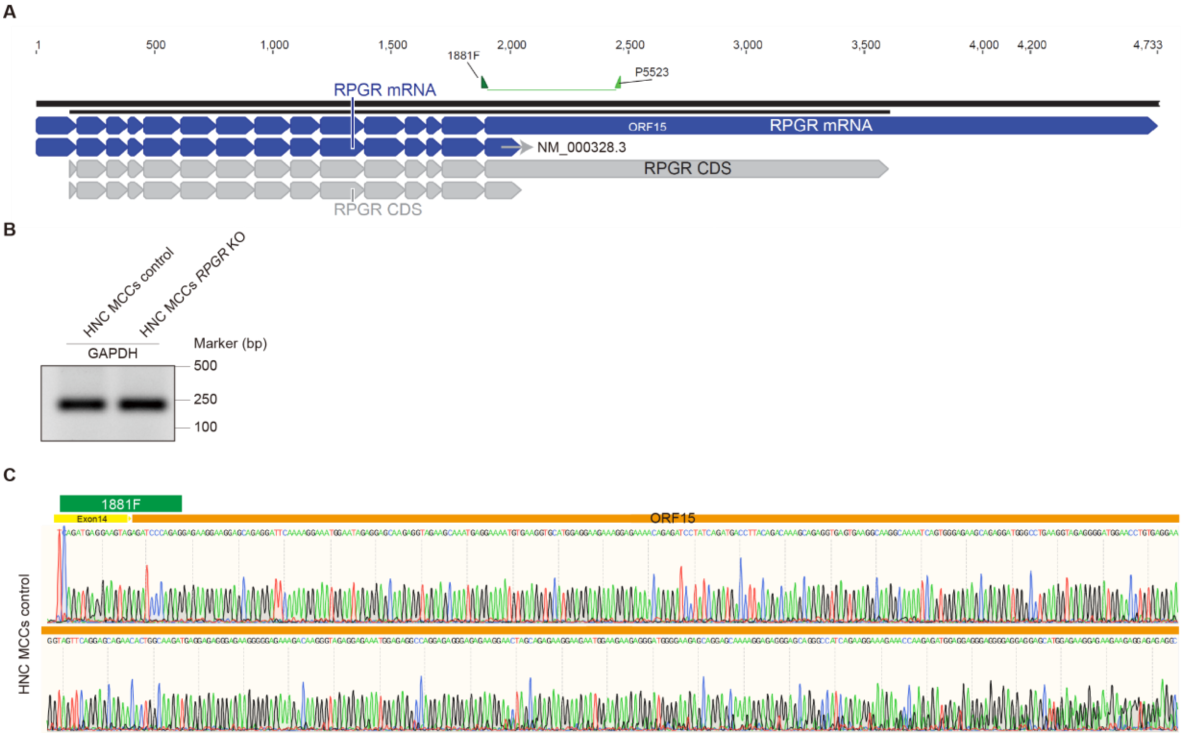

Supplemental Figure 5. Validation of the expression of *RPGR*^{ORF15} isoform in human airway MCCs. (A) RT-PCR primer design for the detection of the *RPGR*^{ORF15} isoform. **(B)** Loading control for the RT-PCR result shown in Figure 2J. **(C)** The amplified RT-PCR band (Figure 2J) was purified and validated by Sanger sequencing.

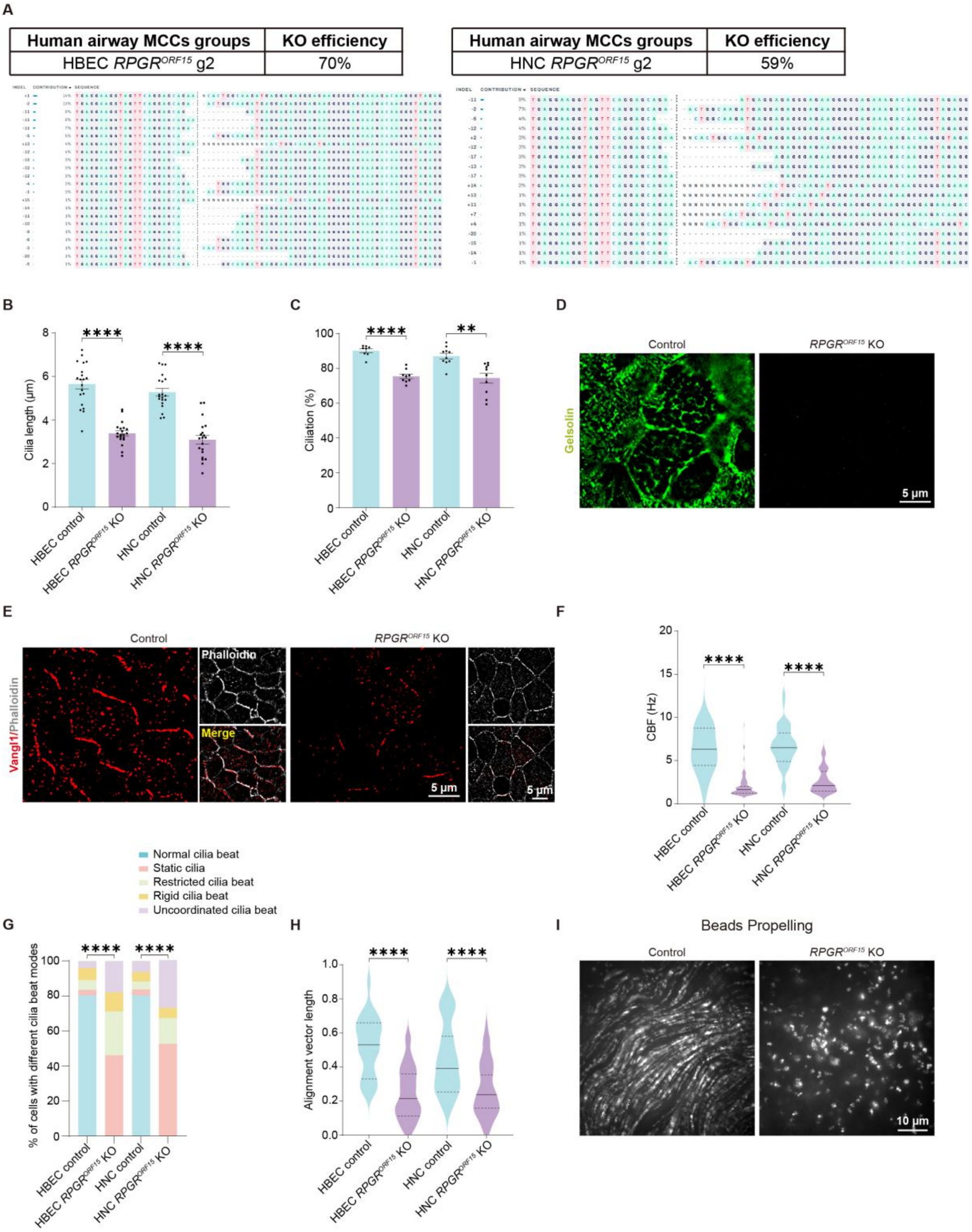

Supplemental Figure 6. Characterization of *RPGR^ORF15^* KO MCCs generated with an ORF15-specific gRNA. (A) DNA sequencing showed efficient KO of *RPGR^ORF15^* in airway MCCs. **(B)** Cilia length was significantly reduced for the *RPGR^ORF15^* KO MCCs. **(C)** Ciliation was significantly decreased for the *RPGR^ORF15^* KO MCCs. **(D)** Gelsolin failed to locate to the apical surface for the 4-week *RPGR^ORF15^* KO MCCs. Scale bar, 5 μm. **(E)** Vangl1 failed to establish the apical distribution in *RPGR^ORF15^* KO MCCs. Scale bar, 5 μm. The insert, 5 μm. **(F)** Cilia beat frequency was significantly diminished in the *RPGR^ORF15^* KO MCCs. Results were summarized from 2 biological replicates (1 HBEC sample and 1 HNC

sample). **(G)** The classic waveform was significantly disrupted in the *RPGR*^{ORF15} KO. **(H)** Rotational polarity was significantly impaired in the *RPGR*^{ORF15} KO MCCs. **(I)** Beads propelling was abolished in 8-week *RPGR*^{ORF15} KO MCCs. The image showed the maximum intensity projection of the video recordings of bead movement. Scale bar, 10 μm. Results were summarized from 2 biological replicates (1 HBEC sample and 1 HNC sample). Data represent mean ± SEM. The center, upper, and lower lines represent the median, upper, and lower quartiles, respectively **(F, H)**. **, $p < 0.01$, ****, $p < 0.0001$ by two-tailed t-test **(B, C, F, H)** or Fisher's exact test **(G)**.

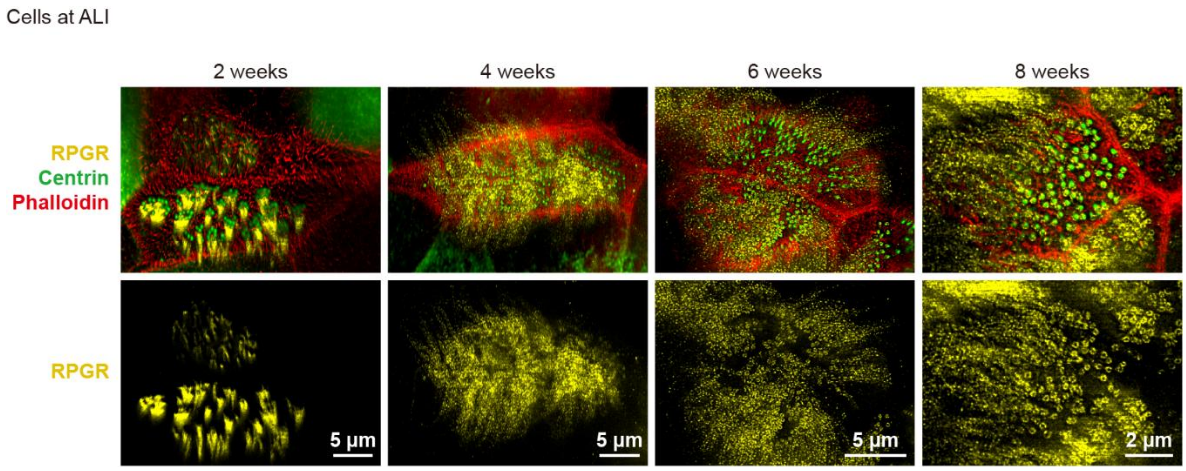

Supplemental Figure 7. RPGR is also located in motile cilia throughout the differentiation of MCCs. Scale bar, 5 μm and 2 μm.

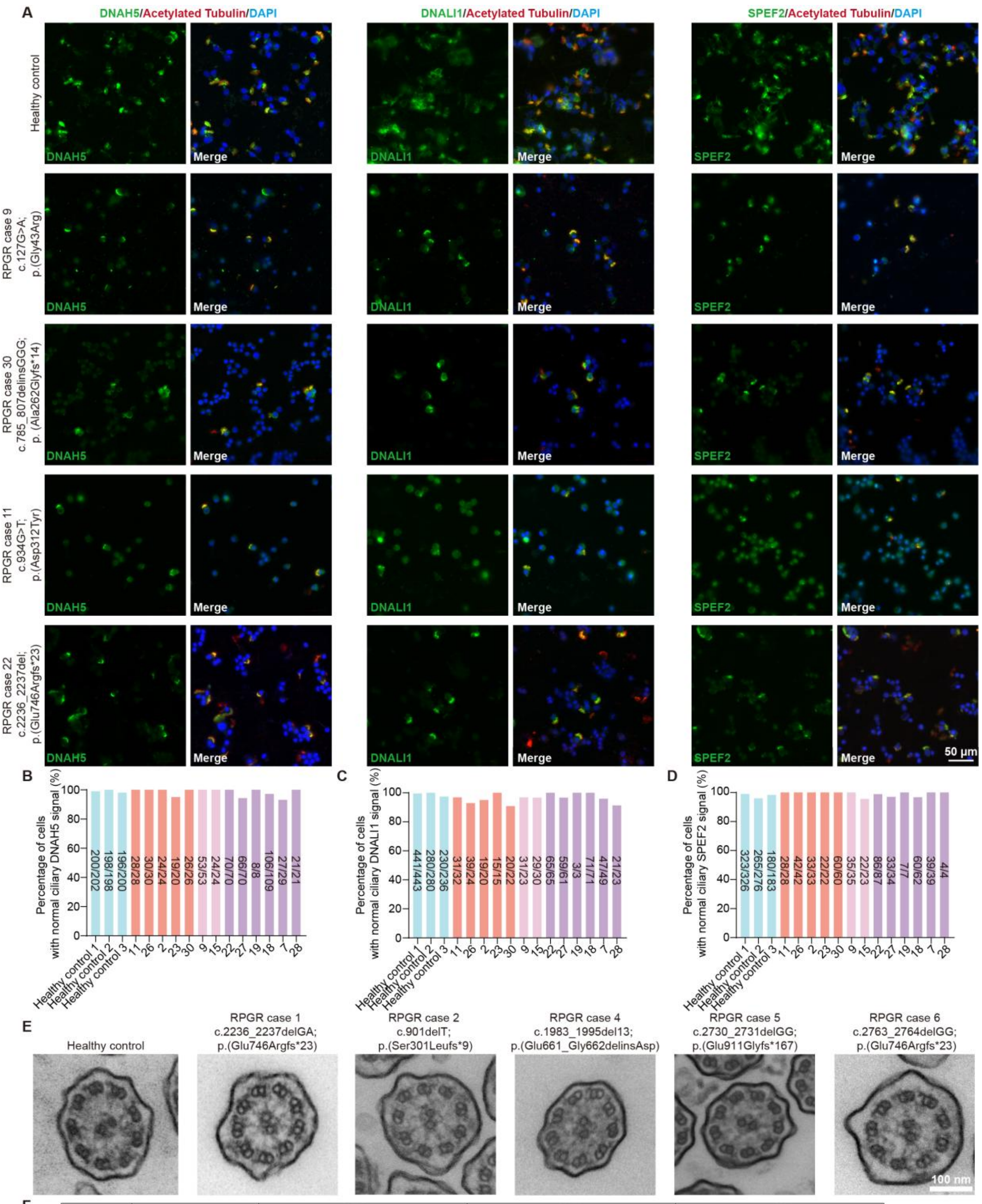

F

Patient#	Genotype	ODA		IDA		CP	
		Total cilia number	ODA count	Total cilia number	IDA count	Total cilia number	abnormal CP ratio
1	*RPGR*$^{ex1-19}$ variant	80	8.8 (range 7 to 9)	80	5.9 (range 3 to 9)	80	NO
2	*RPGR*^{ORF15} variant	60	8.1 (range 3 to 9)	60	6.4 (range 3 to 9)	60	~10%
5	*RPGR*^{ORF15} variant	60	8.9 (range 8 to 9)	60	4.8 (range 2 to 9)	60	NO
32	*RPGR*^{ORF15} variant	80	8.6 (range 5 to 9)	80	7.1 (range 4 to 9)	80	~1%
10	*RPGR*$^{ex1-19}$ variant	NA	NA	NA	NA	NA	NA
11	*RPGR*$^{ex1-19}$ variant	NA	NA	NA	NA	NA	NA

Supplemental Figure 8. RPGR defect didn't affect the distribution of ODA, IDA, or CP components. (A) Immunostaining of ODA marker DNAH5, IDA marker DNALI1, central pair

marker SPEF2, and cilia marker acetylated-tubulin showed that the MCCs with either $RPGR^{ex1-19}$ or $RPGR^{ORF15}$ variants maintained intact ODA, IDA, and central pair components. Note that case 9 had shorter cilia, while case 30 had longer cilia; most cilia in case 11 had a low beating frequency, whereas case 22's cilia had a higher frequency. Scale bar, 50 μm. **(B-D)** A summary of the IF staining data for 13 patients. **(E)** TEM showed the normal ciliary ultrastructure for human MCCs with different *RPGR* variants. Scale bar, 100 nm. **(F)** A summary of the TEM experiments performed.

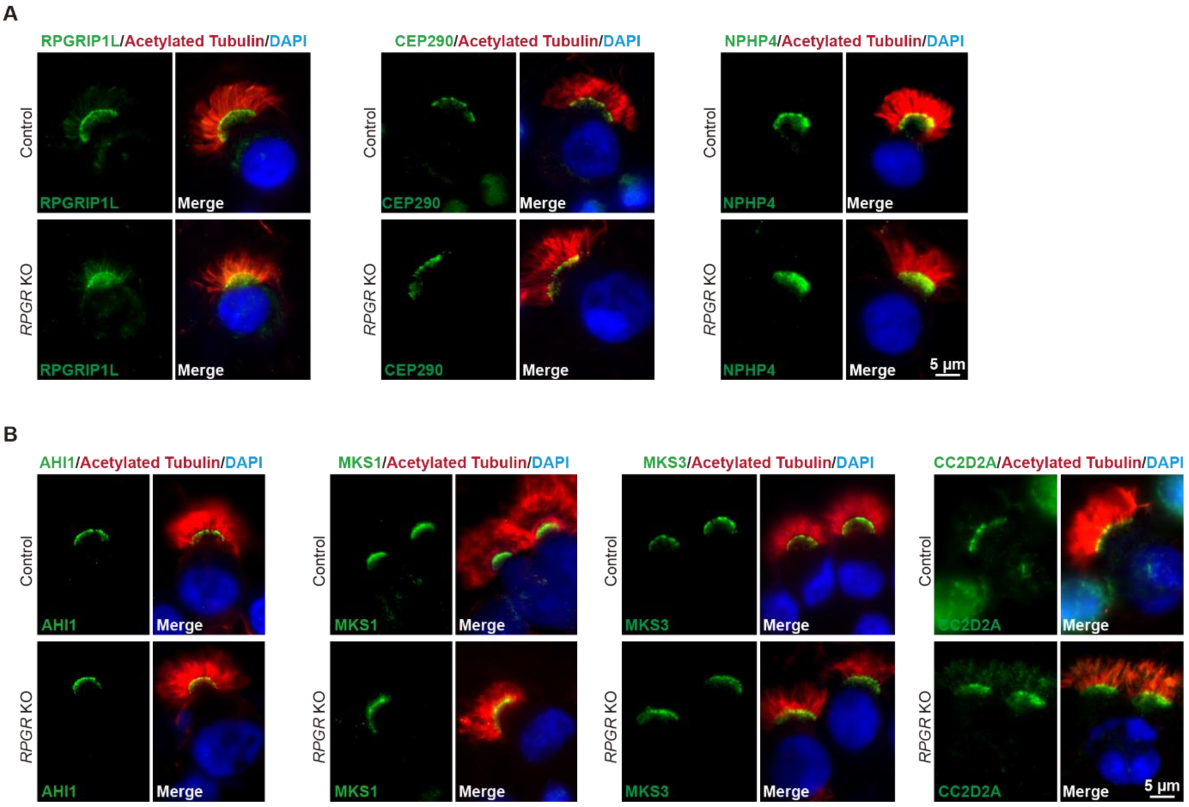

Supplemental Figure 9. The subcellular localization of RPGR interactors and transition zone components was largely unaffected in *RPGR* KO MCCs. (A) Immunostaining of RPGR interactors RPGRIP1L, CEP290, or NPHP4 showed that their TZ distributions were not affected in *RPGR* KO MCCs. Scale bar, 5 μm. **(B)** Immunostaining of transition zone components AHI1, MKS1, MKS3, or CC2D2A showed that the transition zone structure was preserved in *RPGR* KO MCCs. Scale bar, 5 μm.

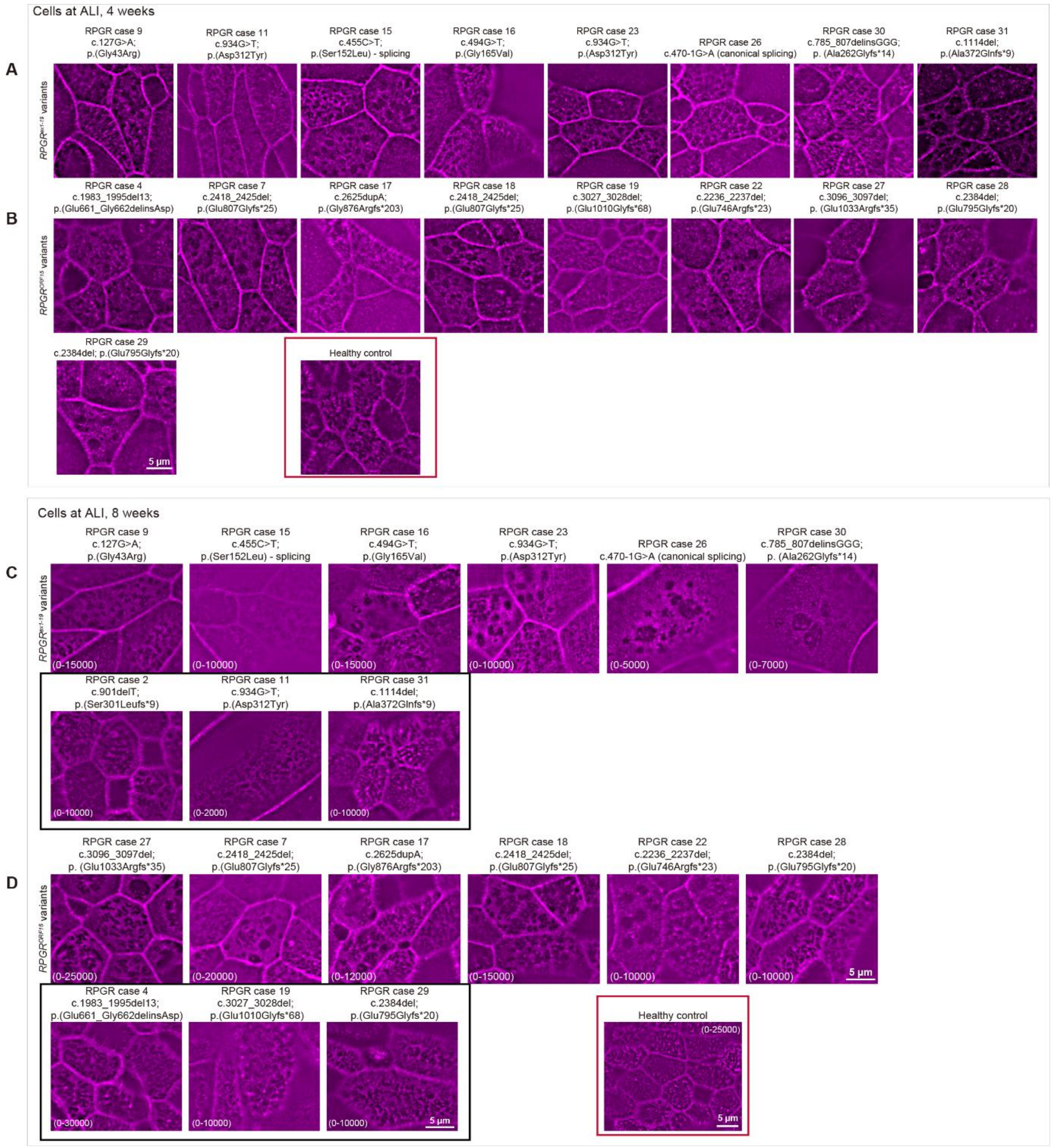

Supplemental Figure 10. Apical F-actin meshwork accumulated in the MCCs from RP patients with pathological variants in *RPGR*. (A) The F-actin meshwork for the 4-week MCCs from patients with *RPGR*$^{ex1-19}$ variants. **(B)** The F-actin meshwork for the 4-week MCCs from patients with *RPGR*^{ORF15} variants. The red square highlights the control as a comparison. Scale bar, 5 µm. **(C)** Distribution of F-actin in 8-week patient MCCs with pathological variants in *RPGR*$^{ex1-19}$. The black rectangle highlights the patient cells without any changes compared to control cells. **(D)** Distribution of F-actin in 8-week patient MCCs bearing *RPGR*^{ORF15} variants. The red square highlights the control as a comparison. The black rectangle highlights the patient cells without any changes compared to control cells. Scale bar, 5 µm.

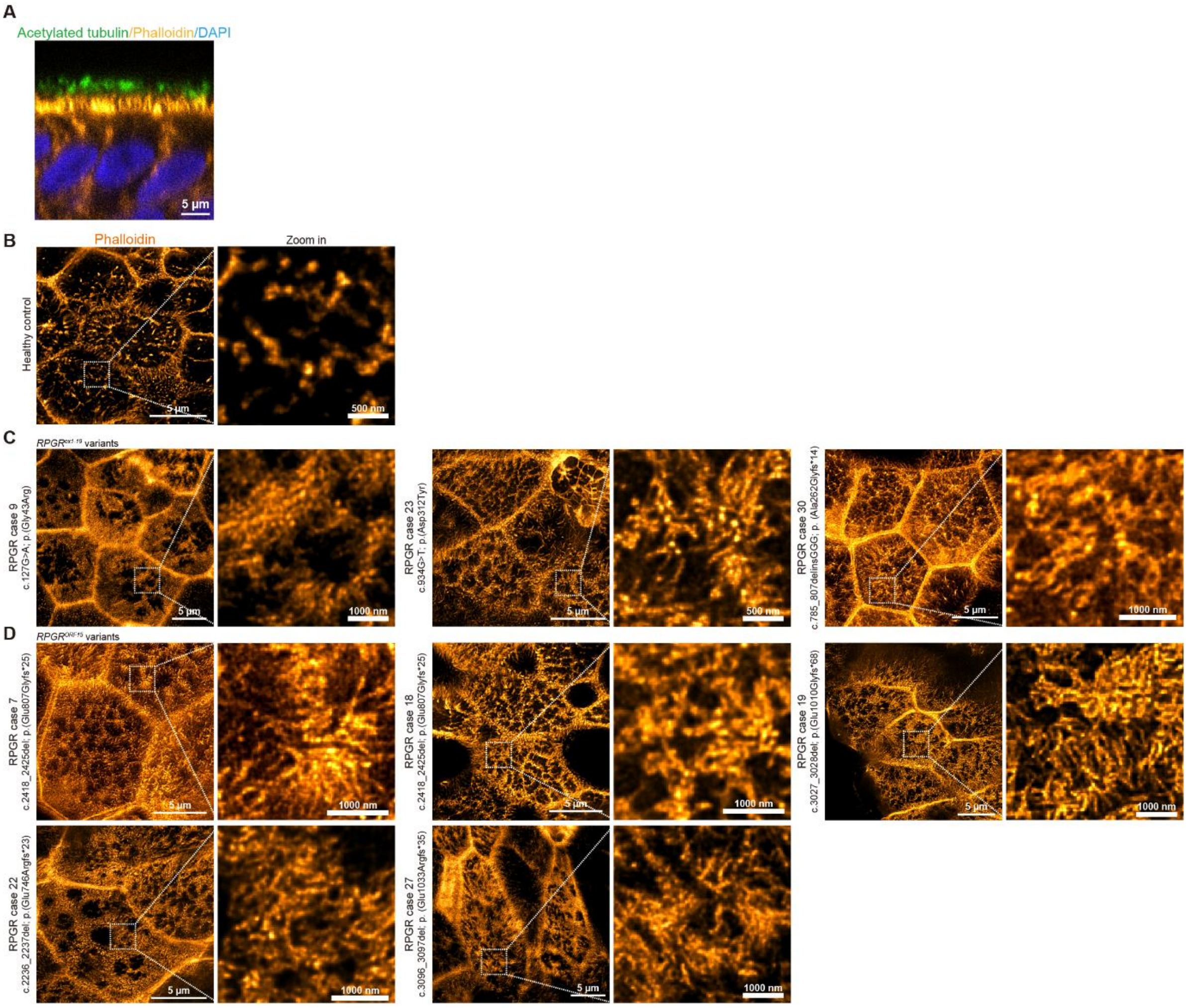

E Compared with healthy control samples

Patient#	Genotype	F-actin (SIM observation) (4 weeks)	F-actin (SIM observation) (8 weeks)	F-actin (STED observation) (8 weeks)
11	*RPGR*$^{ex1-19}$ variant	no change	no change	NA
26	*RPGR*$^{ex1-19}$ variant	no change	accumulation	NA
2	*RPGR*$^{ex1-19}$ variant	NA	no change	NA
31	*RPGR*$^{ex1-19}$ variant	no change	no change	NA
23	*RPGR*$^{ex1-19}$ variant	no change	accumulation	accumulation
30	*RPGR*$^{ex1-19}$ variant	no change	accumulation	accumulation
9	*RPGR*$^{ex1-19}$ variant	no change	accumulation	accumulation
16	*RPGR*$^{ex1-19}$ variant	no change	accumulation	NA
15	*RPGR*$^{ex1-19}$ variant	no change	accumulation	NA
22	*RPGR*^{ORF15} variant	no change	accumulation	accumulation
27	*RPGR*^{ORF15} variant	no change	accumulation	accumulation
19	*RPGR*^{ORF15} variant	no change	no change	accumulation
4	*RPGR*^{ORF15} variant	no change	no change	NA
18	*RPGR*^{ORF15} variant	no change	accumulation	accumulation
7	*RPGR*^{ORF15} variant	no change	accumulation	accumulation
17	*RPGR*^{ORF15} variant	no change	accumulation	NA
28	*RPGR*^{ORF15} variant	no change	accumulation	NA
29	*RPGR*^{ORF15} variant	no change	no change	NA

Supplemental Figure 11. STED revealed that apical F-actin meshwork accumulated in the MCCs from RP patients with pathological variants in *RPGR*. (A) An x-z cross-section image shows the apical F-actin located at the base of cilia. Scale bar, 5 μm. **(B)** The apical F-actin in the healthy control MCCs cultured at ALI for 8 weeks. Scale bar, 5 μm. The insert scale

bar, 500 nm. **(C)** The F-actin meshwork for the MCCs from patients with *RPGR*$^{ex1\text{-}19}$ variants cultured at ALI for 8 weeks. Scale bar, 5 µm. The insert, 500 nm and 1000 nm. **(D)** The F-actin meshwork for the MCCs from patients with *RPGR*^{ORF15} variants cultured at ALI for 8 weeks. Scale bar, 5 µm. The insert, 1000 nm. **(E)** A summary of the F-actin distribution experiments in patient MCCs. For the majority of the 8-week patient MCCs (13 out of 18 patients), F-actin accumulated at the apical surface.

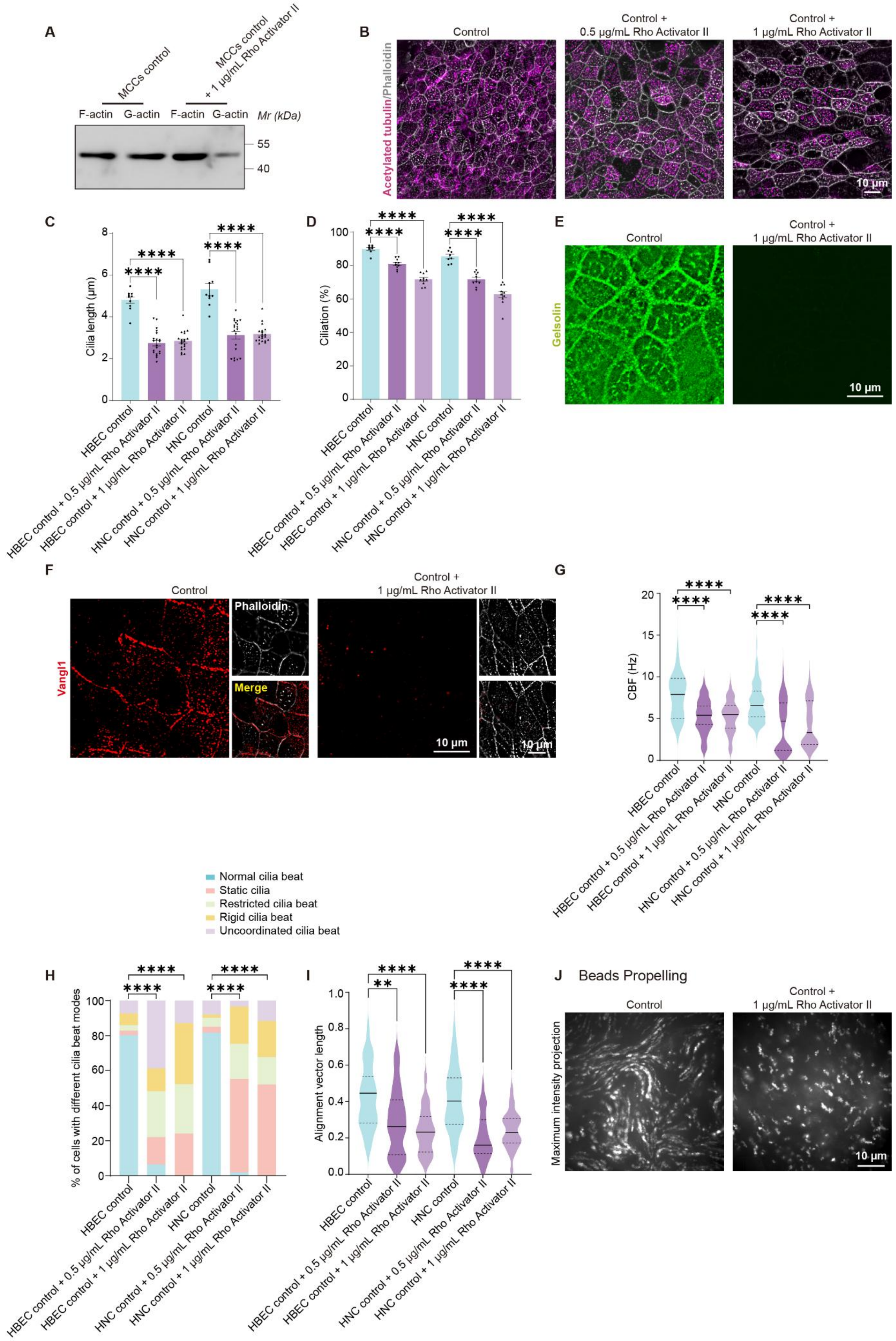
A
MCCs control
MCCs control + 1 μg/mL Rho Activator II
F-actin
G-actin
F-actin
G-actin
Mr (kDa)
55
40
B
Control
Control + 0.5 μg/mL Rho Activator II
Control + 1 μg/mL Rho Activator II
Acetylated tubulin/Phalloidin
10 μm
C
Cilia length (μm)
D
Ciliation (%)
E
Control
Control + 1 μg/mL Rho Activator II
Gelsolin
10 μm
F
Control
Control + 1 μg/mL Rho Activator II
Vangl1
Phalloidin
Merge
10 μm
G
CBF (Hz)
Normal cilia beat
Static cilia
Restricted cilia beat
Rigid cilia beat
Uncoordinated cilia beat
H
% of cells with different cilia beat modes
I
Alignment vector length
J
Beads Propelling
Control
Control + 1 μg/mL Rho Activator II
Maximum intensity projection
10 μm
HBEC control
HBEC control + 0.5 μg/mL Rho Activator II
HBEC control + 1 μg/mL Rho Activator II
HNC control
HNC control + 0.5 μg/mL Rho Activator II
HNC control + 1 μg/mL Rho Activator II
****
**

489

Supplemental Figure 12. Stabilizing F-actin in healthy control MCCs affected multiciliogenesis and ciliary motility. (A) Immunoblotting showed Rho activator II treatment effectively increased the F-actin/G-actin ratio; Control MCCs cultured at ALI were treated with 1 μg/ml Rho activator II for 8 weeks before lysis for immunoblotting. **(B)** Immunostaining showed a reduction in both ciliation and cilia length in Rho activator II treated control MCCs. Scale bar, 10 μm. **(C, D)** Quantitative analysis showed a significant decrease in cilia length and ciliation in control MCCs treated with Rho activator II. n>8 cells per sample, and each point represents one cell. **(E)** Gelsolin failed to locate to the apical surface in 4-week control MCCs treated with Rho activator II. Scale bar, 10 μm. **(F)** Vangl1 failed to establish the apically polarized distribution in control MCCs treated with Rho activator II. Scale bar, 10 μm. The insert scale bar is 10 μm. **(G-J)** Cilia beat frequency **(G)**, waveform **(H)**, and rotational polarity **(I)** were all adversely affected in Rho Activator II treated MCCs, resulting in paralyzed clearance of fluorescent beads **(J)**. **(J)** The image shows the MIP of the video recordings of bead movement. Scale bar, 10 μm. For (G), n>42 cells per sample, and each point represents one cell. For (H), n>50 cells per sample, n>3 views per group, and each point represents one view. For (I), n>24 cells per sample, and each point represents one cell. The samples used were 8-week HNC/HBEC treated with 1 μg/ml Rho activator II. Results were from 2 biological replicates. Data represent mean ± SEM. The center, upper and lower lines represent the median, upper, and lower quartiles, respectively **(H)**. **, $p < 0.01$, ****, $p < 0.0001$ by two-tailed t-test **(C, D, G, I)** or Fisher's exact test **(H)**.

A

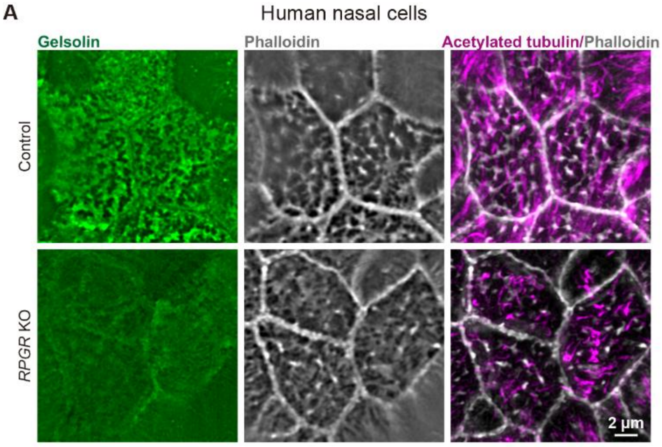

B

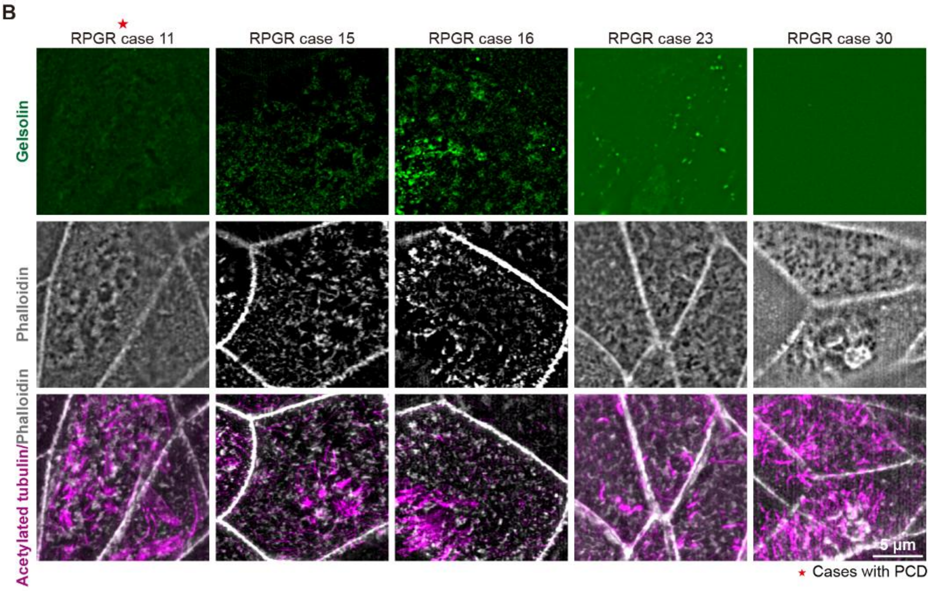

C Compared with healthy control samples

Patient#	Genotype	Gelsolin
11	*RPGR*$^{ex1-19}$ variant	diminished
26	*RPGR*$^{ex1-19}$ variant	diminished
31	*RPGR*$^{ex1-19}$ variant	diminished
23	*RPGR*$^{ex1-19}$ variant	diminished
30	*RPGR*$^{ex1-19}$ variant	diminished
9	*RPGR*$^{ex1-19}$ variant	diminished
16	*RPGR*$^{ex1-19}$ variant	diminished
15	*RPGR*$^{ex1-19}$ variant	diminished
22	*RPGR*^{ORF15} variant	diminished
27	*RPGR*^{ORF15} variant	diminished
19	*RPGR*^{ORF15} variant	diminished
4	*RPGR*^{ORF15} variant	diminished
18	*RPGR*^{ORF15} variant	diminished
7	*RPGR*^{ORF15} variant	diminished
17	*RPGR*^{ORF15} variant	no change
28	*RPGR*^{ORF15} variant	diminished
29	*RPGR*^{ORF15} variant	no change

Supplemental Figure 13. *RPGR* defect led to diminished apical gelsolin. (A) 4-week *RPGR* KO HNC MCCs showed diminished apical gelsolin. Scale bar, 2 μm. **(B)** The diminished apical gelsolin was observed in the 4-week MCCs from RP patients with pathological *RPGR* variants. Scale bar, 5 μm. **(C)** A summary of Vangl1 distribution in the MCCs of patients with variants in *RPGR*.

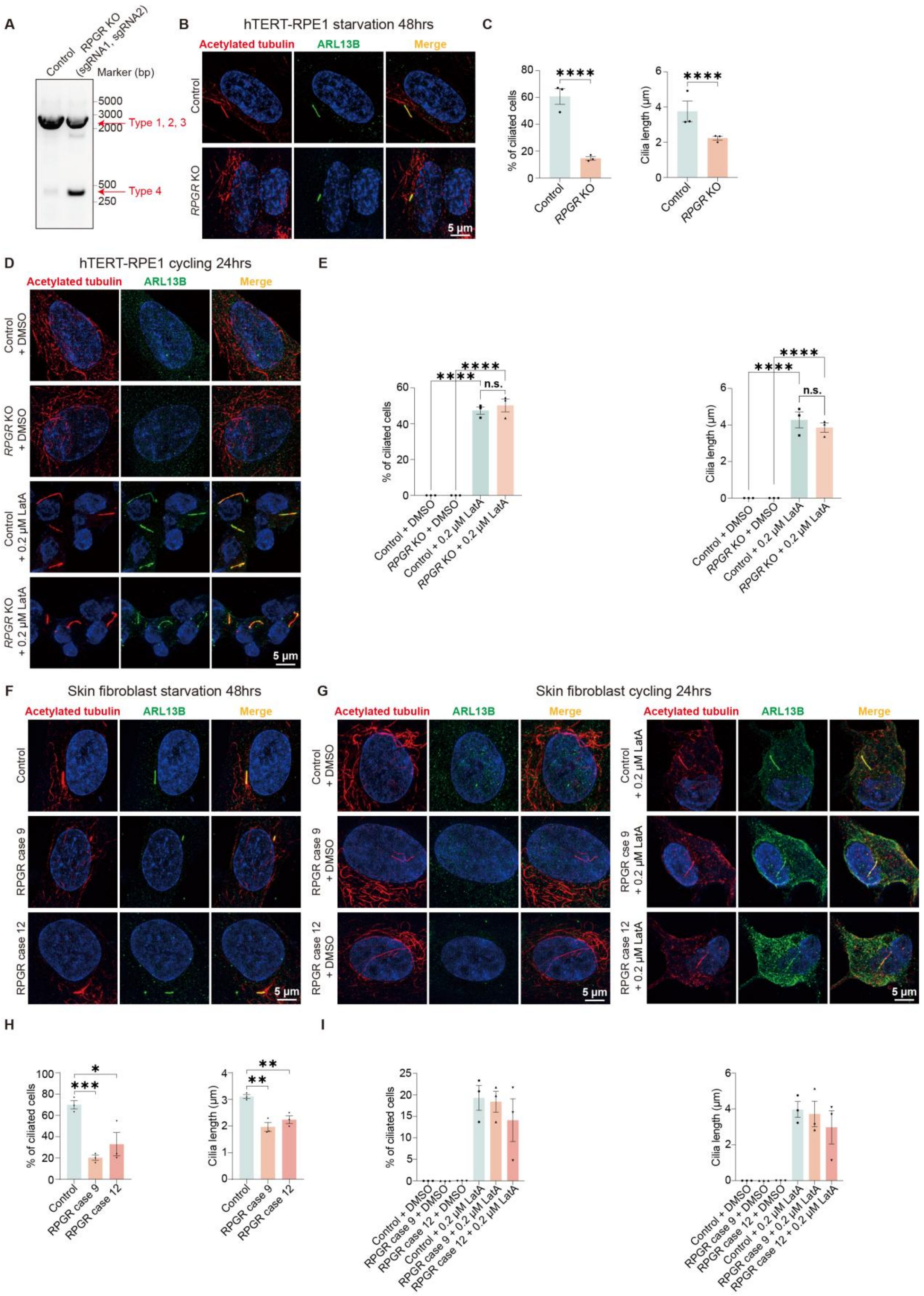

Supplemental Figure 14. Cilia issues in *RPGR* KO hTERT-RPE1 were rescued with LatA treatment. (A) DNA gel showed the high efficiency of *RPGR* KO in hTERT-RPE1. **(B, C)**

Loss of *RPGR* in hTERT-RPE1 cells led to reduced ciliation and short cilia length. Results were summarized from 3 technical replicates. n>30 cells for each technical replicate, and each point represents one technical replicate. Scale bar, 5 μm. **(D, E)** The ciliation and cilia length issues of *RPGR* KO hTERT-RPE1 cells were rescued by Lat A treatment. Scale bar, 5 μm. Results were summarized from 3 technical replicates. n>36 cells for each technical replicate, and each point represents one technical replicate. Scale bar, 5 μm. **(F, H)** Skin fibroblasts with pathological variants in *RPGR* (cases 9, and 12) showed reduced ciliation and cilia length. Results were summarized from 3 technical replicates. n>20 cells for each technical replicate, and each point represents one technical replicate. Scale bar, 5 μm. **(G, I)** Cilia issues of patient fibroblast cells caused by *RPGR* variants (cases 9, and 12) were rescued by LatA. Results were summarized from 3 technical replicates. n>32 cells for each technical replicate, and each point represents one technical replicate. Scale bar, 5 μm. Data represent mean ± SEM. n.s. no significance, *, $p < 0.05$, **, $p < 0.01$, ***, $p < 0.001$, ****, $p < 0.0001$ by two-tailed t-test **(C, E, H, I)**.

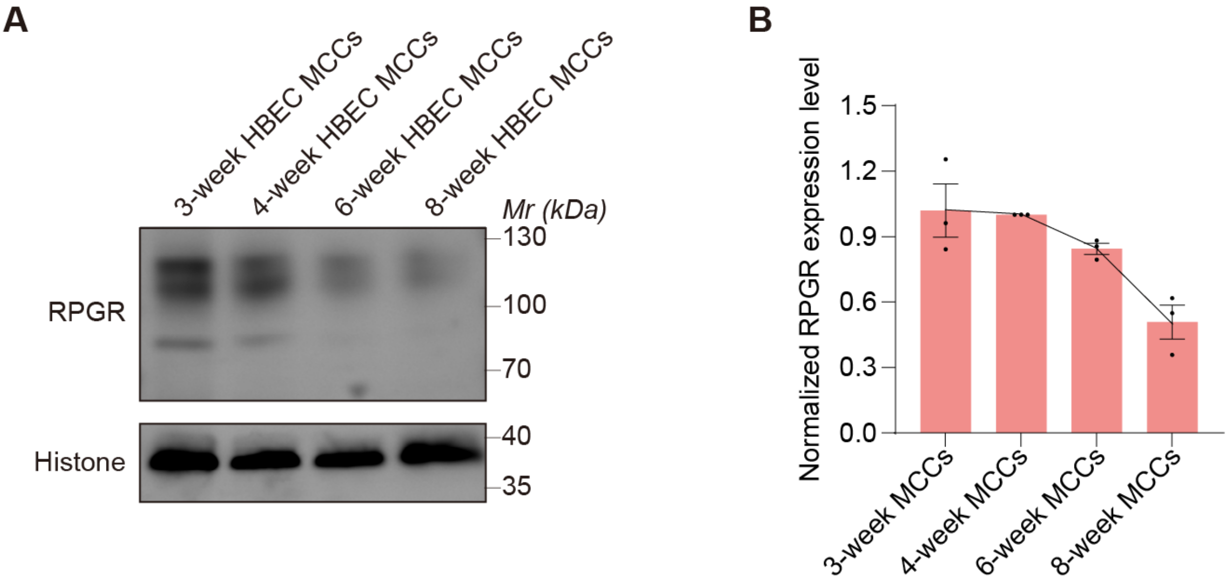

Supplemental Figure 15. RPGR expression at different developmental stages of MCCs. Western blot and quantification showed the changes in RPGR expression during the maturation of human bronchial epithelial cells. Results were summarized from 3 technical replicates. Each point represents one technical replicate. All data are presented as average ± SEM.

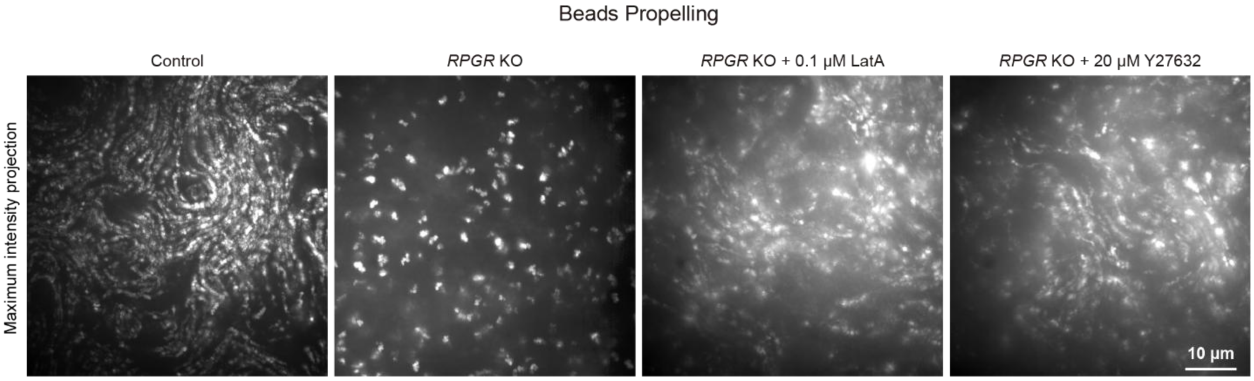

Supplemental Figure 16. LatA and Y27632 treatment ameliorated the mucociliary clearance defect caused by *RPGR* loss of function. The beads propelling experiment showed that both LatA and Y27632 treatment enhanced bead clearance. Scale bar, 10 μm.

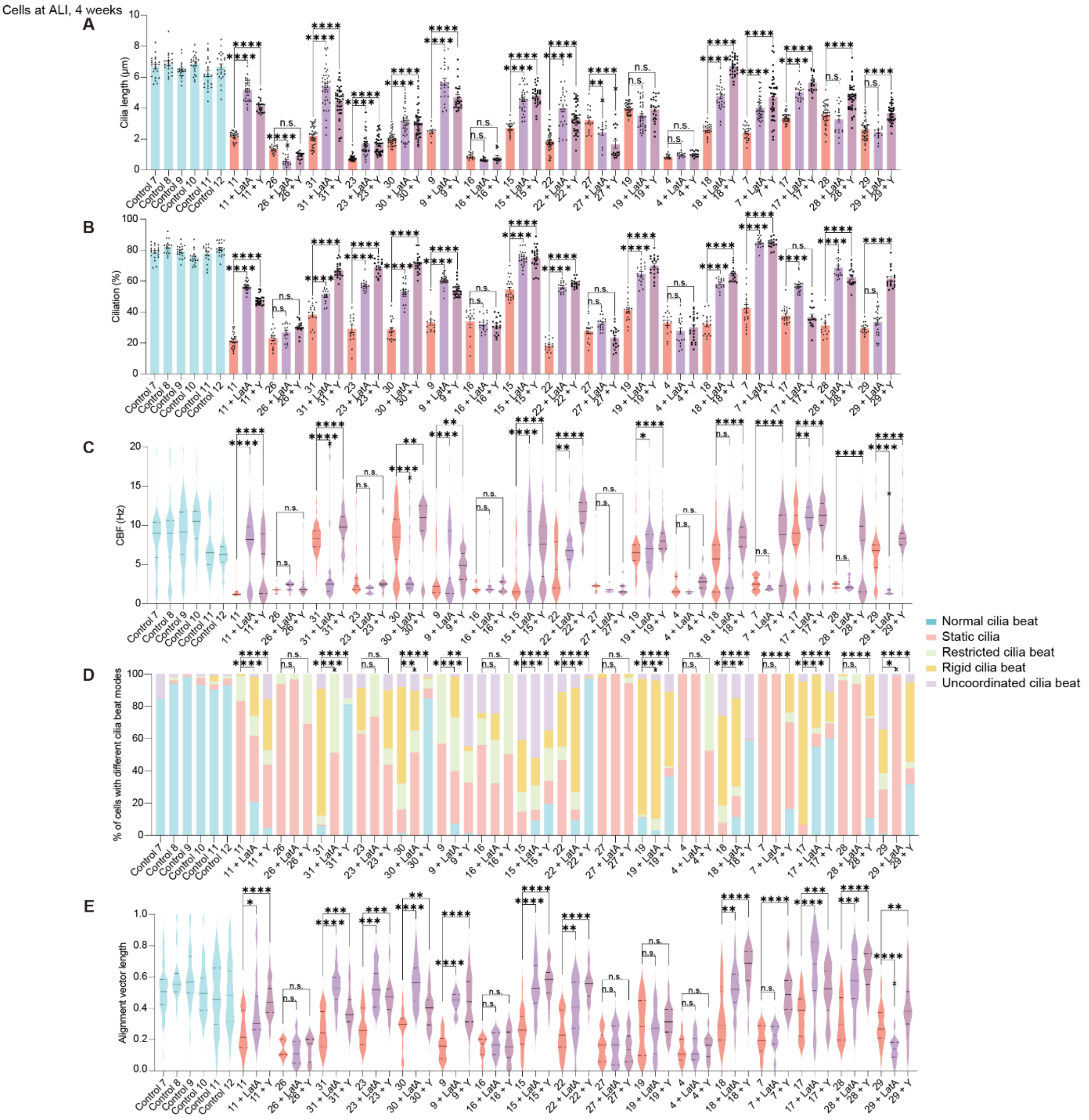

F

#	Genotype	Rescue	Rescue effect		
			Vangl1	Gelsolin	Beads propelling
11	*RPGR*$^{ex1-19}$ variant	LatA	no change	no change	++
		Y27632	+	++	+
26	*RPGR*$^{ex1-19}$ variant	LatA	no change	no change	no change
		Y27632	no change	no change	no change
31	*RPGR*$^{ex1-19}$ variant	LatA	no change	++	no change
		Y27632	no change	++	++
23	*RPGR*$^{ex1-19}$ variant	LatA	+	++	no change
		Y27632	+	++	+
30	*RPGR*$^{ex1-19}$ variant	LatA	+	×	no change
		Y27632	++	+	+
9	*RPGR*$^{ex1-19}$ variant	LatA	no change	no change	++
		Y27632	++	++	+
16	*RPGR*$^{ex1-19}$ variant	LatA	no change	no change	no change
		Y27632	no change	no change	no change
15	*RPGR*$^{ex1-19}$ variant	LatA	×	no change	+
		Y27632	+	no change	no change

#	Genotype	Rescue	Rescue effect		
			Vangl1	Gelsolin	Beads propelling
22	*RPGR*^{ORF15} variant	LatA	+	+	+
		Y27632	++	++	+++
27	*RPGR*^{ORF15} variant	LatA	no change	++	no change
		Y27632	no change	+	no change
19	*RPGR*^{ORF15} variant	LatA	no change	no change	no change
		Y27632	++	+	no change
4	*RPGR*^{ORF15} variant	LatA	no change	no change	no change
		Y27632	no change	no change	no change
18	*RPGR*^{ORF15} variant	LatA	no change	no change	no change
		Y27632	no change	+++	++
7	*RPGR*^{ORF15} variant	LatA	no change	++	no change
		Y27632	+	no change	+
17	*RPGR*^{ORF15} variant	LatA	no change	no change	no change
		Y27632	+++	+	+
28	*RPGR*^{ORF15} variant	LatA	+	++	+
		Y27632	+	++	++
29	*RPGR*^{ORF15} variant	LatA	no change	×	no change
		Y27632	+	no change	+

Supplemental Figure 17. LatA and Y27632 treatment partially rescued the MCCs from patients with pathological variants in *RPGR*. (A) Cilia length changes following LatA or

Y27632 treatment were evaluated in 17 patients. Improvements following LatA treatment were observed in 10 patient cells: 6 with *RPGR*$^{ex1\text{-}19}$ and 4 with *RPGR*^{ORF15} variants. Y27632 treatment resulted in improvements in 12 patient cells, with 6 having *RPGR*$^{ex1\text{-}19}$ and 6 having *RPGR*^{ORF15} variants. **(B)** Ciliation level assessments revealed improvements in 12 patient cells after Lat A treatment (6 *RPGR*$^{ex1\text{-}19}$ and 6 *RPGR*^{ORF15}) and in 12 patient cells after Y27632 treatment (6 *RPGR*$^{ex1\text{-}19}$ and 6 *RPGR*^{ORF15}). **(C)** Cilia beat frequency analysis indicated that 6 patient cells improved after LatA treatment (3 *RPGR*$^{ex1\text{-}19}$ and 3 *RPGR*^{ORF15}), whereas 12 patient cells showed improvement following Y27632 treatment (5 *RPGR*$^{ex1\text{-}19}$ and 7 *RPGR*^{ORF15}). **(D)** Cilia beat waveform improvements were observed in 6 patient cells post LatA treatment, with an increase in normal beating in 3 *RPGR*$^{ex1\text{-}19}$ and 3 *RPGR*^{ORF15} variants. After Y27632 treatment, 11 patient cells demonstrated improved cilia beat waveform (4 *RPGR*$^{ex1\text{-}19}$ and 7 *RPGR*^{ORF15}). **(E)** Coordination of cilia beats improved in 10 patient cells after LatA treatment (6 *RPGR*$^{ex1\text{-}19}$ and 4 *RPGR*^{ORF15}) and in 12 patient cells following Y27632 treatment (6 *RPGR*$^{ex1\text{-}19}$ and 6 *RPGR*^{ORF15}). **(F)** Changes in the distribution of Vangl1 and gelsolin, as well as the beads propelling efficiency after LatA or Y27632 treatment. The symbols convey treatment effects: “x” means worse compared to no treatment, “+” means improvement, “++” or “+++” means substantial improvements. Data represent mean ± SEM. The center, upper, and lower lines represent the median, upper, and lower quartiles, respectively **(C, E)**. n.s., no significance, *, $p < 0.05$, **, $p < 0.01$, ***, $p < 0.001$, ****, $p < 0.0001$ by two-tailed t-test **(A, B, C, E)**, or Fisher’s exact test **(D)**.

567

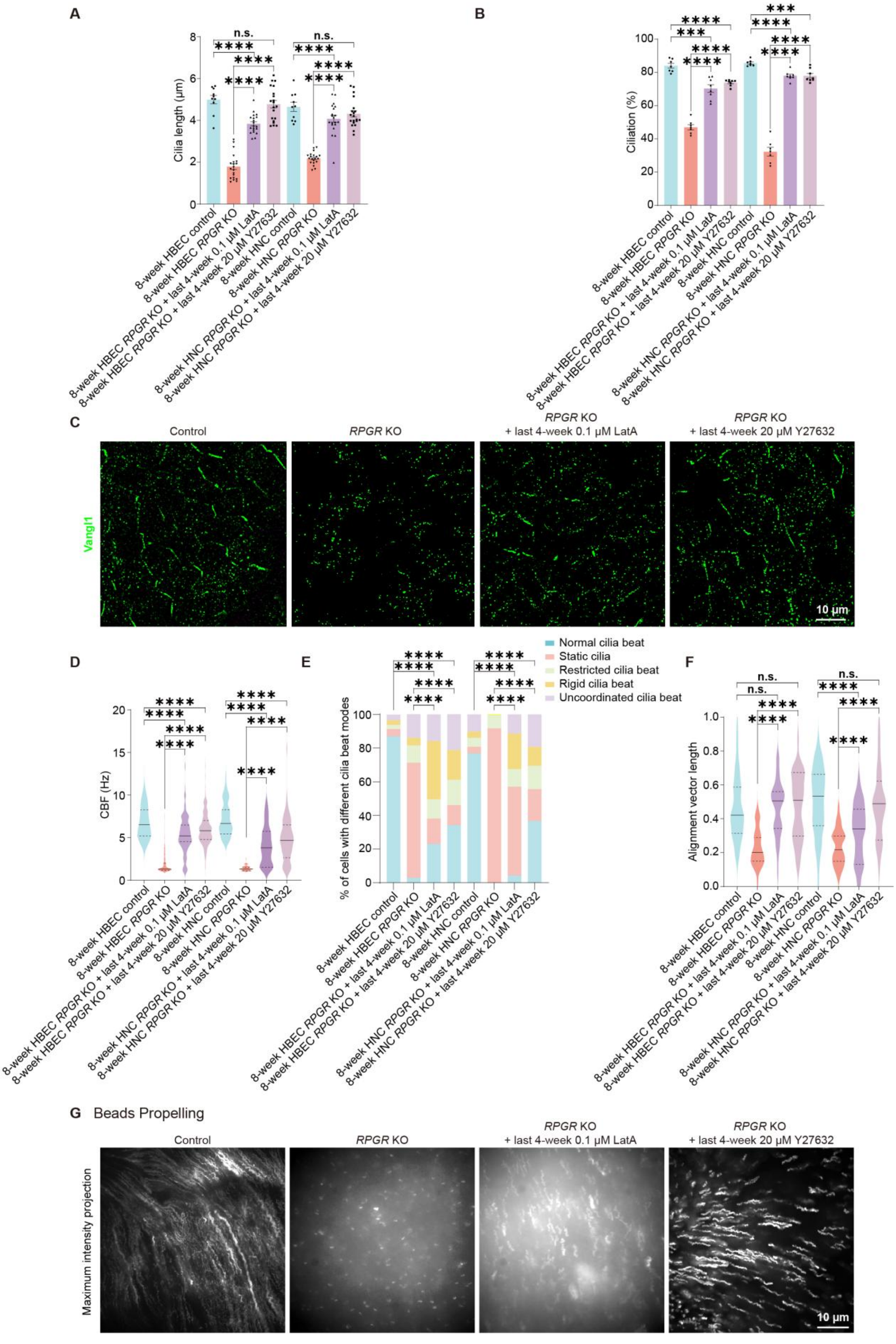

A
B
C
D
E
F
G
Beads Propelling
Cilia length (μm)
Ciliation (%)
CBF (Hz)
% of cells with different cilia beat modes
Alignment vector length
Normal cilia beat
Static cilia
Restricted cilia beat
Rigid cilia beat
Uncoordinated cilia beat
Control
RPGR KO
RPGR KO + last 4-week 0.1 μM LatA
RPGR KO + last 4-week 20 μM Y27632
Vangl1
Maximum intensity projection
10 μm
8-week HBEC control
8-week HBEC RPGR KO
8-week HBEC RPGR KO + last 4-week 0.1 μM LatA
8-week HBEC RPGR KO + last 4-week 20 μM Y27632
8-week HNC control
8-week HNC RPGR KO
8-week HNC RPGR KO + last 4-week 0.1 μM LatA
8-week HNC RPGR KO + last 4-week 20 μM Y27632
n.s.
****
***

Supplemental Figure 18. LatA and Y27632 treatment starting from ALI 4 weeks also ameliorated the motile cilia defect caused by *RPGR* KO. LatA and Y27632 were added into the culturing medium starting from ALI week 4, and the phenotypes were evaluated at ALI week 8. **(A, B)** Image quantification showed improvements in cilia length and ciliation for two *RPGR* KO biological replicates. **(C)** Y27632 and LatA restored the polarized distribution of Vangl1 at the apical surface in the 8-week HBEC sample. Scale bar, 10 μm. **(D-F)** The cilia beat anomalies in *RPGR* KO MCCs were partially rescued by either LatA or Y27632 treatment. **(D)** Both treatments improved cilia beat frequency. **(E)** Both treatments partially rescued the waveform, with more cells displaying a normal beating pattern. **(F)** The disrupted cilia beat coordination caused by *RPGR* KO was partially rescued or restored by LatA and Y27632 treatment. **(G)** Beads propelling experiment showed that both LatA and Y27632 treatment enhanced bead clearance. Scale bar, 10 μm. Results were summarized from 2 biological replicates (1 HBEC sample and 1 HNC sample). Data represent mean ± SEM. The center, upper, and lower lines represent the median, upper, and lower quartiles, respectively **(D, F)**. n.s., no significance, ***, $p < 0.001$, ****, $p < 0.0001$ by two-tailed t-test **(A, B, D, F)**, or Fisher's exact test **(E)**.

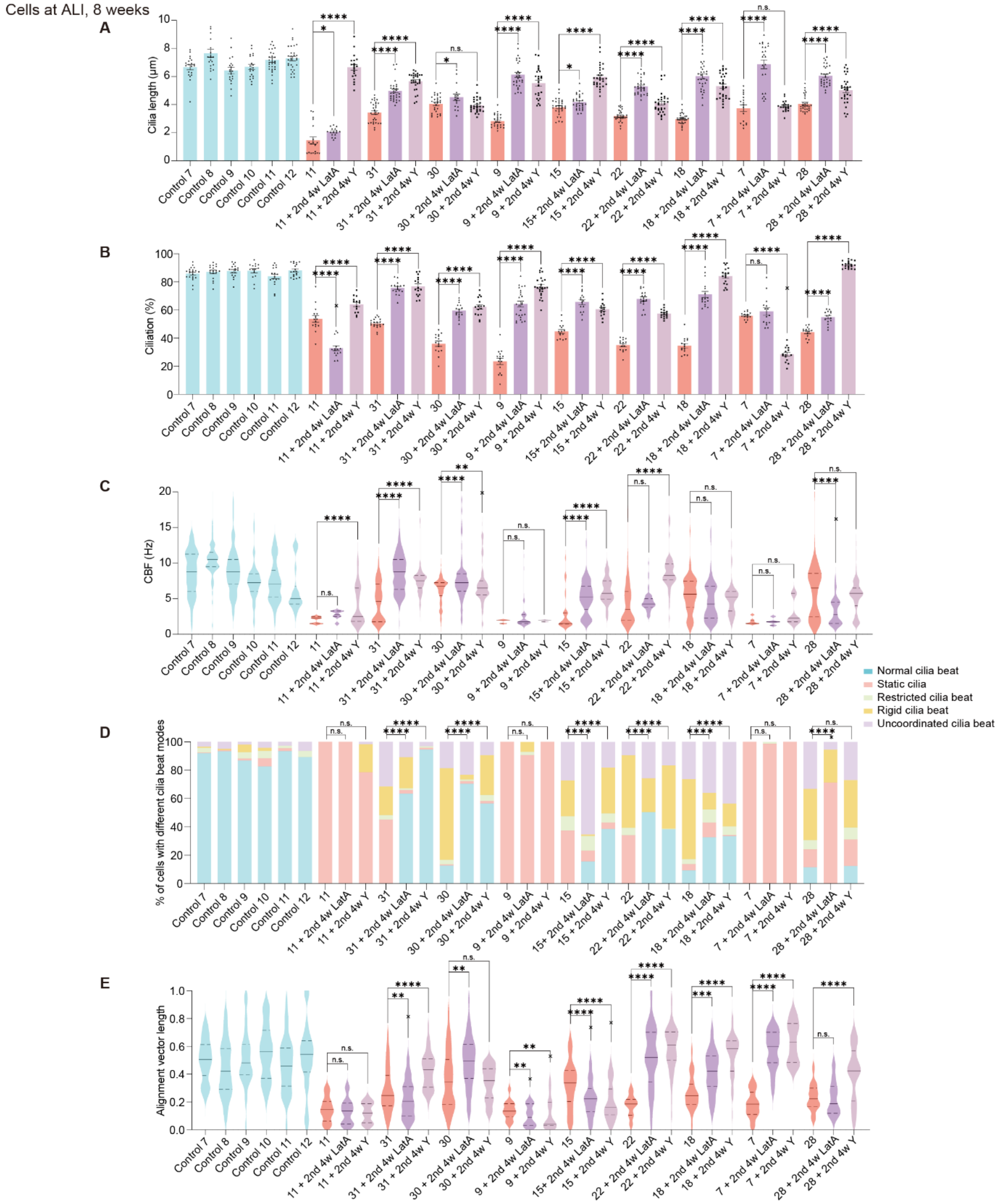

F Late rescue (Compared with 8-week RP samples)

#	Genotype	Rescue	Rescue effect	
			Vangl1	Beads propelling
11	$RPGR^{ex1-19}$ variant	LatA	no change	no change
		Y27632	no change	no change
31	$RPGR^{ex1-19}$ variant	LatA	×	++
		Y27632	no change	+
30	$RPGR^{ex1-19}$ variant	LatA	+	no change
		Y27632	+	no change
9	$RPGR^{ex1-19}$ variant	LatA	+	no change
		Y27632	+	no change
15	$RPGR^{ex1-19}$ variant	LatA	+	no change
		Y27632	++	+

#	Genotype	Rescue	Rescue effect	
			Vangl1	Beads propelling
22	$RPGR^{ORF15}$ variant	LatA	++	+
		Y27632	++	++
18	$RPGR^{ORF15}$ variant	LatA	+	no change
		Y27632	++	+
7	$RPGR^{ORF15}$ variant	LatA	+	no change
		Y27632	no change	no change
28	$RPGR^{ORF15}$ variant	LatA	no change	no change
		Y27632	+	+

Supplemental Figure 19. LatA and Y27632 treatment starting from ALI 4 weeks also ameliorated the motile cilia defect in patient MCCs. LatA and Y27632 were added to the culturing medium starting from ALI week 4, and the phenotypes were evaluated at ALI week 8. Changes in cilia properties after LatA or Y27632treatment were assessed in a total of 9 patients. **(A)** Cilia length improvements following LatA treatment were observed in 9 patient cells: 5 with *RPGR*$^{ex1\text{-}19}$ and 4 with *RPGR*^{ORF15} variants. For Y27632 treatment, improvements were found in 7 patients, including 4 with *RPGR*$^{ex1\text{-}19}$ and 3 with *RPGR*^{ORF15} variants. **(B)** Assessment of ciliation level indicated that, after LatA treatment, 7 patient cells showed improvements (4 *RPGR*$^{ex1\text{-}19}$ and 3 *RPGR*^{ORF15}). After Y27632 treatment, 8 patient cells showed improvements (5 *RPGR*$^{ex1\text{-}19}$ and 3 *RPGR*^{ORF15}). **(C)** Analysis of the cilia beat frequency map showed that 3 patient cells improved after LatA treatment (3 *RPGR*$^{ex1\text{-}19}$), while 5 patient cells showed improvements after Y27632 treatment (4 *RPGR*$^{ex1\text{-}19}$ and 1 *RPGR*^{ORF15}). **(D)** Cilia beat waveform analysis indicated that 5 patient cells showed an increase in normal cilia beat following LatA treatment (3 *RPGR*$^{ex1\text{-}19}$ and 2 *RPGR*^{ORF15}). After Y27632 treatment, 5 patient cells exhibited increased normal cilia beat (3 *RPGR*$^{ex1\text{-}19}$ and 2 *RPGR*^{ORF15}). **(E)** Coordination of cilia beats improved in 4 patient cells after LatA treatment (1 *RPGR*$^{ex1\text{-}19}$ and 3 *RPGR*^{ORF15}), while 5 patient cells showed enhanced coordination after Y27632 treatment (1 *RPGR*$^{ex1\text{-}19}$ and 4 *RPGR*^{ORF15}). **(F)** Changes in Vangl1 and gelsolin distribution, as well as cilia propelling efficiency after LatA or Y27632 treatment. The symbols convey treatment effects: "x" means worse relative to treatment, "+" means improvement, "++" or "+++" means substantial improvement. Data represent mean ± SEM. The center, upper, and lower lines represent the median, upper, and lower quartiles, respectively **(C, E)**. n.s., no significance, *, $p < 0.05$, **, $p < 0.01$, ***, $p < 0.001$, ****, $p < 0.0001$ by two-tailed t-test **(A, B, C, E)**, or Fisher's exact test **(D)**.

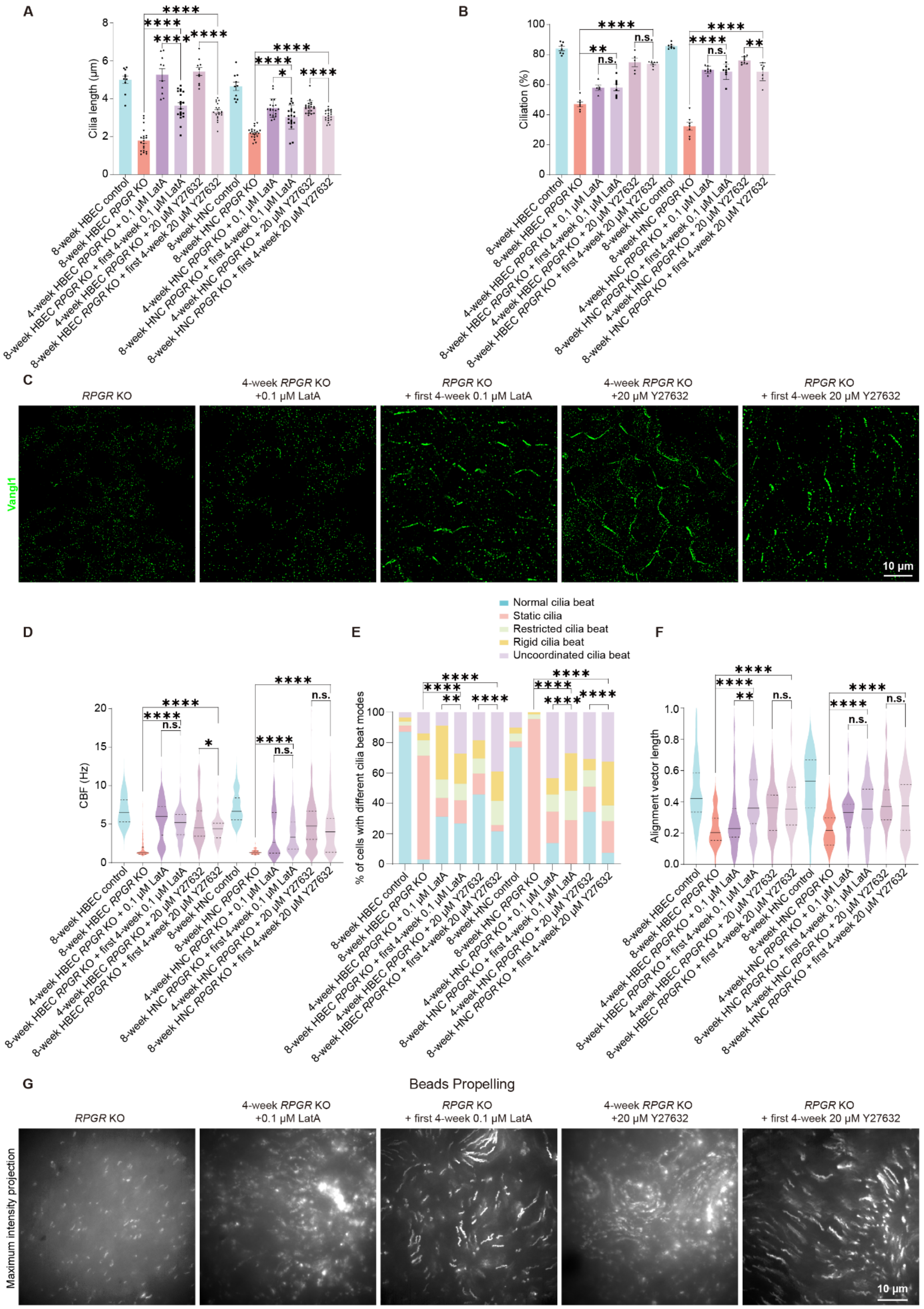

Supplemental Figure 20. Withdrawal of LatA and Y27632 treatment led to a decline in cilia properties but mucociliary clearance was preserved. LatA and Y27632 were administered in the culture medium from day 0 of ALI culturing, and the treatment was stopped

after 4 weeks. Phenotypes were evaluated at both weeks 4 and 8 for comparison. **(A)** Image quantification showed a significant decrease in cilia length following the withdrawal of LatA and Y27632 across two biological replicates. **(B)** Image quantification showed ciliation was either unaffected or slightly impacted after treatment withdrawal in two *RPGR* KO biological replicates. **(C)** The withdrawal of Y27632 and LatA didn't alter the polarized distribution of Vangl1 at the apical surface in the 8-week HBEC sample. Scale bar, 10 μm. **(D)** The withdrawal of LatA and Y27632 didn't significantly affect or only slightly decreased cilia beat frequency. **(E)** The withdrawal of LatA and Y27632 significantly worsened the waveform, with few cells displaying a normal beating pattern. **(F)** The withdrawal of LatA and Y27632 had a minor or negligible effect on cilia beat coordination. **(G)** Beads propelling experiment showed that bead clearance remained preserved after the withdrawal of either LatA or Y27632. Scale bar, 10 μm. For comparison, the 8-week *RPGR* KO data are the same as those in Supplemental Figure 18. The data for the 4-week patient samples rescued with LatA or Y27632 are a small subset of those in Figure 7 and Supplemental Figure 16. Results were from 2 biological replicates. Data represent mean ± SEM. The center, upper, and lower lines represent the median, upper, and lower quartiles, respectively **(D, F)**. n.s., no significance, *, $p < 0.05$, **, $p < 0.01$, ****, $p < 0.0001$ by two-tailed t-test **(A, B, D, F)**, or Fisher's exact test **(E)**.

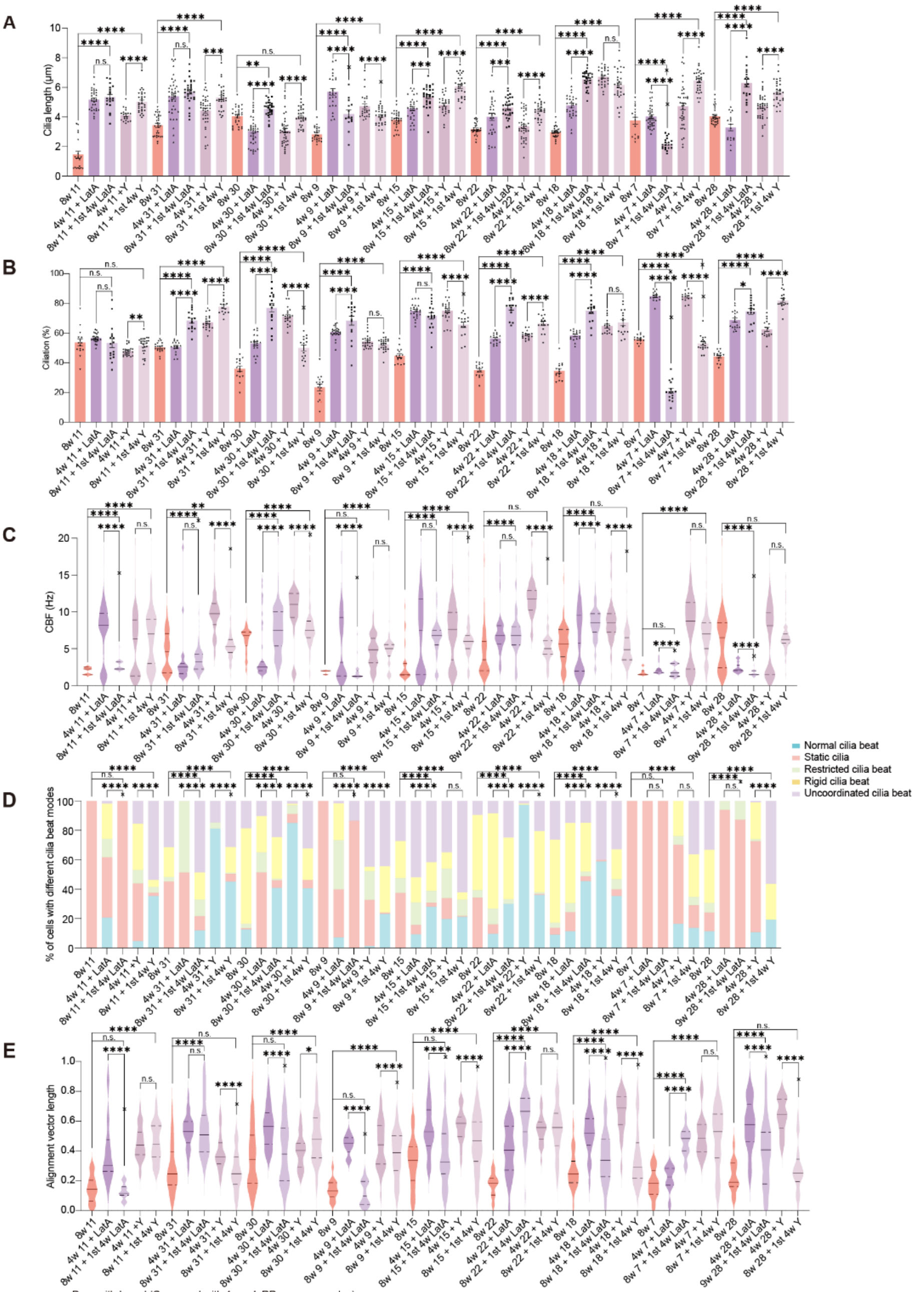

F

Drug withdrawal (Compared with 4-week RP rescue samples)

#	Genotype	Rescue	Rescue effect	
			Vangl1	Beads propelling
11	*RPGR*$^{ex1-19}$ variant	LatA	no change	no change
		Y27632	+	+
31	*RPGR*$^{ex1-19}$ variant	LatA	×	+
		Y27632	++	+
30	*RPGR*$^{ex1-19}$ variant	LatA	+	no change
		Y27632	+	+
9	*RPGR*$^{ex1-19}$ variant	LatA	+	×
		Y27632	++	no change
15	*RPGR*$^{ex1-19}$ variant	LatA	+	no change
		Y27632	+	++

#	Genotype	Rescue	Rescue effect	
			Vangl1	Beads propelling
22	*RPGR*^{ORF15} variant	LatA	++	+
		Y27632	+	++
18	*RPGR*^{ORF15} variant	LatA	no change	+
		Y27632	no change	+
7	*RPGR*^{ORF15} variant	LatA	no change	no change
		Y27632	+	×
28	*RPGR*^{ORF15} variant	LatA	×	+
		Y27632	×	no change

G

Drug withdrawal (Compared with 8-week RP samples)

#	Genotype	Rescue	Rescue effect	
			Vangl1	Beads propelling
11	*RPGR*$^{ex1-19}$ variant	LatA	+	no change
		Y27632	+++	+
31	*RPGR*$^{ex1-19}$ variant	LatA	×	+
		Y27632	no change	+++
30	*RPGR*$^{ex1-19}$ variant	LatA	+	+
		Y27632	+	++
9	*RPGR*$^{ex1-19}$ variant	LatA	+	no change
		Y27632	+++	+
15	*RPGR*$^{ex1-19}$ variant	LatA	+	+
		Y27632	+++	+++

#	Genotype	Rescue	Rescue effect	
			Vangl1	Beads propelling
22	*RPGR*^{ORF15} variant	LatA	++	++
		Y27632	++	++
18	*RPGR*^{ORF15} variant	LatA	+	+
		Y27632	+	++
7	*RPGR*^{ORF15} variant	LatA	no change	no change
		Y27632	++	no change
28	*RPGR*^{ORF15} variant	LatA	no change	+
		Y27632	+	+

Supplemental Figure 21. Examining patient MCCs showed that withdrawal of LatA and Y27632 led to a decline in cilia properties, but mucociliary clearance was preserved. LatA and Y27632 were administered in the culture medium from day 0 of ALI culturing, and the treatment was stopped after 4 weeks. Phenotypes were evaluated at both 4 and 8 weeks for comparison. Changes in cilia properties following drug withdrawal were assessed in a total of 9 patients. **(A)** Cilia length maintenance or improvement after LatA withdrawal was observed in 7 patient cells: 4 with $RPGR^{ex1\text{-}19}$ variants and 3 with $RPGR^{ORF15}$ variants. Similar maintenance or improvement was found after Y27632 withdrawal in 7 patient cells, including 4 with $RPGR^{ex1\text{-}19}$ variants and 3 with $RPGR^{ORF15}$ variants. **(B)** After LatA withdrawal, 2 patient cells showed a decrease in ciliation (1 $RPGR^{ex1\text{-}19}$ and 1 $RPGR^{ORF15}$). After Y27632 withdrawal, 3 patient cells showed a decrease (2 $RPGR^{ex1\text{-}19}$ and 1 $RPGR^{ORF15}$); **(C)** Cilia beat frequency analysis showed a decrease in 5 patient cells after LatA withdrawal (3 $RPGR^{ex1\text{-}19}$ and 2 $RPGR^{ORF15}$). A similar decrease was observed in 5 patient cells following Y27632 withdrawal (3 $RPGR^{ex1\text{-}19}$ and 2 $RPGR^{ORF15}$). **(D)** Cilia beat waveform analysis revealed that 4 patient cells showed a decrease in normal cilia beat after LatA withdrawal (2 $RPGR^{ex1\text{-}19}$ and 2 $RPGR^{ORF15}$). Following Y27632 withdrawal, 5 patient cells showed a decrease in normal cilia beat (2 $RPGR^{ex1\text{-}19}$ and 3 $RPGR^{ORF15}$). **(E)** Coordination of cilia beats deteriorated in 8 patient cells after LatA withdrawal (4 $RPGR^{ex1\text{-}19}$ and 4 $RPGR^{ORF15}$), while 5 patient cells showed affected coordination after Y27632 withdrawal (3 $RPGR^{ex1\text{-}19}$ and 2 $RPGR^{ORF15}$). **(F and G)** Changes in the distribution of Vangl1 and gelsolin, as well as cilia propelling efficiency, were observed following drug withdrawal. The symbols convey treatment effects: "x" indicates worse compared to no treatment, "+" indicates improvement, and "++" and "+++" indicate substantial improvement. For comparison, the 8-week patient data are the same as those in Supplemental Figure 19. The data for the 4-week patient samples rescued with LatA or Y27632 are a small subset of those in Figure 8 and Supplemental Figure 17. Data represent mean ± SEM. The center, upper, and lower lines represent the median, upper, and lower quartiles, respectively (**C**, **E**). n.s., no significance, *, $p < 0.05$, **, $p < 0.01$, ***, $p < 0.001$, ****, $p < 0.0001$ by two-tailed t-test (**A, B**, **C**, **E**), or Fisher's exact test (**D**).
