# Supplementary material for: Loss of RPGR disrupts motile cilia and causes primary ciliary dyskinesia by affecting F-actin dynamics
Source: J Clin Invest. 2026 Mar 31;136(10):e193367. doi: 10.1172/JCI193367 (PMC13178666; doi:10.1172/JCI193367)
Supplement: Unedited blot and gel images [file jci-136-193367-s108.pdf]

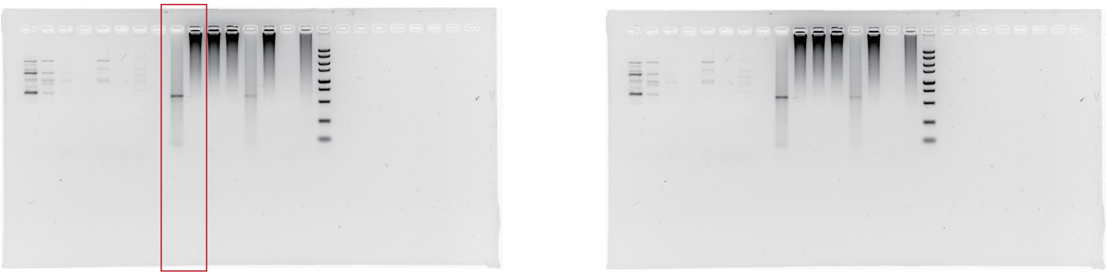

Figure 2J. RT-PCR showed the expression of the ORF15 isoform in human airway epithelial cells.

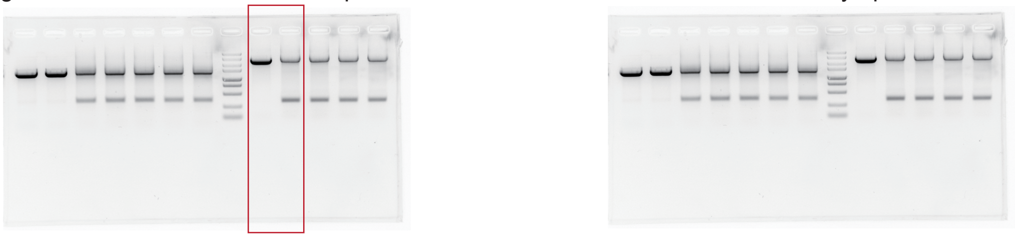

Figure 3C. DNA gel and Sanger sequencing showed high efficiency of *RPGR* KO in MCCs.

Ms-actin

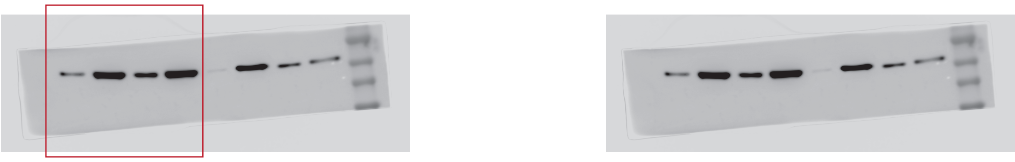

Figure 6D. WB showed that the increased F-actin ratio for 4-week *RPGR* KO samples.

Ms-actin

Ms-actin

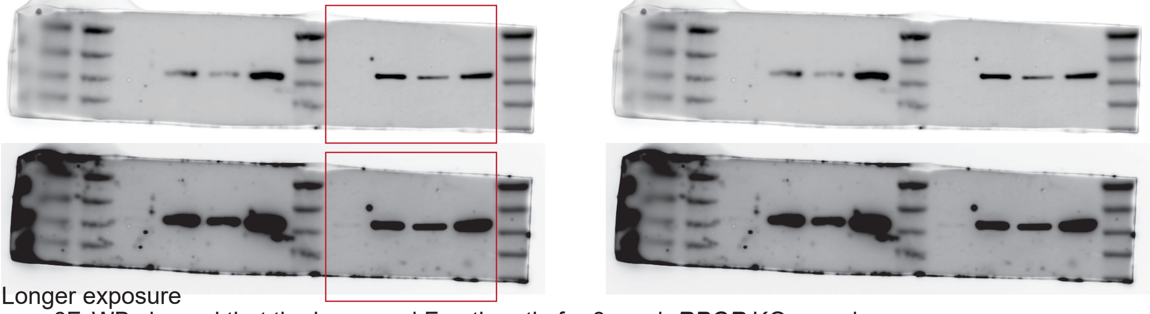

Longer exposure

Figure 6F. WB showed that the increased F-actin ratio for 8-week *RPGR* KO samples.

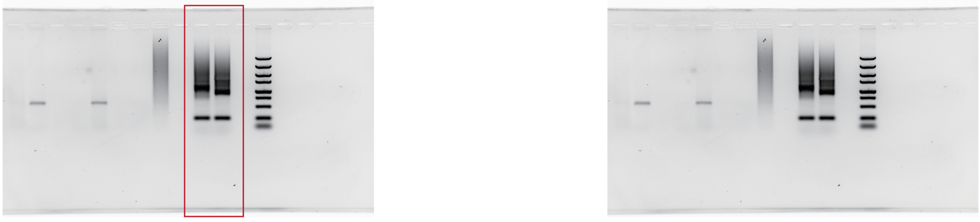

Supplemental Figure 5B Loading control for RT-PCR shown in Fig. 2J.

Ms-actin

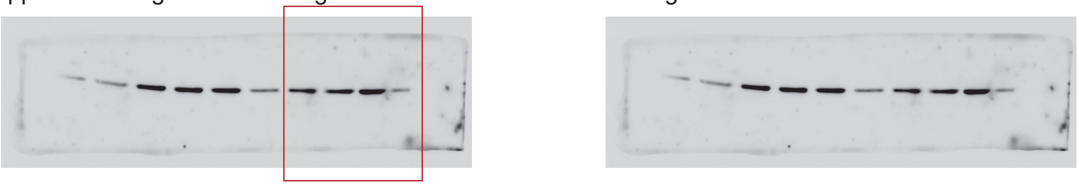

Supplemental Figure 12A. WB showed the increased F-actin ratio due to the treatment of Rho Activator II.

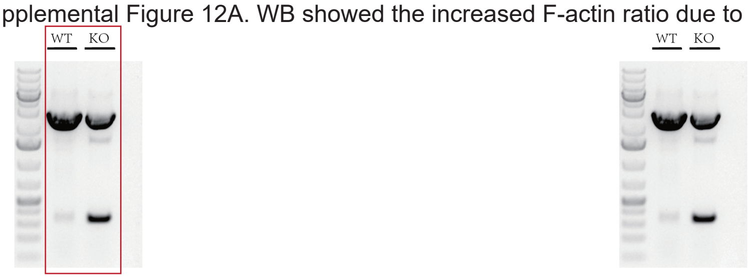

Supplemental Figure 14A. DNA gel showed the high efficiency of *RPGR* KO in hTERT-RPE1.

Rbt-RPGR

Rbt-Histone H1

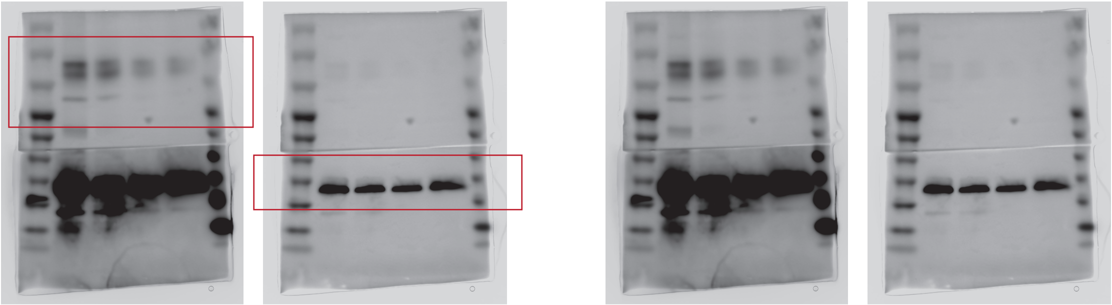

Supplemental Figure 15. *RPGR* expresion change during ciliogenesis.
